# Proteomic Profiling of Cryoglobulinemia

**DOI:** 10.3389/fimmu.2022.855513

**Published:** 2022-05-23

**Authors:** Peng Liu, Jianqiang Wu, Dandan Sun, Haolong Li, Zhihong Qi, Xiaoyue Tang, Wei Su, Yongzhe Li, Xuzhen Qin

**Affiliations:** ^1^ Medical Research Center, State Key Laboratory of Complex Severe and Rare Diseases, Peking Union Medical College Hospital, Chinese Academy of Medical Sciences and Peking Union Medical College, Beijing, China; ^2^ Department of Laboratory Medicine, Chinese Academy of Medical Sciences and Peking Union Medical College Hospital, Beijing, China

**Keywords:** proteomics, serum, biomarker, cyroglobulinemia, metabolic pathway

## Abstract

**Objective:**

We aimed to explore and identify candidate protein biomarkers of cryoglobulinemia (CGE) in disease control patients with negative cryoglobulin (DC) or healthy controls (HCs).

**Methods:**

The tandem mass tag (TMT)-labeled serum quantitative proteomics approach was used to identify differentially expressed proteins between the CGE and DC groups. Ingenuity pathway analysis was used for functional annotation of differentially expressed proteins. Biomarker candidates were validated in another cohort using the parallel reaction monitoring (PRM) method. Apolipoprotein A1 (APOA1), apolipoprotein CIII (APOC3), adiponectin, and proprotein convertase subtilisin/kexin type-9 (PCSK9), which represent key proteins involved in the cholesterol metabolism pathway, were further verified in an increased number of samples by enzyme-linked immunosorbent assay (ELISA).

**Results:**

A total of 1004 proteins were identified, of which 109 proteins were differentially expressed between the CGE and DC groups. These differentially expressed proteins were primarily involved in hepatic fibrosis/hepatic stellate cell activation and immune/inflammation-related pathways. In the disease and biofunction analysis, these proteins were mainly associated with the adhesion of blood cells, leukocyte migration, cholesterol transport, and transport of lipids. Twelve candidate biomarkers were validated by PRM-based proteomics, and proteins involved in the cholesterol metabolism pathway were further verified. APOA1, APOC3, adiponectin and PCSK9 concentrations were increased in CGE patients compared with healthy controls (P=0.0123, 0.1136, 0.5760, and 0.0019, respectively).

**Conclusion:**

This report describes the first application of a TMT-PRM-ELISA workflow to identify and validate CGE-specific biomarkers in serum. APOA1 and PCSK9 have been confirmed to be increased in CGE patients, demonstrating that proteins involved in cholesterol metabolism are also implicated in the development of CGE. These findings contribute to pathogenesis research and biomarker discovery in CGE.

## Highlights:

1. This is the first report on applying the proteomics approach in cryoglobulinemia research in China.
2. This is the first report on the relationship between CGE development and cholesterol metabolism.

## Introduction

Cryoglobulinemia (CGE) is a group of clinical syndromes caused by the presence of cryoglobulin (CG) in serum. CG is a special immunoglobulin that precipitates at low temperatures and then dissolves at approximately 37°C. CG can be divided into three types based on different compositions. Type I is mainly composed of monoclonal IgM, type II is composed of two different types of polyclonal IgG and monoclonal IgM, and type III is composed of the combination of polyclonal IgG and polyclonal IgM. Type I is mainly noted in lymphoma and other hematological tumors. Type II and III are also known as mixed cryoglobulinemia (MC) because the CG in these types has various clonal components and rheumatoid factor (RF) activity. The clinical manifestations of MC are mainly multisystem vasculitis, which can affect small and medium-sized vessels (1). As the disease progresses, severe irreversible clinical manifestations, such as glomerulonephritis, peripheral neuropathy, and skin ulcers, occur. In addition, patients with MC have a poor response to treatment, high mortality rate, and a high sequelae rate of chronic organ failure (such as chronic renal failure and lymphoma). The 10-year survival rate was significantly reduced. Therefore, CGE is a disease with atypical symptoms and may cause severe damage to patients (2, 3).

Previous studies have suggested that CGE is a rare disease (less than 5 in 10,000 people in Europe and North America). However, the prevalence of CGE reaches 20% in some regions (4). As a concomitant disease, approximately 30-50% of hepatitis C virus (HCV)-infected patients, 10% of systemic lupus erythematosus patients, and 5-20% of primary Sjogren’s syndrome patients have MC (1). According to the epidemic situation of Statutory Reporting infectious diseases in China in 2018, hepatitis C infection was noted in more than 200,000 individuals (5). Therefore, the theoretical estimates of the prevalence of CGE in China are higher than those in other regions due to the relatively high prevalence of HCV in China. However, the diagnosis of CGE is always neglected due to the absence of any symptoms at the early stage or nonspecific clinical manifestations (greater than 60% present palpable purpura, arthralgia, and fatigue) (6). It is critical to identify accurate early diagnosis and risk assessment markers for the prognosis of patients.

Mass spectrometry (MS)-based proteomic analysis is a powerful screening tool for the rapid detection of disease biomarkers. In a previous study, MS was used to molecularly type IgM RFs in CG (7). The main protein components in a serum cryoprecipitate from a patient with HCV infection and presenting type II CGE were IgM and IgG as identified by MS analysis. MS analysis was also used to type CG based on their unique molecular barcodes (8). Using an MS-based proteomic approach, one study identified the immunoglobulin heavy chain variable region subfamilies and mutational profiles of cryoprecipitable IgM-RF in a group of primary Sjögren’s syndrome patients with MC along with their clonotypic heavy chain complementarity-determining regions 3 (HCDR3) peptides (9). However, all of these studies focused on the immunological characteristics of the cryoprecipitate. No previous studies have investigated the pathogenesis and proteomics of CGE using mass spectrometry analysis.

In this study, we first used tandem mass tag (TMT)-labeled MS technology to analyze the differential proteome in the serum of CGE patients and disease control patients with negative CG. Then, the candidate biomarkers were validated using parallel reaction monitoring (PRM) technology in an external set of samples, consisting of CGE patients, disease control patients (DC), and healthy controls (HC). Furthermore, immunoassays were applied to verify the levels of key proteins in the differentially expressed pathway. This study may provide helpful information for understanding CGE development and the findings of potential serum biomarkers.

## Methods

### Patients and Sample Collection

For the TMT-labeled quantitative proteomics analysis, ten CGE patients and ten matched DC patients were recruited in the same period from Peking Union Medical College Hospital (PUMCH) in China. These DC patients had similar clinical symptoms to CGE patients, but their CG tests were negative. For validation purposes, an independent case–control test set, including ten CGE, five HC, and five DC patients, was obtained from PUMCH and tested using the PRM method. Finally, 48 CGE patients and 32 HCs were enrolled to verify several key proteins using ELISA.

A total of 1 mL of serum sample after clinical measurement was collected for each subject and stored at −80°C. CG was qualitatively analyzed by capillary electrophoresis typing, and the quantitative analysis of CG was performed using the pyrogallol molybdenum-collateralization method in PUMCH (10, 11). In all cases, the blood cell count and the biochemical profile were reviewed. Clinical information was obtained from the Hospital Information System. The Institutional Ethics Committee at the PUMCH approved the study (No. S-K904). Informed written consent was waived for the remaining routine test samples.

### Sample Preparation

The cellular debris of the serum sample was removed by centrifugation at 12,000 g at 4°C for 10 min. The top 12 high abundance proteins were removed using a Pierce™ Top 12 Abundant Protein Depletion Spin Column Kit (Thermo Fisher). Then, the protein concentration was determined using the BCA method. The protein was digested using the FASP method (12). Briefly, the samples were reduced with 5 mM dithiothreitol for 30 min at 56°C and alkylated with 11 mM iodoacetamide for 15 min at room temperature in darkness. Finally, trypsin was added at a 1:50 trypsin-to-protein mass ratio overnight. After digestion, the peptide samples were desalted using a Strata X C18 SPE column (Phenomenex) and dried under vacuum.

### TMT-Labeled Quantitative Proteomic Analysis

Two units of TMT reagents were thawed and reconstituted in acetonitrile. Five randomly selected samples in each group were individually labeled with a 10-plex TMT reagent (Thermo Fisher Scientific). The TMT-labeled samples were pooled and then fractionated into fractions by high pH reverse-phase high-performance liquid chromatography. Briefly, peptides were first separated with a gradient of 8% to 32% acetonitrile (pH 9.0) over 60 min into 60 fractions. Then, the peptides were combined into six fractions and dried by vacuum centrifugation.

The tryptic peptides were dissolved in 0.1% formic acid (solvent A) and directly loaded onto a homemade reversed-phase analytical column (15-cm length, 75 μm i.d.). The gradient comprised an increase from 6% to 23% solvent B (0.1% formic acid in 98% acetonitrile) over 26 min, 23% to 35% in 8 min, an increase to 80% in 3 min, and a hold at 80% for the last 3 min. All steps were performed at a constant flow rate of 400 nL/min. Q Exactive™ Plus Mass spectrometry (Thermo Fisher Scientific) coupled with an EASY-nLC 1000 UPLC system was used for the MS analysis in the data-dependent acquisition mode. The m/z scan range was 350 to 1800 for a full scan, and intact peptides were detected in the Orbitrap at a resolution of 70,000. Peptides were then selected for MS/MS using the normalized collisional energy setting as 28, and the fragments were detected in the Orbitrap at a resolution of 17,500.

The resulting MS/MS data were processed using the Maxquant search engine (v.1.5.2.8). The mass tolerance for precursor ions was set as 20 ppm in the first search and 5 ppm in the main search, and the mass tolerance for fragment ions was set as 0.02 Da. The false discovery rate was adjusted to < 1%, and the minimum score for modified peptides was set to > 40. The protein abundance ratio was based on the unique peptide results. The relative quantification values of each sample made log2 changes to normalize the data, and then corresponding p values were tested. To be considered differentially expressed, proteins were required to have a p value < 0.05 and a fold change > 1.20.

### Parallel Reaction Monitoring (PRM) Proteomics Analysis

In PRM analysis, a Q Exactive™ Plus mass spectrometer was used to analyze eluted peptides from liquid chromatography. To ensure the quality of the data, the mixed sample was analyzed as a quality control to ensure the stability of the instrument signal during the entire process. The indexed retention time (iRT) standard peptide was also added to each sample, and the stability of the chromatographic retention time was assessed during the analysis. Two technical repeats were run in each sample. To reduce system bias, different groups of samples were analyzed in a random order for mass spectrometry analysis.

The gradient comprised an increase from 6% to 23% solvent B (0.1% formic acid in 98% acetonitrile) over 38 min, 23% to 35% in 14 min, an increase to 80% in 4 min, a hold at 80% for the last 4 min. All procedures were performed at a constant flow rate of 700 nL/min on an EASY-nLC 1000 UPLC system. The m/z scan range was 350 to 1000 for the full scan, and intact peptides were detected in the Orbitrap at a resolution of 35,000. Peptides were then selected for MS/MS using a normalized collisional energy setting of 27, and the fragments were detected in the Orbitrap at a resolution of 17,500.

The resulting MS data were processed using Skyline software (v.3.6). All data were imported into Skyline software, and the correct peaks were selected manually. The mass spectra of each peptide in each sample were normalized to the total ionic chromatography strength of the sample to adjust the errors from the sample loading amount and MS signal intensity. The results of each peptide were quantitatively analyzed, and the differentially expressed proteins between different groups were analyzed and compared with TMT results.

### Immunoassay Validation of APOA1, APOC3, Adiponectin and PCSK9 Concentrations

Serum samples of 48 CGE patients and 32 healthy controls matched by sex were collected from PUMCH. According to the manufacturer’s instructions, commercial ELISA kits were utilized to measure the concentrations of APOA1 (Abcam, ab189576), APOC3 (Abcam, ab154131), adiponectin (Abcam, ab222508), and PCSK9 (BioLegend, 443107). Briefly, serum samples were diluted with different factors based on individual biomarkers. The standard and sample were then added to ELISA plates. The antibody cocktail was added to the ELISA plates followed by incubation for 1 hour at room temperature. Each well was aspirated and washed, and development solution was added to each well and incubated. Stop solution was added at a defined endpoint, and the optical density was read at 450 nm using a microplate reader (Biotek, Winooski, CT, USA).

### Statistics

Pattern recognition analysis (principal component analysis, PCA; orthogonal partial least squares discriminant analysis, OPLS-DA) was performed using SIMCA 14.0 (Umetrics, Sweden) software. Comparison of the selected peptides between groups was analyzed using the t test when appropriate, and P < 0.05 was considered significant. For the Ingenuity Pathway Analysis (IPA), the SwissProt accession numbers were uploaded to IPA software (Ingenuity Systems, Mountain View, CA, United States). The proteins were mapped to the disease and function categories and canonical pathways available in ingenuity and were ranked by the P value. Receiver operating characteristic (ROC) analysis was performed using MedCalc software and the Metaboanalyst website (http://www.metaboanalyst.ca).

## Results

### Demographics of Included Patients

Samples from CGE and DC patients (negative for CG) and HCs were included in this study. The diagnosis in the DC group covered purpura, nephritis, and related diseases with similar symptoms. The patients were all negative for HCV and HIV antibodies. In the CGE patients and DC group, 20-40% of patients were positive for hepatitis B antigen. These three groups were age- and sex-matched.

**Figure 1:**
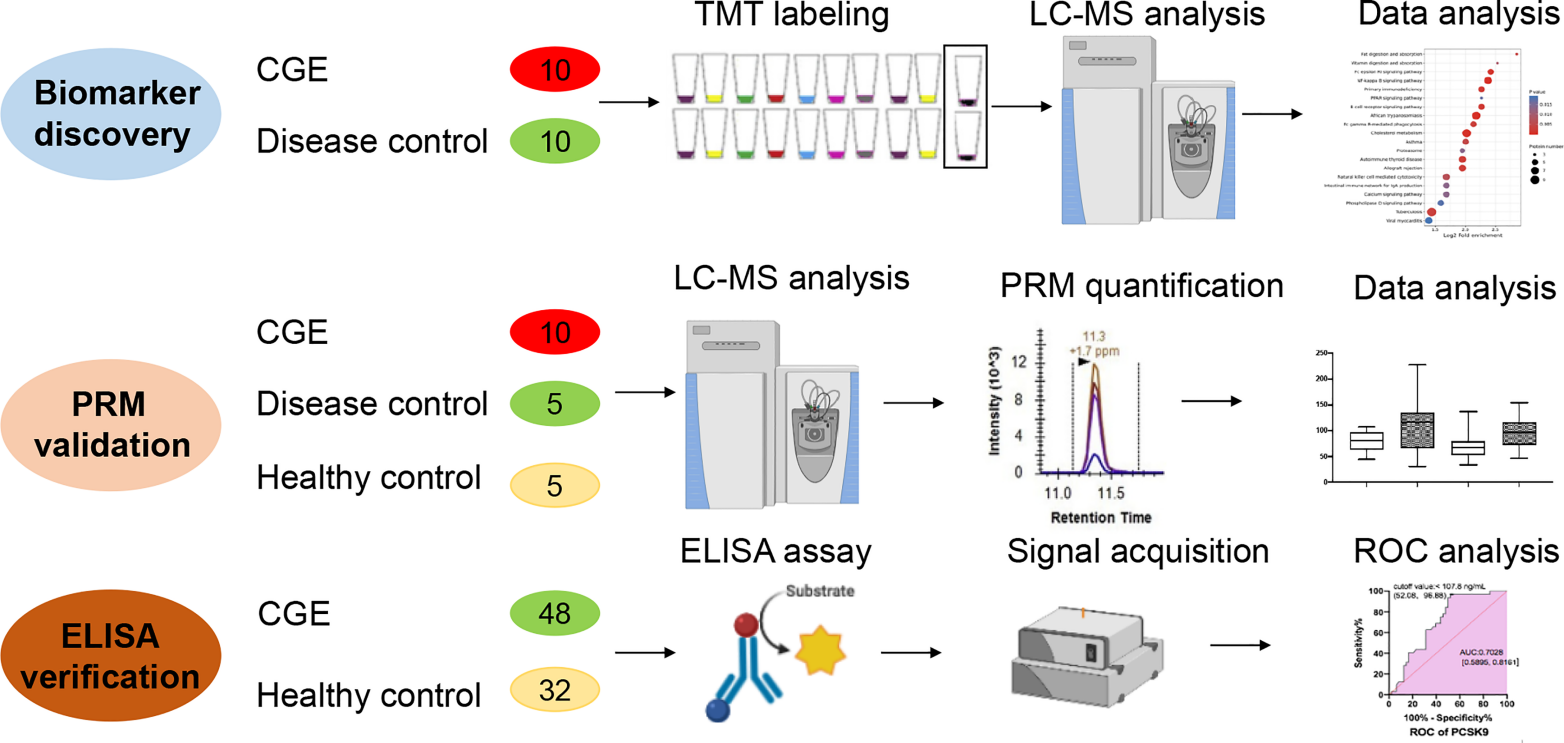
The workflow of this study.

The study design of this study is shown in **Figure 1**. In the discovery stage, serum samples from 10 CGE and 10 DC patients were randomly included in the discovery group and used for differential proteome discovery. In the validation stage, 10 CGE patients, 5 DC patients, and 5 HCs were included in the validation group and used for PRM validation. Then, 48 CGE patients and 32 HCs were included in the immunoassay. The characteristics of the patients are summarized in **Table 1** and **Table S1**.

**Table 1:** Clinical characteristics of enrolled subjects.

Analysis	Group	N	Age(mean±SD)	Gender(M/F)	Average CG quantity (g/L)
TMT-MS	Cryoglobulinemia	10	58.5 ± 9.4	5/5	1793.99
	Disease Control	10	56.9 ± 7.1	4/6	–
PRM-MS	Cryoglobulinemia	10	49.5 ± 10.5	5/5	305.00
	Disease Control	5	50.6 ± 16.1	2/3	–
	Healthy Control	5	54.3 ± 8.6	3/2	–
ELISA	Cryoglobulinemia	48	54.2 ± 15.7	20/28	846.59
	Healthy Control	32	48.2 ± 10.2	16/16	–

TMT-MS, tandem mass tag - labeled Mass spectrometry.

PRM-MS, Parallel reaction monitoring - Mass spectrometry.

ELISA, Enzyme-Linked Immunosorbent Assay.

### TMT-Based Quantitative Proteomics Analysis

**Figure 2:**
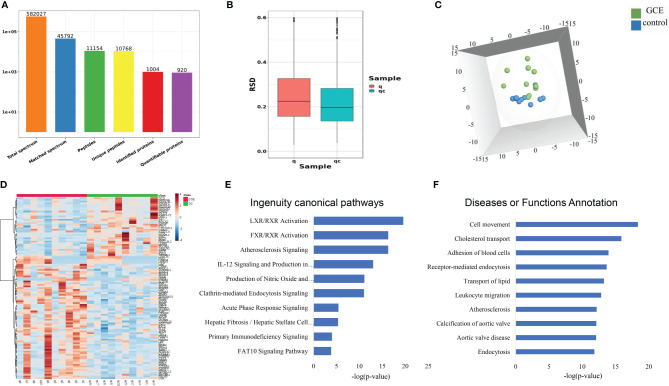
Serum proteomic profiling of CGE patients. **(A)** Protein and peptide numbers identified from the serum sample; **(B-D)** The RSD distribution, PCA analysis, and heat map analysis of samples between CGE and disease control groups; **(E, F)** IPA analysis of differentially expressed proteins between CGE patients and disease controls.

Ten CGE samples and ten DC samples were randomly selected, individually labeled with TMT reagents, and analyzed by 2D LC–MS/MS. Finally, a total of 11154 peptides and 920 proteins were identified (**Figure 2A**; **Table S2**). Principal component analysis (PCA), relative standard deviation (RSD), and Pearson’s correlation coefficient were used to evaluate the quantitative repeatability of proteins. Since three types of CGE patients were included in the CGE group, the PC of the CGE group was higher than the PC of the control group (23.4% explained var. vs. 15.5% explained var.). The RSD distribution box figure of the two groups is shown in **Figure 2B**. The median interindividual CV of the CEG group and disease control group was 22.9% and 19.5%, indicating low variation levels within the DC group. A total of 676 proteins were identified with at least 2 unique peptides, of which 667 proteins were quantified and used for further analysis.

First, to explore the proteomic profiling differences between the two groups, an unsupervised PCA was performed. The score plot showed that the CGE group could be separated from the DC group (**Figure 2C**). Second, OPLS-DA was performed to further determine the proteomic differences between the two groups. The two groups could also be separated from each other in the OPLS-DA model. One hundred permutation tests indicated that there was no overfitting in these models (**Figure S1**). Thus, a total of 109 proteins were significantly changed (fold change >1.2 and p <0.05), of which 72 proteins were upregulated and 37 proteins were downregulated when comparing the CGE and DC groups (**Figure 2D**).

The 109 CGE-related differentially expressed proteins were further analyzed by IPA. Canonical pathway analysis revealed that these proteins were significantly involved in immune/inflammatory pathways (LXR/RXR activation, FXR/RXR activation, acute phase response signaling, and primary immunodeficiency signaling) and hepatic fibrosis/hepatic stellate cell activation (**Figure 2E**; **Table S2A** **)**. In the disease and biofunction analysis, the differentially expressed proteins were mainly associated with adhesion of blood cells, leukocyte migration, cholesterol transport, and transport of lipids (**Figure 2F**; **Table S2B**).

### Validation of Differentially Expressed Proteins Using PRM-Based Proteomics

A total of 25 differentially expressed proteins in the TMT experiment were used for PRM targeted proteomics, and 22 proteins could be quantitatively identified (**Table 2**). The identification of 5 upregulated proteins, including alpha-2-macroglobulin (A2M), 72 kDa type IV collagenase (MMP2), vascular cell adhesion protein 1 (VCAM1), cell surface glycoprotein MUC18 (MCAM), and proprotein convertase subtilisin/kexin type 9 (PCSK9), and 4 downregulated proteins, including complement C3 (C3), immunoglobulin heavy constant gamma 2 (IGHG2), vitamin K-dependent protein Z (PROZ), and platelet basic protein (PPBP), was consistent with the TMT results regardless of whether the control group contained disease controls or healthy controls. Upregulated proteins are mostly related to cholesterol metabolism, whereas downregulated proteins are associated with immune regulation.

**Table 2:** Validation of biomarker candidates by PRM analysis.

Protein Accession	Protein Gene	Protein description	CGE/DC+HC (PRM)	CGE/DC (PRM)	CGE/DC(TMT)
P00739	HPR	Haptoglobin-related protein	0.79	1.01	0.49
P01023	A2M	Alpha-2-macroglobulin*	2.05	2.20	1.36
P01024	C3	**Complement C3#**	0.86	0.96	0.80
P01859	IGHG2	**Immunoglobulin heavy constant gamma 2#**	0.75	0.79	0.62
P02654	APOC1	Apolipoprotein C-I	0.84	1.17	2.07
P02655	APOC2	Apolipoprotein C-II	0.82	1.02	1.62
P02656	APOC3	Apolipoprotein C-III	1.15	0.93	1.60
P02743	APCS	Serum amyloid P-component	0.85	1.12	0.66
P08253	MMP2	72 kDa type IV collagenase*	1.22	1.35	1.46
P08519	LPA	Apolipoprotein(a)	2.00	0.99	1.35
P0DOY3	IGLC3	Immunoglobulin lambda constant 3	1.16	0.93	0.50
P18428	LBP	Lipopolysaccharide-binding protein	0.84	0.53	1.41
P19320	VCAM1	Vascular cell adhesion protein 1*	2.12	1.82	1.38
P22891	PROZ	**Vitamin K-dependent protein Z#**	0.61	0.90	0.71
P22897	MRC1	Macrophage mannose receptor 1	1.97	0.82	1.47
P43121	MCAM	Cell surface glycoprotein MUC18*	1.26	1.32	1.32
P49913	CAMP	Cathelicidin antimicrobial peptide	0.60	0.92	1.51
P55058	PLTP	Phospholipid transfer protein	0.90	0.97	1.22
Q8NBP7	PCSK9	Proprotein convertase subtilisin/kexin type 9*	1.04	1.17	1.22
P02775	PPBP	**Platelet basic protein#**	0.58	0.64	0.77
P55103	INHBC	Inhibin beta C chain	0.94	0.82	1.20
Q9NPH3	IL1RAP	Interleukin-1 receptor accessory protein	0.97	1.26	1.06

The bold font indicates that the expression ratio trend is consistent in different control groups.

*indicates that the results are all elevated. #indicates that the results are all decreased.

### Verification of Proteins in the Cholesterol Metabolism Pathway by Immunoassay

**Figure 3:**
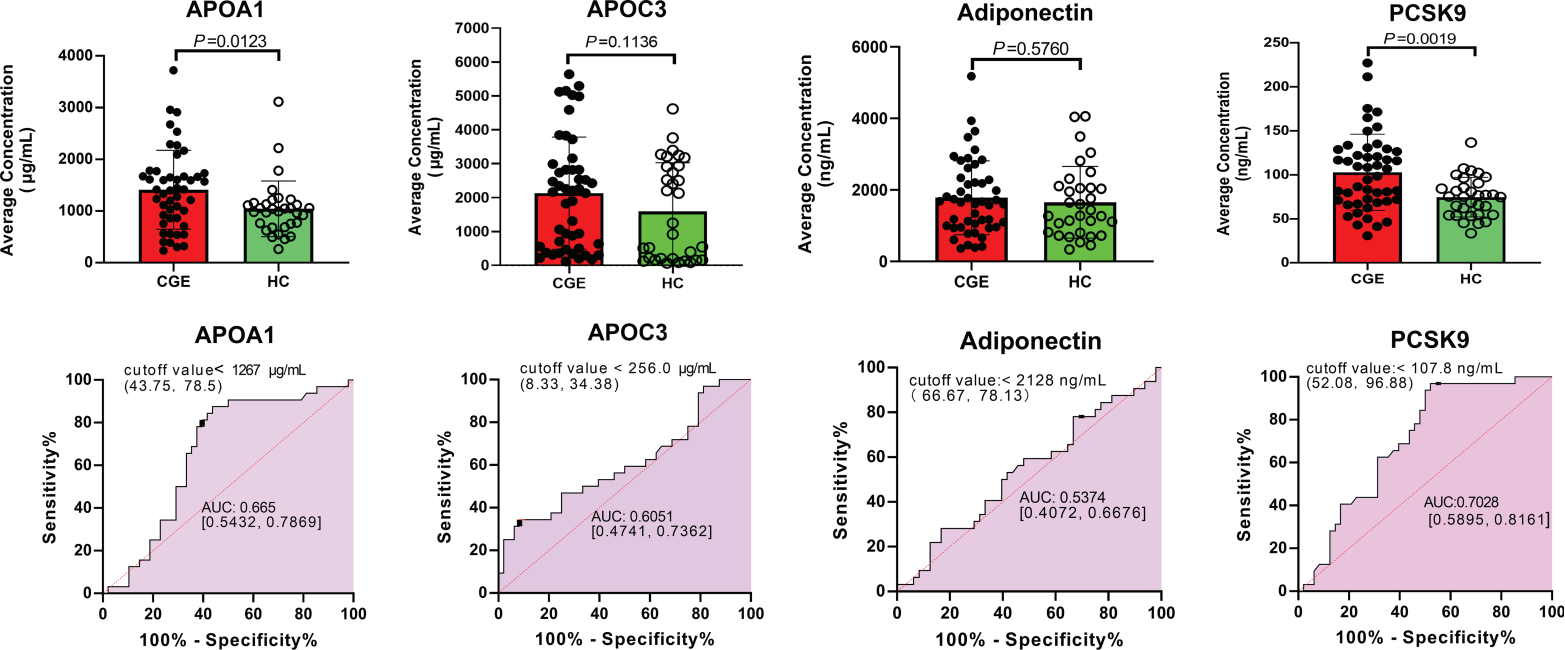
ELISA validation of APOA1, APOC3, Adiponectin and PCSK9 in another cohort. **(A)** The concentration comparison and **(B)** ROC analysis of APOA1, APOC3, Adiponectin and PCSK9 between CGE patients and healthy controls.

Given that differential proteins involved in cholesterol metabolism were enriched in the TMT experiment and validated by PRM analysis, we further verified four differential proteins in this pathway, including APOA1, APOC3, adiponectin, and PCSK9, based on an increased sample size using the ELISA method. The average concentrations of ApoA1, Apo CIII, Adiponectin, and PCSK9 in the CGE group were greater than those in the healthy control group (*P* value = 0.0123, 0.1136, 0.5760, and 0.0019, respectively). ROC curve analysis showed that the areas under the curve (AUCs) of APOA1, APOC3, adiponectin, and PCSK9 were 0.665, 0.6051, 0.5374, and 0.7028, respectively (**Figure 3**). The AUC of a combination of ApoA1 and PCSK9 reached 0.761. The concentration and ROC curve comparison of APOA1, APOC3, Adiponectin, and PCSK9 stratified by sex are shown in **Figure S2**.

## Discussion

In this study, we enrolled CGE patients, disease controls, and healthy controls to investigate the differential proteins in three groups with TMT-based proteomics, PRM-based proteomics, and immunoassays. A total of 920 serum proteins were quantitatively identified. Compared with the disease control group, 62 proteins were upregulated, and 56 proteins were downregulated in the CGE patients. These differentially expressed proteins were significantly involved in immune/inflammatory pathways, the atherosclerosis pathway, and hepatic fibrosis/hepatic stellate cell activation. In the disease and biofunction analysis, these differentially expressed proteins were mainly associated with adhesion of blood cells, leukocyte migration, cholesterol transport, and transport of lipids. Then, we selected twelve differentially expressed proteins for PRM validation in CGE patients, disease control patients, and healthy controls. Upregulated proteins are mostly related to cholesterol metabolism, while downregulated proteins are associated with immune regulation. For further verification, ELISA tests for APOA1, APOC3, Adiponectin, and PCSK9, which are involved in the pathway of cholesterol metabolism, were performed. APOA1 and PCSK9 levels were significantly increased compared with healthy controls, and ROC analysis showed that the AUC of the combination of APOA1 and PCSK9 levels reached 0.761.

The pathogenesis of MC remains unclear. B lymphocyte proliferation plays a central role in various hypotheses with viral infection and cytokines involved in the process of disease (13, 14). When B-cell homeostasis is destroyed, the B-cell clone is reactivated, leading to MC recurrence (15, 16). B lymphocyte activation and antibody production processes require metabolic regulation to meet increased energy requirements. When autoreactive B-cell receptors are overactivated, energy requirements are permanently increased (17). Germinal center B cells are critical to the generation of long-lived humoral immunity. These cells mainly derive their capacity from oxidized fatty acids, and glycolysis provides only a small amount of energy (18). Therefore, these findings could suggest that lipid metabolism plays an essential role in lymphocyte proliferation. In this study, we noted that proteins involved in cholesterol metabolism were verified in CGE patients. This is a novel discovery in CGE research.

Few studies have been conducted on cholesterol metabolism and its relationship to the pathogenesis of CGE, but its role in CGE-related diseases has been reported. Type I CGE often occurs in hematological disorders, especially lymphomas. It has been reported that some enzymes in the cholesterol biosynthesis pathway can drive lymphomagenesis in B-cell lymphomas (19). *In vitro* studies have shown that if nutrient supply is restricted, it can prevent the malignant transformation of precursor B cells (20). Type II CGE is mostly secondary to hepatitis virus infection. The impairment of lipid metabolism has been reported during the rapid progression and unfavorable therapeutic outcomes in patients with chronic hepatitis C. Direct-acting antiviral agents evolve with favoring lipoprotein/apo metabolism (21). Type III CGE is associated with autoimmune disease. Cholesterol imbalance has been implicated as a contributor to immune dysfunction. New therapies targeting cholesterol metabolism could be beneficial in the setting of autoimmune disease (22). In our study, we stratified the concentrations of APOA1, APOC3, adiponectin and PCSK9 based on different types of CGE. No significant differences were noted among the various types of CGE (**Figure S3**). Therefore, we assume that cholesterol metastasis disorder is involved in the development of CGE, regardless of the primary disease.

Cholesterol homeostasis is regulated by well-balanced mechanisms of intestinal uptake, endogenous synthesis, transport in lipoprotein particles, and biliary excretion. APOA1 and APOC3 are components of lipoproteins, mainly high-density lipoproteins and chylomicrons. These proteins play an important role in the transportation of lipoprotein particles. Adiponectin is a protein produced and secreted by adipocytes. Adiponectin concentrations in plasma are generally inversely proportional to fat mass. PCSK9, the ninth member of the proprotein invertase family, binds to the low-density lipoprotein receptor (LDLR) on the cell surface to form a complex, which prevents the formation of LDLR extracellular region rearrangement and hairpin structure, making it unable to recycle to the cell surface (23). PCSK9 and APOC3 inhibitors have been successfully tested as treatments for atherosclerosis (24). The typical clinical manifestations of CGE, especially mixed CGE, including type II and type III, are vasculitis. It has been reported that lipid levels, including total cholesterol, low-density lipoprotein, and apolipoprotein B, increased during remission induction among patients with anti-neutrophil cytoplasmic antibody-associated vasculitis (25). Lipid and lipoprotein profiles were compared in active or remission systemic vasculitis patients and healthy subjects. The concentrations of total cholesterol, high-density lipoprotein, APOA1 and apolipoprotein B increased sequentially in healthy subjects, active patients, and remission patients (26). Circulating adiponectin levels are decreased in the acute stage of Kawasaki disease, an acute febrile illness characterized by systemic vasculitis (27). Given that the adiponectin levels of the CGE groups in this study were greater than those of the healthy controls and not consistent with a previous study, we assumed that the treatment and disease status of CGE patients affected the results. PCSK9 levels in the peripheral blood of patients with SLE are significantly increased, which is associated with disease activity and poor prognosis (28). Further studies on PCSK9 showed that it played an important role in vasculitis (29, 30). Antiphospholipid syndrome is mainly characterized by thrombosis in small and medium blood vessels, and thrombi can form in both arteries and veins. Studies have shown that high titers of antiphospholipid antibodies in antiphospholipid syndrome are associated with single nucleotide mutations in LDLR and PCSK9 (31). Our findings were consistent with the conclusions of previous studies. Further research should be performed on more patients or in different animal models.

This report describes the first application of a TMT-PRM-ELISA workflow to identify and validate CGE-specific biomarkers in serum. The limitation of our study is the lack of patient follow-up, which would facilitate dynamic analysis of the biomarkers. However, the sample size was larger than that noted in previous studies, and our study is the first proteomic study of CGE in China. Therefore, the findings reported here are instructive for CGE pathogenesis research and biomarker screening.

## Conclusions and Future Perspectives

Using a proteomic method, APOA1 and PCSK9 levels are increased in CGE patients, demonstrating that proteins with important roles in cholesterol metabolism are involved in the development of CGE. These findings contribute to CGE pathogenesis research and biomarker discovery. Moreover, drugs targeting APOA1 and PCSK9 may represent novel therapies for CGE treatment.

## Data Availability Statement

The original contributions presented in the study have been uploaded to the ProteomeXchange Consortium *via* the iProX partner repository. This data can be found here: https://www.iprox.cn/page/PDV0141.html.

## Ethics Statement

The studies involving human participants were reviewed and approved by the ethics committee of Peking Union Medical College Hospital (S-K904). The ethics committee waived the requirement of written informed consent for participation.

## Author Contributions

PL proposed this study. JW helped the proteomic tests. WS conducted the electrophoresis tests and contacted with clinicians. DS collected the samples. JW and XT analyzed the data. ZQ and YL gave the comments for revision. XQ wrote the draft. All authors have accepted responsibility for the entire content of this manuscript and approved submission.

## Funding

This work was supported by grants from CAMS Innovation Fund for Medical Sciences (CIFMS 2021-I2M-1-003) and Basic Research Funds of Central Public Research Institutes, Chinese Academy of Medical Sciences (2020-RW330-004).

## Conflict of Interest

The authors declare that the research was conducted in the absence of any commercial or financial relationships that could be construed as a potential conflict of interest.

## Publisher’s Note

All claims expressed in this article are solely those of the authors and do not necessarily represent those of their affiliated organizations, or those of the publisher, the editors and the reviewers. Any product that may be evaluated in this article, or claim that may be made by its manufacturer, is not guaranteed or endorsed by the publisher.
