# Supplementary material for: Proteomic Profiling of Cryoglobulinemia
Source: Front Immunol. 2022 May 23;13:855513. doi: 10.3389/fimmu.2022.855513 (PMC9167934; doi:10.3389/fimmu.2022.855513)
Supplement: Supplementary file 1 [file DataSheet_1.docx]

**Table S1. Clinical characteristics of enrolled subjects**

| Analysis | Group | N | Cryoglobulin typing | Age | Gender | Diagnosis | CG quantity（g/L） | Monoclonal analysis | HCV Ab | HBs Ag |
| --- | --- | --- | --- | --- | --- | --- | --- | --- | --- | --- |
| TMT-MS | Cryoglobulinemia | 1 | I | 66 | F | Multiple myeloma | 2476.3 | IgGλ | - | - |
|  |  | 2 | I | 69 | M | CGE | 3974.8 | IgGλ | - | - |
|  |  | 3 | I | 74 | M | CGE, Systemic vasculitis | 150 | IgMκ | - | - |
|  |  | 4 | II | 55 | F | CGE, macroglobulinemia, cold agglutinin disease | 10054 | IgMκ | - | + |
|  |  | 5 | II | 54 | F | CGE, Connective tissue disease; Facial paralysis | 110.7 | IgMκ | - | - |
|  |  | 6 | II | 65 | F | CGE, Urinary tract infection | 1223.4 | IgMκ | - | - |
|  |  | 7 | II | 48 | F | CGE | 1071.7 | IgAλ | - | + |
|  |  | 8 | III | 52 | M | CGE | 32 | NA | - | - |
|  |  | 9 | III | 65 | M | CGE | 40 | NA | - | - |
|  |  | 10 | III | 45 | M | CGE | 64.3 | NA | - | + |
|  | Disease Control | 1 | - | 67 | M | Lymphoplasmacytic lymphoma | - | - | - | - |
|  |  | 2 | - | 55 | M | Purpura | - | - | - | - |
|  |  | 3 | - | 49 | F | sjogren's syndrome | - | - | - | - |
|  |  | 4 | - | 51 | M | Chronic glomerulonephritis | - | - | - | - |
|  |  | 5 | - | 60 | F | Cold Agglutinin Disease | - | - | - | - |
|  |  | 6 | - | 46 | F | Leukocytoclastic vasculitis | - | - | - | - |
|  |  | 7 | - | 62 | F | Allergic purpura nephritis | - | - | - | - |
|  |  | 8 | - | 65 | F | Giant cell arteritis | - | - | - | + |
|  |  | 9 | - | 53 | M | glomerulonephritis | - | - | - | + |
|  |  | 10 | - | 61 | F | Leukopenia | - | - | - | - |
| PRM-MS | Cryoglobulinemia | 1 | I | 60 | M | CGE, Macroglobulinemia | 125.8 | IgMκ | - | - |
|  |  | 2 | I | 39 | F | CGE | 629.9 | IgGκ | - | - |
|  |  | 3 | I | 52 | M | CGE | 305 | IgGλ | - | - |
|  |  | 4 | II | 29 | F | CGE | 107.1 | IgMκ | - | - |
|  |  | 5 | II | 43 | F | CGE, Indolent lymphoma | 149.2 | IgMκ | - | + |
|  |  | 6 | II | 47 | F | CGE | 1997.0 | IgAλ | - | - |
|  |  | 7 | II | 54 | F | CGE, Chronic hepatitis B | 25.1 | IgMκ | - | + |
|  |  | 8 | III | 55 | M | CGE, Necrotizing vasculitis; Virus infection | 15.3 | - | - | - |
|  |  | 9 | III | 65 | M | CGE, Chronic glomerulonephritis, Anemia | 511.4 | - | - | - |
|  |  | 10 | III | 51 | M | CGE, Proteinuria, Haematuria | 14.8 | - | - | - |
|  | Disease Control | 1 | - | 49 | F | Allergic purpura nephritis | - | - | - | - |
|  |  | 2 | - | 60 | M | Acute kidney failure | - | - | - | - |
|  |  | 3 | - | 23 | F | Purpura | - | - | - | - |
|  |  | 4 | - | 54 | M | Polyarteritis nodosa | - | - | - | - |
|  |  | 5 | - | 64 | F | Allergic purpura nephritis | - | - | - | - |
|  | Healthy Control | 5 | - | 54.3±8.6 | 3/2 | - | - | - |  |  |
| ELISA | Cryoglobulinemia | 1 | I | 69 | M | CGE | 2551.5 | IgGκ | - | - |
|  |  | 2 |  |  |  |  |  |  |  |  |
|  |  | 3 | I | 77 | F | Cold Agglutinin Disease | 44.6 | IgMκ | - | - |
|  |  | 4 | I | 73 | F | Non-hodgkin's lymphoma | 24.3 | IgMκ | - | - |
|  |  | 5 | I | 55 | M | Acquired Immune Deficiency Syndrome | 55.5 | IgGλ | - | - |
|  |  | 6 | I | 56 | F | Non-hodgkin's lymphoma | 46.7 | IgMλ | - | - |
|  |  | 7 | I | 70 | F | Macroglobulinemia | 520.1 | IgMκ | - | - |
|  |  | 8 | I | 56 | M | Cold Agglutinin Disease | 19.6 | IgMκ | NA | NA |
|  |  | 9 | I | 66 | F | CGE | 1569.6 | IgMλ | - | - |
|  |  | 10 | I | 74 | F | CGE | 4991.3 | IgGκ | NA | NA |
|  |  | 11 | II | 78 | F | CGE, Diabetes Mellitus, Hypertension, Chronic renal insufficiency | 31.5 | IgMλ | - | - |
|  |  | 12 | II | 36 | F | CGE, Chronic hepatitis B, Glomerulonephritis | 208.5 | IgMκ | - | + |
|  |  | 13 | II | 43 | F | CGE, Non-hodgkin's lymphoma | 575.1 | IgMκ | - | + |
|  |  | 14 | II | 68 | F | CGE, Chronic renal insufficiency, Diabetes Mellitus | 2926.6 | IgMκ | - | - |
|  |  | 15 | II | 57 | F | CGE, Diabetes Mellitus | 4345.1 | IgMκ | - | + |
|  |  | 16 | II | 47 | F | CGE | 792.2 | IgMκ | - | - |
|  |  | 17 | II | 64 | M | Chronic lymphocytic leukemia, Hypertension | 27 | IgMκ | - | - |
|  |  | 18 | II | 67 | M | Chronic glomerulonephritis, CGE | 30.1 | IgMλ | - | - |
|  |  | 19 | II | 15 | M | CGE | 686.8 | IgMλ | - | + |
|  |  | 20 | II | 16 | M | CGE, Gout, Hypertension | 17075.8 | IgMκ | + | - |
|  |  | 21 | III | 15 | M | Fever of Unknown Origin | 22.5 | - | - | - |
|  |  | 22 | III | 56 | M | Purpura, urticaria, hyperglobulinemia | 32.5 | - | NA | NA |
|  |  | 23 | III | 68 | F | Peripheral neuropathy | 46.7 | - | - | - |
|  |  | 24 | III | 21 | F | Systemic lupus erythematosus | 88.3 | - | - | - |
|  |  | 25 | III | 54 | F | Renal insufficiency, Leukopenia, diabetes mellitus, hypertension, hyperthyroidism | 25.3 | - | + | - |
|  |  | 26 | III | 59 | F | IgG4 related diseases | 0 | - | - | - |
|  |  | 27 | III | 63 | M | Peripheral T cell lymphoma | 166 | - | - | - |
|  |  | 28 | III | 36 | M | Antiphospholipid antibody syndrome | 25.8 | - | - | - |
|  |  | 29 | III | 58 | F | CGE, Chronic hepatitis B, Chronic renal insufficiency | 45.4 | - | - | + |
|  |  | 30 | III | 37 | F | Acute kidney injury | 33.6 | - | - | - |
|  |  | 31 | III | 81 | M | Thrombocytopenia | 27.7 | - | - | - |
|  |  | 32 | III | 40 | F | Systemic lupus erythematosus | 40.5 | - | - | - |
|  |  | 33 | III | 48 | F | Rheumatoid arthritis | 44.8 | - | - | - |
|  |  | 34 | III | 72 | M | Renal failure | 23.4 | - | - | - |
|  |  | 35 | III | 43 | M | Rheumatoid arthritis | 46.9 | - | - | - |
|  |  | 36 | III | 31 | M | Nephrotic syndrome | 116.4 | - | - | - |
|  |  | 37 | III | 65 | F | Fever of Unknown Origin | 19.6 | - | - | - |
|  |  | 38 | III | 56 | F | Lung infection, Rash | 58.7 | - | - | - |
|  |  | 39 | III | 67 | M | ANCA-related vasculitis | 61.1 | - | - | - |
|  |  | 40 | III | 46 | M | Renal insufficiency | 41.6 | - | - | - |
|  |  | 41 | III | 44 | F | Fanconi syndrome | 56.7 | - | - | - |
|  |  | 42 | III | 56 | M | Anemia | 257.2 | - | - | - |
|  |  | 43 | III | 54 | M | Chronic renal insufficiency | 50 | - | - | - |
|  |  | 44 | III | 40 | F | Systemic lupus erythematosus | 24.5 | - | - | - |
|  |  | 45 | III | 43 | F | IgA nephropathy | 100.6 | - | - | - |
|  |  | 46 | III | 62 | M | Chronic renal insufficiency | 45.3 | - | - | - |
|  |  | 47 | III | 22 | F | Systemic lupus erythematosus | 84.6 | - | NA | NA |
|  |  | 48 | III | 65 | F | Acute kidney injury | 52.7 | - | - | - |
|  | Healthy Control | 32 | - | 48.2±10.2 | 16/16 | - | - | - | - | - |

**Table S2. Identified proteins by TMT-based quantitative proteomics analysis**

| **Protein accession** | **Gene name** | **Protein description** | **Ratio** |
| --- | --- | --- | --- |
| P02656 | APOC3 | Apolipoprotein C-III OS=Homo sapiens OX=9606 GN=APOC3 PE=1 SV=1 | 1.598 |
| P05062 | ALDOB | Fructose-bisphosphate aldolase B OS=Homo sapiens OX=9606 GN=ALDOB PE=1 SV=2 | 1.497 |
| P05155 | SERPING1 | Plasma protease C1 inhibitor OS=Homo sapiens OX=9606 GN=SERPING1 PE=1 SV=2 | 1.701 |
| P0DOX5 | --- | Immunoglobulin gamma-1 heavy chain OS=Homo sapiens OX=9606 PE=1 SV=2 | 0.429 |
| Q9NR12 | PDLIM7 | PDZ and LIM domain protein 7 OS=Homo sapiens OX=9606 GN=PDLIM7 PE=1 SV=1 | 0.369 |
| P02647 | APOA1 | Apolipoprotein A-I OS=Homo sapiens OX=9606 GN=APOA1 PE=1 SV=1 | 1.579 |
| Q9H156 | SLITRK2 | SLIT and NTRK-like protein 2 OS=Homo sapiens OX=9606 GN=SLITRK2 PE=1 SV=1 | 0.564 |
| P01023 | A2M | Alpha-2-macroglobulin OS=Homo sapiens OX=9606 GN=A2M PE=1 SV=3 | 1.359 |
| P49257 | LMAN1 | Protein ERGIC-53 OS=Homo sapiens OX=9606 GN=LMAN1 PE=1 SV=2 | 1.634 |
| A0A0C4DH25 | IGKV3D-20 | Immunoglobulin kappa variable 3D-20 OS=Homo sapiens OX=9606 GN=IGKV3D-20 PE=3 SV=1 | 0.561 |
| Q0VF96 | CGNL1 | Cingulin-like protein 1 OS=Homo sapiens OX=9606 GN=CGNL1 PE=1 SV=2 | 0.475 |
| Q9UIB8 | CD84 | SLAM family member 5 OS=Homo sapiens OX=9606 GN=CD84 PE=1 SV=1 | 0.668 |
| Q86UD1 | OAF | Out at first protein homolog OS=Homo sapiens OX=9606 GN=OAF PE=2 SV=1 | 1.412 |
| A0A0J9YX35 | IGHV3-64D | Immunoglobulin heavy variable 3-64D OS=Homo sapiens OX=9606 GN=IGHV3-64D PE=3 SV=1 | 0.295 |
| Q15848 | ADIPOQ | Adiponectin OS=Homo sapiens OX=9606 GN=ADIPOQ PE=1 SV=1 | 1.742 |
| P36222 | CHI3L1 | Chitinase-3-like protein 1 OS=Homo sapiens OX=9606 GN=CHI3L1 PE=1 SV=2 | 2.458 |
| Q7L2Z9 | CENPQ | Centromere protein Q OS=Homo sapiens OX=9606 GN=CENPQ PE=1 SV=1 | 1.604 |
| P0DOX8 | --- | Immunoglobulin lambda-1 light chain OS=Homo sapiens OX=9606 PE=1 SV=1 | 0.66 |
| Q9UPY3 | DICER1 | Endoribonuclease Dicer OS=Homo sapiens OX=9606 GN=DICER1 PE=1 SV=3 | 0.699 |
| P06727 | APOA4 | Apolipoprotein A-IV OS=Homo sapiens OX=9606 GN=APOA4 PE=1 SV=3 | 1.503 |
| P33241 | LSP1 | Lymphocyte-specific protein 1 OS=Homo sapiens OX=9606 GN=LSP1 PE=1 SV=1 | 0.442 |
| Q9Y657 | SPIN1 | Spindlin-1 OS=Homo sapiens OX=9606 GN=SPIN1 PE=1 SV=3 | 0.585 |
| O00151 | PDLIM1 | PDZ and LIM domain protein 1 OS=Homo sapiens OX=9606 GN=PDLIM1 PE=1 SV=4 | 0.637 |
| A0A0C4DH34 | IGHV4-28 | Immunoglobulin heavy variable 4-28 OS=Homo sapiens OX=9606 GN=IGHV4-28 PE=3 SV=1 | 0.241 |
| Q5VUA4 | ZNF318 | Zinc finger protein 318 OS=Homo sapiens OX=9606 GN=ZNF318 PE=1 SV=2 | 1.482 |
| P07998 | RNASE1 | Ribonuclease pancreatic OS=Homo sapiens OX=9606 GN=RNASE1 PE=1 SV=4 | 1.67 |
| P25786 | PSMA1 | Proteasome subunit alpha type-1 OS=Homo sapiens OX=9606 GN=PSMA1 PE=1 SV=1 | 1.352 |
| P13611 | VCAN | Versican core protein OS=Homo sapiens OX=9606 GN=VCAN PE=1 SV=3 | 1.386 |
| P16035 | TIMP2 | Metalloproteinase inhibitor 2 OS=Homo sapiens OX=9606 GN=TIMP2 PE=1 SV=2 | 1.397 |
| P0DOX7 | --- | Immunoglobulin kappa light chain OS=Homo sapiens OX=9606 PE=1 SV=1 | 0.681 |
| Q5T890 | ERCC6L2 | DNA excision repair protein ERCC-6-like 2 OS=Homo sapiens OX=9606 GN=ERCC6L2 PE=1 SV=2 | 0.702 |
| P16109 | SELP | P-selectin OS=Homo sapiens OX=9606 GN=SELP PE=1 SV=3 | 0.746 |
| P06312 | IGKV4-1 | Immunoglobulin kappa variable 4-1 OS=Homo sapiens OX=9606 GN=IGKV4-1 PE=1 SV=1 | 0.496 |
| P04211 | IGLV7-43 | Immunoglobulin lambda variable 7-43 OS=Homo sapiens OX=9606 GN=IGLV7-43 PE=3 SV=2 | 0.587 |
| P08514 | ITGA2B | Integrin alpha-IIb OS=Homo sapiens OX=9606 GN=ITGA2B PE=1 SV=3 | 0.599 |
| P49913 | CAMP | Cathelicidin antimicrobial peptide OS=Homo sapiens OX=9606 GN=CAMP PE=1 SV=1 | 1.514 |
| P02743 | APCS | Serum amyloid P-component OS=Homo sapiens OX=9606 GN=APCS PE=1 SV=2 | 0.656 |
| Q3ZCW2 | LGALSL | Galectin-related protein OS=Homo sapiens OX=9606 GN=LGALSL PE=1 SV=2 | 0.679 |
| Q96JG8 | MAGED4 | Melanoma-associated antigen D4 OS=Homo sapiens OX=9606 GN=MAGED4 PE=1 SV=3 | 0.71 |
| P37802 | TAGLN2 | Transgelin-2 OS=Homo sapiens OX=9606 GN=TAGLN2 PE=1 SV=3 | 0.548 |
| P02654 | APOC1 | Apolipoprotein C-I OS=Homo sapiens OX=9606 GN=APOC1 PE=1 SV=1 | 2.074 |
| O43866 | CD5L | CD5 antigen-like OS=Homo sapiens OX=9606 GN=CD5L PE=1 SV=1 | 0.31 |
| Q16851 | UGP2 | UTP--glucose-1-phosphate uridylyltransferase OS=Homo sapiens OX=9606 GN=UGP2 PE=1 SV=5 | 1.72 |
| P02655 | APOC2 | Apolipoprotein C-II OS=Homo sapiens OX=9606 GN=APOC2 PE=1 SV=1 | 1.615 |
| P22891 | PROZ | Vitamin K-dependent protein Z OS=Homo sapiens OX=9606 GN=PROZ PE=1 SV=2 | 0.707 |
| Q9UMZ3 | PTPRQ | Phosphatidylinositol phosphatase PTPRQ OS=Homo sapiens OX=9606 GN=PTPRQ PE=1 SV=2 | 0.365 |
| P18428 | LBP | Lipopolysaccharide-binding protein OS=Homo sapiens OX=9606 GN=LBP PE=1 SV=3 | 1.411 |
| P18206 | VCL | Vinculin OS=Homo sapiens OX=9606 GN=VCL PE=1 SV=4 | 1.384 |
| Q8IVL5 | P3H2 | Prolyl 3-hydroxylase 2 OS=Homo sapiens OX=9606 GN=P3H2 PE=1 SV=1 | 0.505 |
| Q9NQ38 | SPINK5 | Serine protease inhibitor Kazal-type 5 OS=Homo sapiens OX=9606 GN=SPINK5 PE=1 SV=2 | 1.384 |
| P08519 | LPA | Apolipoprotein(a) OS=Homo sapiens OX=9606 GN=LPA PE=1 SV=1 | 1.347 |
| P13796 | LCP1 | Plastin-2 OS=Homo sapiens OX=9606 GN=LCP1 PE=1 SV=6 | 1.335 |
| A0A0C4DH68 | IGKV2-24 | Immunoglobulin kappa variable 2-24 OS=Homo sapiens OX=9606 GN=IGKV2-24 PE=3 SV=1 | 0.484 |
| A0A0B4J1V6 | IGHV3-73 | Immunoglobulin heavy variable 3-73 OS=Homo sapiens OX=9606 GN=IGHV3-73 PE=3 SV=1 | 0.468 |
| P08253 | MMP2 | 72 kDa type IV collagenase OS=Homo sapiens OX=9606 GN=MMP2 PE=1 SV=2 | 1.457 |
| P07195 | LDHB | L-lactate dehydrogenase B chain OS=Homo sapiens OX=9606 GN=LDHB PE=1 SV=2 | 1.395 |
| P0DOY3 | IGLC3 | Immunoglobulin lambda constant 3 OS=Homo sapiens OX=9606 GN=IGLC3 PE=1 SV=1 | 0.497 |
| Q14766 | LTBP1 | Latent-transforming growth factor beta-binding protein 1 OS=Homo sapiens OX=9606 GN=LTBP1 PE=1 SV=4 | 0.722 |
| P15924 | DSP | Desmoplakin OS=Homo sapiens OX=9606 GN=DSP PE=1 SV=3 | 0.71 |
| P04114 | APOB | Apolipoprotein B-100 OS=Homo sapiens OX=9606 GN=APOB PE=1 SV=2 | 1.431 |
| P00739 | HPR | Haptoglobin-related protein OS=Homo sapiens OX=9606 GN=HPR PE=2 SV=2 | 0.49 |
| Q6EEV6 | SUMO4 | Small ubiquitin-related modifier 4 OS=Homo sapiens OX=9606 GN=SUMO4 PE=1 SV=2 | 0.648 |
| Q6UVK1 | CSPG4 | Chondroitin sulfate proteoglycan 4 OS=Homo sapiens OX=9606 GN=CSPG4 PE=1 SV=2 | 1.534 |
| P01859 | IGHG2 | Immunoglobulin heavy constant gamma 2 OS=Homo sapiens OX=9606 GN=IGHG2 PE=1 SV=2 | 0.622 |
| O14773 | TPP1 | Tripeptidyl-peptidase 1 OS=Homo sapiens OX=9606 GN=TPP1 PE=1 SV=2 | 1.381 |
| Q9BXJ4 | C1QTNF3 | Complement C1q tumor necrosis factor-related protein 3 OS=Homo sapiens OX=9606 GN=C1QTNF3 PE=1 SV=1 | 0.669 |
| Q15404 | RSU1 | Ras suppressor protein 1 OS=Homo sapiens OX=9606 GN=RSU1 PE=1 SV=3 | 0.749 |
| Q6PEV8 | FAM199X | Protein FAM199X OS=Homo sapiens OX=9606 GN=FAM199X PE=1 SV=1 | 0.521 |
| Q8NF91 | SYNE1 | Nesprin-1 OS=Homo sapiens OX=9606 GN=SYNE1 PE=1 SV=4 | 1.317 |
| P24592 | IGFBP6 | Insulin-like growth factor-binding protein 6 OS=Homo sapiens OX=9606 GN=IGFBP6 PE=1 SV=1 | 1.451 |
| Q8NFR7 | CCDC148 | Coiled-coil domain-containing protein 148 OS=Homo sapiens OX=9606 GN=CCDC148 PE=1 SV=2 | 1.905 |
| Q6AI12 | ANKRD40 | Ankyrin repeat domain-containing protein 40 OS=Homo sapiens OX=9606 GN=ANKRD40 PE=1 SV=2 | 2.757 |
| P02649 | APOE | Apolipoprotein E OS=Homo sapiens OX=9606 GN=APOE PE=1 SV=1 | 1.41 |
| P01034 | CST3 | Cystatin-C OS=Homo sapiens OX=9606 GN=CST3 PE=1 SV=1 | 1.448 |
| Q4LDE5 | SVEP1 | Sushi, von Willebrand factor type A, EGF and pentraxin domain-containing protein 1 OS=Homo sapiens OX=9606 GN=SVEP1 PE=1 SV=3 | 1.788 |
| P25788 | PSMA3 | Proteasome subunit alpha type-3 OS=Homo sapiens OX=9606 GN=PSMA3 PE=1 SV=2 | 1.624 |
| P01780 | IGHV3-7 | Immunoglobulin heavy variable 3-7 OS=Homo sapiens OX=9606 GN=IGHV3-7 PE=1 SV=2 | 0.504 |
| P01709 | IGLV2-8 | Immunoglobulin lambda variable 2-8 OS=Homo sapiens OX=9606 GN=IGLV2-8 PE=1 SV=2 | 0.497 |
| P0DJI8 | SAA1 | Serum amyloid A-1 protein OS=Homo sapiens OX=9606 GN=SAA1 PE=1 SV=1 | 2.278 |
| Q9NZK5 | ADA2 | Adenosine deaminase 2 OS=Homo sapiens OX=9606 GN=ADA2 PE=1 SV=2 | 1.43 |
| O94885 | SASH1 | SAM and SH3 domain-containing protein 1 OS=Homo sapiens OX=9606 GN=SASH1 PE=1 SV=3 | 2.55 |
| A0A075B6K4 | IGLV3-10 | Immunoglobulin lambda variable 3-10 OS=Homo sapiens OX=9606 GN=IGLV3-10 PE=3 SV=2 | 0.577 |
| A2NJV5 | IGKV2-29 | Immunoglobulin kappa variable 2-29 OS=Homo sapiens OX=9606 GN=IGKV2-29 PE=3 SV=2 | 0.518 |
| A0A0B4J1U7 | IGHV6-1 | Immunoglobulin heavy variable 6-1 OS=Homo sapiens OX=9606 GN=IGHV6-1 PE=3 SV=1 | 0.346 |
| P01834 | IGKC | Immunoglobulin kappa constant OS=Homo sapiens OX=9606 GN=IGKC PE=1 SV=2 | 0.388 |
| O15335 | CHAD | Chondroadherin OS=Homo sapiens OX=9606 GN=CHAD PE=1 SV=2 | 0.726 |
| P05090 | APOD | Apolipoprotein D OS=Homo sapiens OX=9606 GN=APOD PE=1 SV=1 | 2.096 |
| Q9P1Z9 | CCDC180 | Coiled-coil domain-containing protein 180 OS=Homo sapiens OX=9606 GN=CCDC180 PE=2 SV=3 | 4.484 |
| O95445 | APOM | Apolipoprotein M OS=Homo sapiens OX=9606 GN=APOM PE=1 SV=2 | 1.524 |
| P19320 | VCAM1 | Vascular cell adhesion protein 1 OS=Homo sapiens OX=9606 GN=VCAM1 PE=1 SV=1 | 1.376 |
| P01701 | IGLV1-51 | Immunoglobulin lambda variable 1-51 OS=Homo sapiens OX=9606 GN=IGLV1-51 PE=1 SV=2 | 0.66 |
| Q5VYJ5 | MALRD1 | MAM and LDL-receptor class A domain-containing protein 1 OS=Homo sapiens OX=9606 GN=MALRD1 PE=1 SV=4 | 1.889 |
| Q04826 | HLA-B | HLA class I histocompatibility antigen, B-40 alpha chain OS=Homo sapiens OX=9606 GN=HLA-B PE=1 SV=1 | 0.353 |
| P27169 | PON1 | Serum paraoxonase/arylesterase 1 OS=Homo sapiens OX=9606 GN=PON1 PE=1 SV=3 | 1.351 |
| P60900 | PSMA6 | Proteasome subunit alpha type-6 OS=Homo sapiens OX=9606 GN=PSMA6 PE=1 SV=1 | 1.415 |
| Q14623 | IHH | Indian hedgehog protein OS=Homo sapiens OX=9606 GN=IHH PE=1 SV=4 | 1.515 |
| P81605 | DCD | Dermcidin OS=Homo sapiens OX=9606 GN=DCD PE=1 SV=2 | 0.577 |
| P17900 | GM2A | Ganglioside GM2 activator OS=Homo sapiens OX=9606 GN=GM2A PE=1 SV=4 | 1.426 |
| P00338 | LDHA | L-lactate dehydrogenase A chain OS=Homo sapiens OX=9606 GN=LDHA PE=1 SV=2 | 1.421 |
| O43639 | NCK2 | Cytoplasmic protein NCK2 OS=Homo sapiens OX=9606 GN=NCK2 PE=1 SV=2 | 0.617 |
| P23284 | PPIB | Peptidyl-prolyl cis-trans isomerase B OS=Homo sapiens OX=9606 GN=PPIB PE=1 SV=2 | 0.702 |
| P43121 | MCAM | Cell surface glycoprotein MUC18 OS=Homo sapiens OX=9606 GN=MCAM PE=1 SV=2 | 1.318 |
| P0DP03 | IGHV3-30-5 | Immunoglobulin heavy variable 3-30-5 OS=Homo sapiens OX=9606 GN=IGHV3-30-5 PE=3 SV=1 | 0.446 |
| Q08830 | FGL1 | Fibrinogen-like protein 1 OS=Homo sapiens OX=9606 GN=FGL1 PE=1 SV=3 | 0.681 |
| A0M8Q6 | IGLC7 | Immunoglobulin lambda constant 7 OS=Homo sapiens OX=9606 GN=IGLC7 PE=1 SV=3 | 0.706 |
| Q86YW5 | TREML1 | Trem-like transcript 1 protein OS=Homo sapiens OX=9606 GN=TREML1 PE=1 SV=2 | 0.718 |
| A0A0B4J1Y9 | IGHV3-72 | Immunoglobulin heavy variable 3-72 OS=Homo sapiens OX=9606 GN=IGHV3-72 PE=3 SV=1 | 0.569 |
| P22897 | MRC1 | Macrophage mannose receptor 1 OS=Homo sapiens OX=9606 GN=MRC1 PE=1 SV=1 | 1.465 |
| P00390 | GSR | Glutathione reductase, mitochondrial OS=Homo sapiens OX=9606 GN=GSR PE=1 SV=2 | 1.334 |
| P01861 | IGHG4 | Immunoglobulin heavy constant gamma 4 OS=Homo sapiens OX=9606 GN=IGHG4 PE=1 SV=1 | 0.436 |
| P35542 | SAA4 | Serum amyloid A-4 protein OS=Homo sapiens OX=9606 GN=SAA4 PE=1 SV=2 | 1.642 |
| Q96P20 | NLRP3 | NACHT, LRR and PYD domains-containing protein 3 OS=Homo sapiens OX=9606 GN=NLRP3 PE=1 SV=3 | 1.401 |
| O75339 | CILP | Cartilage intermediate layer protein 1 OS=Homo sapiens OX=9606 GN=CILP PE=1 SV=4 | 1.765 |
| Q9Y279 | VSIG4 | V-set and immunoglobulin domain-containing protein 4 OS=Homo sapiens OX=9606 GN=VSIG4 PE=1 SV=1 | 2.083 |
| P12110 | COL6A2 | Collagen alpha-2(VI) chain OS=Homo sapiens OX=9606 GN=COL6A2 PE=1 SV=4 | 1.621 |
| Q02487 | DSC2 | Desmocollin-2 OS=Homo sapiens OX=9606 GN=DSC2 PE=1 SV=1 | 1.411 |
| P55056 | APOC4 | Apolipoprotein C-IV OS=Homo sapiens OX=9606 GN=APOC4 PE=1 SV=1 | 1.411 |
| Q99436 | PSMB7 | Proteasome subunit beta type-7 OS=Homo sapiens OX=9606 GN=PSMB7 PE=1 SV=1 | 1.422 |

**Table S2A. Canonical pathway analysis**

| Ingenuity Canonical Pathways | -log(p-value) | Ratio | z-score | Molecules |
| --- | --- | --- | --- | --- |
| LXR/RXR Activation | 19.6 | 0.138 | 2.668 | APOA1,APOA4,APOB,APOC1,APOC2,APOC3,APOC4,APOD,APOE,APOM,C3,LBP,LPA,PLTP,PON1,SAA1,SAA4 |
| FXR/RXR Activation | 16.3 | 0.12 | - | APOA1,APOA4,APOB,APOC1,APOC2,APOC3,APOC4,APOD,APOE,APOM,C3,PLTP,PON1,SAA1,SAA4 |
| Atherosclerosis Signaling | 16.3 | 0.119 | - | APOA1,APOA4,APOB,APOC1,APOC2,APOC3,APOC4,APOD,APOE,APOM,LPA,PON1,SAA4,SELP,VCAM1 |
| Maturity Onset Diabetes of Young (MODY) Signaling | 14.5 | 0.156 | - | ADIPOQ,ALDOB,APOA1,APOA4,APOB,APOC1,APOC2,APOC3,APOC4,APOD,APOE,APOM |
| IL-12 Signaling and Production in Macrophages | 13 | 0.097 | - | APOA1,APOA4,APOB,APOC1,APOC2,APOC3,APOC4,APOD,APOE,APOM,LPA,PON1,SAA4 |
| Production of Nitric Oxide and Reactive Oxygen Species in Macrophages | 11.1 | 0.0691 | 3.606 | APOA1,APOA4,APOB,APOC1,APOC2,APOC3,APOC4,APOD,APOE,APOM,LPA,PON1,SAA4 |
| Clathrin-mediated Endocytosis Signaling | 11 | 0.0677 | - | APOA1,APOA4,APOB,APOC1,APOC2,APOC3,APOC4,APOD,APOE,APOM,LPA,PON1,SAA4 |
| Acute Phase Response Signaling | 5.45 | 0.0435 | 0 | A2M,APCS,APOA1,C3,LBP,SAA1,SAA4,SERPING1 |
| Hepatic Fibrosis / Hepatic Stellate Cell Activation | 5.35 | 0.0421 | - | A2M,COL6A2,IGFBP4,LBP,MMP2,MYH9,TIMP2,VCAM1 |
| Primary Immunodeficiency Signaling | 4.04 | 0.0833 | - | IGHG2,IGHG4,IGLC3,IGLC7 |
| FAT10 Signaling Pathway | 3.84 | 0.0741 | - | PSMA1,PSMA3,PSMA6,PSMB7 |
| Polyamine Regulation in Colon Cancer | 3.66 | 0.0667 | - | PSMA1,PSMA3,PSMA6,PSMB7 |
| Pyruvate Fermentation to Lactate | 3.63 | 0.4 | - | LDHA,LDHB |
| Phagosome Formation | 3.22 | 0.0362 | - | IGHG2,IGHG4,IGLC3,IGLC7,MRC1 |
| Coagulation System | 3.17 | 0.0857 | - | A2M,F10,TFPI |
| BAG2 Signaling Pathway | 3.16 | 0.0494 | - | PSMA1,PSMA3,PSMA6,PSMB7 |
| LPS/IL-1 Mediated Inhibition of RXR Function | 3.12 | 0.0273 | -1 | APOC1,APOC2,APOC4,APOE,LBP,PLTP |
| Inhibition of Matrix Metalloproteases | 3.07 | 0.0789 | - | A2M,MMP2,TIMP2 |
| Pentose Phosphate Pathway | 2.98 | 0.2 | - | PGD,TKT |
| Leukocyte Extravasation Signaling | 2.62 | 0.0265 | 0 | MMP2,ROCK2,TIMP2,VCAM1,VCL |
| Extrinsic Prothrombin Activation Pathway | 2.56 | 0.125 | - | F10,TFPI |
| Role of Macrophages, Fibroblasts and Endothelial Cells in Rheumatoid Arthritis | 2.35 | 0.0192 | - | IGHG2,IGHG4,IGLC3,IGLC7,ROCK2,VCAM1 |
| Inhibition of ARE-Mediated mRNA Degradation Pathway | 2.1 | 0.0252 | - | PSMA1,PSMA3,PSMA6,PSMB7 |
| Complement System | 1.87 | 0.0556 | - | C3,SERPING1 |
| B Cell Receptor Signaling | 1.85 | 0.0212 | - | IGHG2,IGHG4,IGLC3,IGLC7 |
| ILK Signaling | 1.82 | 0.0207 | - | DSP,MYH9,RSU1,VCL |
| Agranulocyte Adhesion and Diapedesis | 1.79 | 0.0203 | - | MMP2,MYH9,SELP,VCAM1 |
| Airway Pathology in Chronic Obstructive Pulmonary Disease | 1.74 | 0.0265 | - | APOD,APOM,MMP2 |
| Pentose Phosphate Pathway (Oxidative Branch) | 1.71 | 0.25 | - | PGD |
| Glutathione Redox Reactions II | 1.71 | 0.25 | - | GSR |
| Hematopoiesis from Pluripotent Stem Cells | 1.69 | 0.019 | - | IGHG2,IGHG4,IGLC3,IGLC7 |
| Allograft Rejection Signaling | 1.61 | 0.0179 | - | IGHG2,IGHG4,IGLC3,IGLC7 |
| Autoimmune Thyroid Disease Signaling | 1.61 | 0.0179 | - | IGHG2,IGHG4,IGLC3,IGLC7 |
| White Adipose Tissue Browning Pathway | 1.54 | 0.0222 | - | CAMP,LDHA,LDHB |
| Pentose Phosphate Pathway (Non-oxidative Branch) | 1.54 | 0.167 | - | TKT |
| Actin Cytoskeleton Signaling | 1.51 | 0.0167 | - | LBP,MYH9,ROCK2,VCL |
| Adenine and Adenosine Salvage III | 1.47 | 0.143 | - | ADA2 |
| Purine Ribonucleosides Degradation to Ribose-1-phosphate | 1.47 | 0.143 | - | ADA2 |
| Glycogen Biosynthesis II (from UDP-D-Glucose) | 1.47 | 0.143 | - | UGP2 |
| Heparan Sulfate Biosynthesis (Late Stages) | 1.47 | 0.0339 | - | EXT1,EXT2 |
| Semaphorin Neuronal Repulsive Signaling Pathway | 1.46 | 0.0207 | - | CSPG4,ROCK2,VCAN |
| Sucrose Degradation V (Mammalian) | 1.41 | 0.125 | - | ALDOB |
| Communication between Innate and Adaptive Immune Cells | 1.38 | 0.0152 | - | IGHG2,IGHG4,IGLC3,IGLC7 |
| Epithelial Adherens Junction Signaling | 1.38 | 0.0192 | - | MYH9,NOTCH3,VCL |
| Heparan Sulfate Biosynthesis | 1.38 | 0.0303 | - | EXT1,EXT2 |
| Protein Ubiquitination Pathway | 1.35 | 0.0148 | - | PSMA1,PSMA3,PSMA6,PSMB7 |
| Huntington's Disease Signaling | 1.33 | 0.0145 | - | PSMA1,PSMA3,PSMA6,PSMB7 |
| Glioma Invasiveness Signaling | 1.32 | 0.0282 | - | MMP2,TIMP2 |
| Granulocyte Adhesion and Diapedesis | 1.26 | 0.0172 | - | MMP2,SELP,VCAM1 |
| VDR/RXR Activation | 1.26 | 0.026 | - | CAMP,IGFBP6 |
| Chondroitin Sulfate Degradation (Metazoa) | 1.18 | 0.0714 | - | GM2A |
| Colanic Acid Building Blocks Biosynthesis | 1.18 | 0.0714 | - | UGP2 |
| Dermatan Sulfate Degradation (Metazoa) | 1.15 | 0.0667 | - | GM2A |
| Adenosine Nucleotides Degradation II | 1.12 | 0.0625 | - | ADA2 |
| HIF1α Signaling | 1.1 | 0.0147 | - | LDHA,LDHB,MMP2 |
| IL-8 Signaling | 1.09 | 0.0145 | - | MMP2,ROCK2,VCAM1 |
| VEGF Signaling | 1.08 | 0.0206 | - | ROCK2,VCL |
| Purine Nucleotides Degradation II (Aerobic) | 1.05 | 0.0526 | - | ADA2 |
| IGF-1 Signaling | 1.03 | 0.0192 | - | IGFBP4,IGFBP6 |
| Dendritic Cell Maturation | 1 | 0.0112 | -2 | IGHG2,IGHG4,IGLC3,IGLC7 |
| Glutathione Redox Reactions I | 0.971 | 0.0435 | - | GSR |
| Glycolysis I | 0.951 | 0.0417 | - | ALDOB |
| Gluconeogenesis I | 0.936 | 0.04 | - | ALDOB |
| Systemic Lupus Erythematosus Signaling | 0.903 | 0.0103 | - | IGHG2,IGHG4,IGLC3,IGLC7 |
| IL-6 Signaling | 0.883 | 0.0156 | - | A2M,LBP |
| Thyroid Hormone Metabolism II (via Conjugation and/or Degradation) | 0.836 | 0.0312 | - | EXT2 |
| Endocannabinoid Cancer Inhibition Pathway | 0.812 | 0.0141 | - | MMP2,ROCK2 |
| NAD Signaling Pathway | 0.796 | 0.0138 | - | LDHA,LDHB |
| Dilated Cardiomyopathy Signaling Pathway | 0.793 | 0.0137 | - | DSC2,MYH9 |
| Notch Signaling | 0.777 | 0.027 | - | NOTCH3 |
| Phospholipase C Signaling | 0.764 | 0.00907 | - | IGHG2,IGHG4,IGLC3,IGLC7 |
| Intrinsic Prothrombin Activation Pathway | 0.738 | 0.0244 | - | F10 |
| HOTAIR Regulatory Pathway | 0.735 | 0.0126 | - | MMP2,ROCK2 |
| Oncostatin M Signaling | 0.719 | 0.0233 | - | CHI3L1 |
| Germ Cell-Sertoli Cell Junction Signaling | 0.708 | 0.012 | - | A2M,VCL |
| iNOS Signaling | 0.693 | 0.0217 | - | LBP |
| PFKFB4 Signaling Pathway | 0.693 | 0.0217 | - | TKT |
| Ephrin A Signaling | 0.684 | 0.0213 | - | ROCK2 |
| Tight Junction Signaling | 0.666 | 0.0113 | - | MYH9,VCL |
| Tumor Microenvironment Pathway | 0.662 | 0.0112 | - | CSPG4,MMP2 |
| Nicotine Degradation III | 0.66 | 0.02 | - | EXT2 |
| PPARα/RXRα Activation | 0.652 | 0.011 | - | ADIPOQ,APOA1 |
| Melatonin Degradation I | 0.638 | 0.0189 | - | EXT2 |
| Lymphotoxin β Receptor Signaling | 0.631 | 0.0185 | - | VCAM1 |
| Nicotine Degradation II | 0.623 | 0.0182 | - | EXT2 |
| Serotonin Degradation | 0.618 | 0.0179 | - | EXT2 |
| Regulation of the Epithelial-Mesenchymal Transition Pathway | 0.611 | 0.0104 | - | MMP2,NOTCH3 |
| Adrenomedullin signaling pathway | 0.607 | 0.0103 | - | C3,MMP2 |
| Superpathway of Melatonin Degradation | 0.604 | 0.0172 | - | EXT2 |
| Semaphorin Signaling in Neurons | 0.597 | 0.0169 | - | ROCK2 |
| PCP pathway | 0.592 | 0.0167 | - | ROCK2 |
| Sertoli Cell-Sertoli Cell Junction Signaling | 0.583 | 0.0099 | - | A2M,VCL |
| Activation of IRF by Cytosolic Pattern Recognition Receptors | 0.573 | 0.0159 | - | PPIB |
| NRF2-mediated Oxidative Stress Response | 0.556 | 0.00948 | - | GSR,PPIB |
| Remodeling of Epithelial Adherens Junctions | 0.556 | 0.0152 | - | VCL |
| RhoGDI Signaling | 0.553 | 0.00943 | - | MYH9,ROCK2 |
| Growth Hormone Signaling | 0.529 | 0.0141 | - | A2M |
| IL-10 Signaling | 0.524 | 0.0139 | - | LBP |
| Ephrin B Signaling | 0.524 | 0.0139 | - | ROCK2 |
| Hypoxia Signaling in the Cardiovascular System | 0.524 | 0.0139 | - | LDHA |
| Estrogen Receptor Signaling | 0.509 | 0.00756 | - | IGF2R,MMP2,ROCK2 |
| Macropinocytosis Signaling | 0.504 | 0.0132 | - | MRC1 |
| Toll-like Receptor Signaling | 0.5 | 0.013 | - | LBP |
| Chemokine Signaling | 0.5 | 0.013 | - | ROCK2 |
| Xenobiotic Metabolism AHR Signaling Pathway | 0.491 | 0.0127 | - | PON1 |
| BEX2 Signaling Pathway | 0.487 | 0.0125 | - | MMP2 |
| TR/RXR Activation | 0.47 | 0.0119 | - | F10 |
| PEDF Signaling | 0.47 | 0.0119 | - | ROCK2 |
| LPS-stimulated MAPK Signaling | 0.465 | 0.0118 | - | LBP |
| Regulation of Cellular Mechanics by Calpain Protease | 0.453 | 0.0114 | - | VCL |
| Actin Nucleation by ARP-WASP Complex | 0.441 | 0.011 | - | ROCK2 |
| Paxillin Signaling | 0.389 | 0.00943 | - | VCL |
| Sirtuin Signaling Pathway | 0.389 | 0.00704 | - | LDHA,LDHB |
| Senescence Pathway | 0.372 | 0.0068 | - | SAA1,SAA4 |
| Bladder Cancer Signaling | 0.366 | 0.00877 | - | MMP2 |
| Role of Tissue Factor in Cancer | 0.364 | 0.0087 | - | F10 |
| FAK Signaling | 0.361 | 0.00862 | - | VCL |
| Cholecystokinin/Gastrin-mediated Signaling | 0.358 | 0.00855 | - | ROCK2 |
| NGF Signaling | 0.358 | 0.00855 | - | ROCK2 |
| Th1 Pathway | 0.358 | 0.00855 | - | NOTCH3 |
| Glioma Signaling | 0.344 | 0.0082 | - | IGF2R |
| RhoA Signaling | 0.344 | 0.0082 | - | ROCK2 |
| GP6 Signaling Pathway | 0.339 | 0.00806 | - | COL6A2 |
| Reelin Signaling in Neurons | 0.336 | 0.008 | - | APOE |
| CCR3 Signaling in Eosinophils | 0.321 | 0.00763 | - | ROCK2 |
| Gα12/13 Signaling | 0.317 | 0.00752 | - | ROCK2 |
| Th2 Pathway | 0.317 | 0.00752 | - | NOTCH3 |
| STAT3 Pathway | 0.312 | 0.00741 | - | IGF2R |
| PI3K Signaling in B Lymphocytes | 0.308 | 0.0073 | - | C3 |
| Apelin Endothelial Signaling Pathway | 0.306 | 0.00725 | - | VCAM1 |
| Glucocorticoid Receptor Signaling | 0.304 | 0.00556 | - | A2M,MMP2,VCAM1 |
| Cellular Effects of Sildenafil (Viagra) | 0.293 | 0.00694 | - | MYH9 |
| Role of Pattern Recognition Receptors in Recognition of Bacteria and Viruses | 0.289 | 0.00685 | - | C3 |
| Factors Promoting Cardiogenesis in Vertebrates | 0.289 | 0.00685 | - | ROCK2 |
| Type II Diabetes Mellitus Signaling | 0.281 | 0.00667 | - | ADIPOQ |
| PTEN Signaling | 0.281 | 0.00667 | - | IGF2R |
| Ovarian Cancer Signaling | 0.269 | 0.00641 | - | MMP2 |
| HMGB1 Signaling | 0.264 | 0.00629 | - | VCAM1 |
| Mitochondrial Dysfunction | 0.255 | 0.0061 | - | GSR |
| CXCR4 Signaling | 0.255 | 0.0061 | - | ROCK2 |
| Gαq Signaling | 0.253 | 0.00606 | - | ROCK2 |
| Th1 and Th2 Activation Pathway | 0.25 | 0.00599 | - | NOTCH3 |
| D-myo-inositol (1,4,5,6)-Tetrakisphosphate Biosynthesis | 0.242 | 0.00581 | - | CILP |
| D-myo-inositol (3,4,5,6)-tetrakisphosphate Biosynthesis | 0.242 | 0.00581 | - | CILP |
| IL-17 Signaling | 0.231 | 0.00559 | - | MMP2 |
| Hepatic Cholestasis | 0.225 | 0.00546 | - | LBP |
| 3-phosphoinositide Degradation | 0.223 | 0.00543 | - | CILP |
| GNRH Signaling | 0.222 | 0.00541 | - | MMP2 |
| D-myo-inositol-5-phosphate Metabolism | 0.218 | 0.00532 | - | CILP |
| HER-2 Signaling in Breast Cancer | 0.213 | 0.00521 | - | MMP2 |
| Regulation Of The Epithelial Mesenchymal Transition By Growth Factors Pathway | 0.213 | 0.00521 | - | MMP2 |
| Natural Killer Cell Signaling | 0.211 | 0.00518 | - | PVR |
| 3-phosphoinositide Biosynthesis | 0.204 | 0.00505 | - | CILP |
| Ephrin Receptor Signaling | 0.203 | 0.00503 | - | ROCK2 |
| Axonal Guidance Signaling | 0 | 0.0041 | - | MMP2,ROCK2 |
| Thrombin Signaling | 0 | 0.00488 | - | ROCK2 |
| Cardiac Hypertrophy Signaling | 0 | 0.00395 | - | ROCK2 |
| CREB Signaling in Neurons | 0 | 0.00169 | - | IGF2R |
| Colorectal Cancer Metastasis Signaling | 0 | 0.00382 | - | MMP2 |
| AMPK Signaling | 0 | 0.00415 | - | ADIPOQ |
| Protein Kinase A Signaling | 0 | 0.00258 | - | ROCK2 |
| Breast Cancer Regulation by Stathmin1 | 0 | 0.00172 | - | MMP2 |
| Signaling by Rho Family GTPases | 0 | 0.00377 | - | ROCK2 |
| Superpathway of Inositol Phosphate Compounds | 0 | 0.00441 | - | CILP |
| Integrin Signaling | 0 | 0.00488 | - | VCL |
| NF-κB Signaling | 0 | 0.0029 | - | IGF2R |
| Osteoarthritis Pathway | 0 | 0.00435 | - | ADIPOQ |
| Neuroinflammation Signaling Pathway | 0 | 0.00328 | - | VCAM1 |
| Cardiac Hypertrophy Signaling (Enhanced) | 0 | 0.0019 | - | ROCK2 |
| Synaptogenesis Signaling Pathway | 0 | 0.00326 | - | APOE |
| Systemic Lupus Erythematosus In T Cell Signaling Pathway | 0 | 0.00263 | - | ROCK2 |
| Hepatic Fibrosis Signaling Pathway | 0 | 0.00485 | - | ROCK2,VCAM1 |
| Calcium Signaling | 0 | 0.00481 | - | MYH9 |

**Table S2B. Disease and bio-function analysis**

| Categories | Diseases or Functions Annotation | p-value | LOG(p-value) | Predicted Activation State | Activation z-score | Molecules | # Molecules |
| --- | --- | --- | --- | --- | --- | --- | --- |
| Cellular Movement | Cell movement | 4.12E-19 | 18.38510278 |  | 0.773 | A2M,ADIPOQ,ANTXR1,APOA1,APOB,APOD,APOE,C3,CAMP,CCN5,CD84,CHAD,CHI3L1,CST3,DSC2,DSP,EFEMP1,F10,FBLN5,FBN1,IGFBP4,IGFBP6,IGHV3-7,IGKC,IGKV4-1,IGLC3,IGLC7,IGLV1-51,LCP1,LPA,MCAM,MMP2,MYH9,NOTCH3,PDLIM1,PPIB,PVR,ROCK2,RSU1,SAA1,SELP,SPARCL1,ST6GAL1,TAGLN2,TFPI,TIMP2,VCAM1,VCAN,VCL | 49 |
| Lipid Metabolism,Molecular Transport,Small Molecule Biochemistry | Cholesterol transport | 1.27E-16 | 15.89619628 |  | 1.823 | ADIPOQ,APOA1,APOA4,APOB,APOC1,APOC2,APOC3,APOE,APOM,NPC2,PLTP,PON1,SAA1 | 13 |
| Cellular Movement | Migration of cells | 4.17E-16 | 15.37986395 |  | 0.231 | A2M,ADIPOQ,APOA1,APOB,APOD,APOE,C3,CAMP,CCN5,CD84,CHI3L1,CST3,DSP,EFEMP1,F10,FBLN5,IGFBP4,IGFBP6,IGHV3-7,IGKC,IGKV4-1,IGLC3,IGLC7,IGLV1-51,LCP1,LPA,MCAM,MMP2,MYH9,PDLIM1,PPIB,PVR,ROCK2,RSU1,SAA1,SELP,SPARCL1,ST6GAL1,TFPI,TIMP2,VCAM1,VCAN,VCL | 43 |
| Protein Synthesis | Metabolism of cellular protein | 5.86E-16 | 15.23210238 |  |  | ADA2,APCS,APOA1,APOA4,APOB,APOE,C3,CST3,FBN1,IGFBP4,IGFBP6,LTBP1,MMP2,PCSK9,SAA1,SPARCL1,VCAN | 17 |
| Lipid Metabolism,Molecular Transport,Small Molecule Biochemistry | Efflux of cholesterol | 5.94E-16 | 15.22621356 | Increased | 2.721 | ADIPOQ,APOA1,APOA4,APOC1,APOC2,APOC3,APOE,APOM,NPC2,PLTP,PON1,SAA1 | 12 |
| Cell-To-Cell Signaling and Interaction | Adhesion of blood cells | 1.09E-14 | 13.9625735 |  | -1.21 | A2M,ADIPOQ,APCS,APOA4,APOE,C3,CAMP,F10,FBN1,MCAM,MRC1,PLTP,PPIB,SAA1,SELP,SERPING1,VCAM1 | 17 |
| Cellular Function and Maintenance | Receptor-mediated endocytosis | 2.05E-14 | 13.68824614 |  | -0.655 | ANTXR1,APOA1,APOB,APOC1,APOC2,APOC3,APOE,IGF2R,IGHV3-7,IGKC,IGKV4-1,IGLC3,IGLC7,IGLV1-51,MRC1,SAA1 | 16 |
| Lipid Metabolism,Molecular Transport,Small Molecule Biochemistry | Transport of lipid | 5.35E-14 | 13.27164622 |  | 1.837 | ADIPOQ,APOA1,APOA4,APOB,APOC1,APOC2,APOC3,APOE,APOM,LBP,NPC2,PLTP,PON1,SAA1 | 14 |
| Cellular Movement,Immune Cell Trafficking | Leukocyte migration | 1.42E-13 | 12.84771166 |  | -0.07 | APOA1,APOB,APOD,C3,CAMP,CD84,IGHV3-7,IGKC,IGKV4-1,IGLC3,IGLC7,IGLV1-51,LCP1,LPA,MCAM,MYH9,PPIB,ROCK2,SAA1,SELP,TIMP2,VCAM1 | 22 |
| Cardiovascular Disease,Organismal Injury and Abnormalities | Atherosclerosis | 6.72E-13 | 12.17263073 |  |  | ADIPOQ,APCS,APOA1,APOB,APOC2,APOC3,APOE,CST3,F10,LPA,MMP2,PCSK9,PON1,SELP,TIMP2,TKT,VCAM1,VCAN,VSIG4 | 19 |
| Cardiovascular Disease,Organismal Injury and Abnormalities | Calcification of aortic valve | 7.39E-13 | 12.13135556 |  |  | APOA1,APOA4,APOB,APOC3,APOD,APOM,MMP2,TIMP2 | 8 |
| Cardiovascular Disease,Organismal Injury and Abnormalities | Aortic valve disease | 7.97E-13 | 12.09854168 |  |  | APOA1,APOA4,APOB,APOC3,APOD,APOM,DSP,F10,MMP2,TIMP2,VCAM1 | 11 |
| Neurological Disease,Organismal Injury and Abnormalities,Psychological Disorders | Dementia | 1.25E-12 | 11.90308999 |  |  | A2M,APOA1,APOA4,APOB,APOC1,APOC2,APOC3,APOC4,APOD,APOE,C3,CHI3L1,CST3,DCD,GM2A,IGFBP6,IGKC,LPA,MMP2,MRC1,NOTCH3,PLTP,PON1,ROCK2,SELP,SPARCL1 | 26 |
| Cellular Function and Maintenance | Endocytosis | 1.45E-12 | 11.838632 |  | -0.609 | ANTXR1,APCS,APOA1,APOB,APOC1,APOC2,APOC3,APOE,C3,CAMP,CD5L,IGF2R,IGHV3-7,IGKC,IGKV4-1,IGLC3,IGLC7,IGLV1-51,MRC1,SAA1 | 20 |
| Metabolic Disease,Organismal Injury and Abnormalities | Amyloidosis | 1.75E-12 | 11.75696195 |  |  | A2M,APOA1,APOA4,APOC1,APOC2,APOC3,APOC4,APOD,APOE,C3,CHI3L1,CST3,DCD,GM2A,IGFBP6,IGKC,LPA,MMP2,MRC1,PLTP,PON1,ROCK2,SAA1,SELP,SPARCL1 | 25 |
| Cardiovascular Disease,Organismal Injury and Abnormalities | Occlusion of artery | 1.96E-12 | 11.70774393 |  |  | ADIPOQ,APCS,APOA1,APOB,APOC2,APOC3,APOE,CST3,F10,LPA,MMP2,PCSK9,PON1,SELP,TFPI,TIMP2,TKT,VCAM1,VCAN,VSIG4 | 20 |
| Lipid Metabolism,Molecular Transport,Small Molecule Biochemistry | Efflux of phospholipid | 2.7E-12 | 11.56863624 |  |  | APOA1,APOA4,APOC1,APOC2,APOC3,APOE,PLTP | 7 |
| Metabolic Disease,Organismal Injury and Abnormalities | Disorder of lipid metabolism | 3.82E-12 | 11.41793664 |  |  | ADIPOQ,APOA1,APOB,APOC2,APOC3,APOE,FBN1,GM2A,LBP,LPA,NPC2,PCSK9,PON1,SELP,VCL | 15 |
| Metabolic Disease,Neurological Disease,Organismal Injury and Abnormalities,Psychological Disorders | Alzheimer disease | 5.27E-12 | 11.27818938 |  |  | A2M,APOA1,APOA4,APOC1,APOC2,APOC3,APOC4,APOD,APOE,C3,CHI3L1,CST3,DCD,GM2A,IGFBP6,IGKC,LPA,MMP2,MRC1,PLTP,PON1,ROCK2,SELP,SPARCL1 | 24 |
| Humoral Immune Response,Inflammatory Response | Complement activation | 5.42E-12 | 11.26600071 |  |  | APOE,C3,COLEC10,IGHG2,IGHG4,IGHV3-7,IGKC,IGKV4-1,IGLC3,IGLC7,IGLV1-51 | 11 |
| Cardiovascular Disease,Organismal Injury and Abnormalities | Left ventricular dysfunction | 1.74E-11 | 10.75945075 |  |  | APOA1,APOA4,APOB,APOC3,APOD,APOM,DSP,F10,MMP2,TIMP2,VCL | 11 |
| Cardiovascular Disease,Organismal Injury and Abnormalities | Abnormality of heart ventricle | 2.86E-11 | 10.54363397 |  |  | APOA1,APOA4,APOB,APOC3,APOD,APOM,DSC2,DSP,F10,MMP2,TIMP2,VCAM1,VCL | 13 |
| Hematological Disease,Metabolic Disease,Organismal Injury and Abnormalities | Dyslipidemia | 3.53E-11 | 10.45222529 |  |  | ADIPOQ,APOA1,APOB,APOC2,APOC3,APOE,LBP,LPA,PCSK9,PON1,SELP | 11 |
| Cardiovascular Disease,Organismal Injury and Abnormalities | Abnormality of left ventricle | 3.85E-11 | 10.41453927 |  |  | APOA1,APOA4,APOB,APOC3,APOD,APOM,DSP,F10,MMP2,TIMP2,VCAM1,VCL | 12 |
| Lipid Metabolism,Molecular Transport,Small Molecule Biochemistry | Transport of phospholipid | 3.95E-11 | 10.4034029 |  |  | APOA1,APOA4,APOC1,APOC2,APOC3,APOE,NPC2,PLTP | 8 |
| Humoral Immune Response,Inflammatory Response | Classical complement pathway | 4.53E-11 | 10.3439018 |  |  | APOE,IGHG2,IGHG4,IGHV3-7,IGKC,IGKV4-1,IGLC3,IGLC7,IGLV1-51 | 9 |
| Cell-To-Cell Signaling and Interaction,Hematological System Development and Function,Inflammatory Response | Adhesion of blood platelets | 6.12E-11 | 10.21324858 | Decreased | -2.425 | APOA4,C3,F10,FBN1,PPIB,SAA1,SELP | 7 |
| Lipid Metabolism,Small Molecule Biochemistry | Homeostasis of lipid | 6.77E-11 | 10.16941133 |  |  | APOA1,APOA4,APOB,APOC2,APOC3,APOC4,APOE,NPC2,PCSK9 | 9 |
| Cardiovascular Disease,Hematological Disease,Metabolic Disease,Organismal Injury and Abnormalities | Hypercholesterolemia | 8.75E-11 | 10.05799195 |  |  | APOA1,APOB,APOC2,APOC3,APOE,LBP,LPA,PCSK9,SELP | 9 |
| Cardiovascular Disease,Hereditary Disorder,Organismal Injury and Abnormalities | Familial cardiovascular disease | 9.19E-11 | 10.03668449 |  |  | ADA2,APOA1,APOB,APOC2,APOC3,APOE,C3,CST3,DSC2,DSP,F10,FBN1,LPA,NOTCH3,PCSK9,PON1,SELP,TKT,VCAN,VCL | 20 |
| Cellular Compromise,Inflammatory Response | Degranulation of cells | 3.81E-10 | 9.419075024 |  | 1.117 | A2M,ADA2,APOA1,C3,CAMP,CD84,CHI3L1,CST3,DSP,GM2A,IGF2R,NPC2,PSMB7,PVR,SELP,SERPING1,TAGLN2,TIMP2,VCL | 19 |
| Neurological Disease,Organismal Injury and Abnormalities | Progressive encephalopathy | 4.11E-10 | 9.386158178 |  |  | A2M,APOA1,APOA4,APOC1,APOC2,APOC3,APOC4,APOD,APOE,C3,CHI3L1,CST3,DCD,GM2A,GSR,IGFBP6,IGKC,LDHA,LDHB,LPA,MCAM,MMP2,MRC1,PLTP,PON1,ROCK2,SELP,SPARCL1 | 28 |
| Endocrine System Disorders,Gastrointestinal Disease,Metabolic Disease,Organismal Injury and Abnormalities | Diabetes mellitus | 4.45E-10 | 9.351639989 |  |  | ADIPOQ,APOA1,APOB,APOC1,APOC3,APOD,APOE,APOM,C3,CHI3L1,COL6A2,CRHBP,F10,FBLN5,LPA,LTBP1,MYH9,PCSK9,PON1,PSMA1,PSMA3,PSMB7,SELP,TFPI,VCAM1 | 25 |
| Neurological Disease | Progressive neurological disorder | 4.87E-10 | 9.312471039 |  |  | A2M,ANTXR1,APOA1,APOA4,APOC1,APOC2,APOC3,APOC4,APOD,APOE,C3,CHI3L1,CST3,DCD,GM2A,GSR,IGFBP6,IGKC,LDHA,LDHB,LPA,MCAM,MMP2,MRC1,PLTP,PON1,ROCK2,SELP,SERPING1,SPARCL1 | 30 |
| Metabolic Disease,Organismal Injury and Abnormalities | Glucose metabolism disorder | 5.2E-10 | 9.283996656 |  |  | ADIPOQ,APOA1,APOB,APOC1,APOC3,APOD,APOE,APOM,C3,CHI3L1,COL6A2,CRHBP,F10,FBLN5,LBP,LPA,LTBP1,MYH9,PCSK9,PON1,PSMA1,PSMA3,PSMB7,SELP,TFPI,VCAM1 | 26 |
| Cardiovascular Disease,Hematological Disease,Hereditary Disorder,Metabolic Disease,Organismal Injury and Abnormalities | Familial hypercholesterolemia | 7.61E-10 | 9.118615343 |  |  | APOA1,APOB,APOC2,APOC3,APOE,LPA,PCSK9 | 7 |
| Infectious Diseases | Severe COVID-19 | 2.07E-09 | 8.684029655 |  |  | APCS,APOA1,APOD,APOM,C3,IGLC3,SAA1,SAA4,TAGLN2,VCAM1,VCAN | 11 |
| Lipid Metabolism,Small Molecule Biochemistry,Vitamin and Mineral Metabolism | Homeostasis of cholesterol | 4.08E-09 | 8.389339837 |  |  | APOA1,APOA4,APOB,APOC3,APOE,NPC2,PCSK9 | 7 |
| Cell-To-Cell Signaling and Interaction,Hematological System Development and Function | Interaction of leukocytes | 5.09E-09 | 8.293282218 |  | -0.458 | A2M,ADIPOQ,APOA4,APOE,CAMP,MCAM,MRC1,PLTP,SAA1,SELP,SERPING1,VCAM1 | 12 |
| Cardiovascular Disease,Organismal Injury and Abnormalities | Acute coronary syndrome | 8.94E-09 | 8.048662481 |  |  | ADIPOQ,APOA1,APOB,APOE,C3,F10,LPA,PCSK9,PON1,SELP,TFPI | 11 |
| Cell-To-Cell Signaling and Interaction,Hematological System Development and Function | Interaction of phagocytes | 9.02E-09 | 8.044793462 |  | -0.127 | A2M,ADIPOQ,APOE,CAMP,MRC1,PLTP,SAA1,SELP,VCAM1 | 9 |
| Cellular Movement | Cell movement of tumor cell lines | 1.15E-08 | 7.93930216 |  | 0.978 | A2M,ADIPOQ,C3,CAMP,CCN5,CST3,DSP,EFEMP1,FBLN5,IGFBP4,IGFBP6,LCP1,MCAM,MMP2,MYH9,NOTCH3,PDLIM1,PVR,RSU1,SELP,SPARCL1,ST6GAL1,TAGLN2,TFPI,TIMP2,VCAM1,VCAN | 27 |
| Cell-To-Cell Signaling and Interaction,Hematological System Development and Function,Immune Cell Trafficking | Adhesion of immune cells | 1.57E-08 | 7.804100348 |  | -1.005 | A2M,ADIPOQ,APOA4,APOE,CAMP,MCAM,MRC1,PLTP,SELP,SERPING1,VCAM1 | 11 |
| Cell-To-Cell Signaling and Interaction,Hematological System Development and Function | Interaction of antigen presenting cells | 0.000000021 | 7.677780705 |  | 0.152 | A2M,ADIPOQ,APOE,CAMP,MRC1,VCAM1 | 6 |
| Lipid Metabolism,Small Molecule Biochemistry | Fatty acid metabolism | 2.41E-08 | 7.617982957 |  | 1.904 | ADIPOQ,APOA1,APOA4,APOB,APOC1,APOC2,APOC3,APOE,APOM,CAMP,LBP,NPC2,PLTP,PON1,SAA1 | 15 |
| Cellular Movement,Hematological System Development and Function,Immune Cell Trafficking | Cell movement of mononuclear leukocytes | 2.64E-08 | 7.578396073 |  | 0.423 | APOA1,APOD,CAMP,LCP1,LPA,MCAM,MYH9,PPIB,SAA1,SELP,TIMP2,VCAM1 | 12 |
| Cardiovascular Disease,Organismal Injury and Abnormalities | Peripheral vascular disease | 2.75E-08 | 7.560667306 |  |  | ADA2,ADIPOQ,APOA1,APOB,APOE,CST3,F10,LCP1,LPA,NOTCH3,PCSK9,PLTP,ST6GAL1,TFPI,VCAM1,VSIG4 | 16 |
| Developmental Disorder,Hereditary Disorder,Metabolic Disease,Organismal Injury and Abnormalities | Familial amyloidosis | 2.78E-08 | 7.555955204 |  |  | A2M,APOA1,APOA4,APOC1,APOC2,APOE,CST3 | 7 |
| Hematological System Development and Function | Hemostasis | 3.06E-08 | 7.514278574 | Decreased | -2.755 | A2M,APOA4,APOE,C3,F10,FBN1,LMAN1,PPIB,SAA1,SELP,SERPING1,TFPI | 12 |
| Cellular Movement,Hematological System Development and Function,Immune Cell Trafficking | Cell movement of leukocytes | 3.21E-08 | 7.493494968 |  | -0.028 | APOA1,APOD,C3,CAMP,LCP1,LPA,MCAM,MYH9,PPIB,ROCK2,SAA1,SELP,TIMP2,VCAM1 | 14 |
| Tissue Development | Organization of extracellular matrix | 4.29E-08 | 7.367542708 |  |  | A2M,COL6A2,FBLN5,FBN1,LCP1,MMP2,SPINK5,TIMP2,VCAM1,VCAN | 10 |
| Cardiovascular Disease,Organismal Injury and Abnormalities | Acute coronary event | 4.73E-08 | 7.325138859 |  |  | ADIPOQ,APOA1,C3,DSC2,F10,PCSK9,PON1,SELP,TFPI,VCL | 10 |
| Infectious Diseases | COVID-19 | 6.17E-08 | 7.209714836 |  |  | APCS,APOA1,APOD,APOM,C3,F10,IGLC3,SAA1,SAA4,TAGLN2,VCAM1,VCAN | 12 |
| Lipid Metabolism,Molecular Transport,Small Molecule Biochemistry | Secretion of triacylglycerol | 0.000000068 | 7.167491087 |  |  | APOA1,APOA4,APOB,APOC3 | 4 |
| Lipid Metabolism,Small Molecule Biochemistry | Esterification of cholesterol | 0.000000103 | 6.987162775 | Increased | 2.236 | APOA1,APOA4,APOC1,APOE,C3 | 5 |
| Cardiovascular Disease,Hematological Disease,Metabolic Disease,Organismal Injury and Abnormalities | Fredrickson hyperlipoproteinemia | 0.000000103 | 6.987162775 |  |  | APOA1,APOB,APOC3,APOE,PCSK9 | 5 |
| Developmental Disorder,Hereditary Disorder,Metabolic Disease,Neurological Disease,Organismal Injury and Abnormalities,Psychological Disorders | Familial Alzheimer disease | 0.000000111 | 6.954677021 |  |  | A2M,APOA4,APOC1,APOC2,APOE,CST3 | 6 |
| Immunological Disease | Systemic autoimmune syndrome | 0.00000012 | 6.920818754 |  |  | ADA2,ADIPOQ,APOA1,APOE,APOM,C3,CAMP,CHI3L1,COL6A2,CST3,F10,GSR,IGKC,LBP,LCP1,LDHB,LPA,LTBP1,MMP2,MRC1,MYH9,PON1,SAA1,SELP,SERPING1,VCAM1,VSIG4 | 27 |
| Cell-To-Cell Signaling and Interaction,Hematological System Development and Function,Inflammatory Response | Binding of professional phagocytic cells | 0.000000121 | 6.91721463 |  | -0.452 | A2M,ADIPOQ,APOE,CAMP,MRC1,PLTP,SELP,VCAM1 | 8 |
| Cardiovascular Disease,Organismal Injury and Abnormalities | Infarction | 0.000000129 | 6.88941029 |  |  | ADIPOQ,APOA1,APOB,APOE,C3,F10,LPA,NOTCH3,PON1,PSMA6,SELP | 11 |
| Cardiovascular Disease,Organismal Injury and Abnormalities | Coronary artery disease | 0.000000138 | 6.860120914 |  |  | ADIPOQ,APOA1,APOB,APOC3,APOE,F10,LPA,PCSK9,PON1,SELP,TKT | 11 |
| Infectious Diseases | Viral Infection | 0.000000142 | 6.847711656 |  | 1.197 | ADIPOQ,APCS,APOA1,APOB,APOC1,APOD,APOE,APOM,C3,CAMP,CCN5,COLEC10,CST3,DCD,F10,FBN1,GM2A,IGF2R,IGLC3,MCAM,MRC1,PPIB,PSMA1,PSMA3,PVR,SAA1,SAA4,ST6GAL1,TAGLN2,TIMP2,TKT,VCAM1,VCAN | 33 |
| Ophthalmic Disease,Organismal Injury and Abnormalities | Exfoliative glaucoma | 0.000000157 | 6.804100348 |  |  | APOD,FBN1,LTBP1,SAA1,TIMP2 | 5 |
| Lipid Metabolism,Molecular Transport,Small Molecule Biochemistry | Reverse cholesterol transport | 0.000000177 | 6.752026734 |  |  | APOA1,APOA4,APOE,APOM | 4 |
| Lipid Metabolism,Molecular Transport,Small Molecule Biochemistry | Concentration of triacylglycerol | 0.000000183 | 6.73754891 |  |  | ADIPOQ,APOA1,APOB,APOC3,PLTP,PON1 | 6 |
| Lipid Metabolism,Small Molecule Biochemistry | Binding of lipid | 0.000000183 | 6.73754891 |  | 0.277 | APOA1,APOB,APOE,CAMP,LBP,SELP | 6 |
| Cellular Movement | Cell movement of myeloid cells | 0.000000211 | 6.675717545 |  | 0.617 | APOA1,C3,CAMP,LCP1,LPA,PPIB,ROCK2,SAA1,SELP,TIMP2,VCAM1 | 11 |
| Lipid Metabolism,Small Molecule Biochemistry,Vitamin and Mineral Metabolism | Metabolism of retinoid | 0.00000022 | 6.657577319 |  |  | APOA1,APOA4,APOB,APOC2,APOC3,APOE,APOM | 7 |
| Dermatological Diseases and Conditions,Organismal Injury and Abnormalities | Psoriasis | 0.000000234 | 6.630784143 |  |  | A2M,APOC1,APOD,APOE,C3,CAMP,CSPG4,DCD,DSC2,GM2A,MCAM,MMP2,PGD,PSMA3,PSMA6,TIMP2,VCAM1 | 17 |
| Cell-To-Cell Signaling and Interaction | Binding of tumor cell lines | 0.00000025 | 6.602059991 |  | -0.841 | APOE,DSP,EFEMP1,F10,FBN1,LPA,MCAM,MMP2,PCSK9,ROCK2,SELP,ST6GAL1,VCAM1,VCAN | 14 |
| Connective Tissue Disorders,Inflammatory Disease,Organismal Injury and Abnormalities,Skeletal and Muscular Disorders | Rheumatic Disease | 0.000000291 | 6.536107011 |  |  | ADIPOQ,APOA1,APOE,APOM,C3,CAMP,CHI3L1,CST3,F10,FBN1,GSR,IGKC,LBP,LCP1,LDHB,LTBP1,MMP2,MRC1,MYH9,PON1,SAA1,SELP,SERPING1,TIMP2,VCAM1,VSIG4 | 26 |
| Cellular Compromise,Inflammatory Response | Degranulation of leukocytes | 0.000000315 | 6.501689446 |  | 1.091 | ADA2,C3,CAMP,CD84,CHI3L1,CST3,DSP,GM2A,IGF2R,NPC2,PSMB7,PVR,TIMP2,VCL | 14 |
| Connective Tissue Disorders,Hereditary Disorder,Organismal Injury and Abnormalities | Hereditary connective tissue disorder | 0.00000034 | 6.468521083 |  |  | ADIPOQ,APCS,APOA1,APOE,C3,CILP,COL6A2,COLEC10,DSP,EXT1,EXT2,F10,FBLN5,FBN1,MMP2,MYH9,PPIB,VCAN,VCL | 19 |
| Lipid Metabolism,Molecular Transport,Small Molecule Biochemistry | Concentration of lipid | 0.000000358 | 6.446116973 |  | -0.737 | ADIPOQ,APOA1,APOB,APOC3,APOD,APOE,LPA,NPC2,PLTP,PON1,ROCK2 | 11 |
| Cardiovascular Disease,Hereditary Disorder,Organismal Injury and Abnormalities | Familial vascular disease | 0.000000358 | 6.446116973 |  |  | ADA2,C3,CST3,DSC2,F10,FBN1,NOTCH3,PCSK9,PON1,SELP,VCAN | 11 |
| Cellular Movement,Embryonic Development | Cell movement of embryonic cell lines | 0.000000404 | 6.393618635 |  | 1.143 | ADIPOQ,ANTXR1,APOA1,C3,CAMP,F10,ROCK2,SAA1 | 8 |
| Nutritional Disease | Body mass index | 0.000000406 | 6.391473966 |  |  | ADIPOQ,APOA1,APOA4,APOB,APOD,APOE,PON1 | 7 |
| Inflammatory Response,Organismal Injury and Abnormalities | Inflammation of organ | 0.000000465 | 6.332547047 |  |  | ADIPOQ,APOA1,APOB,APOE,C3,CAMP,CST3,DCD,DSP,F10,GSR,IGHG4,IGKC,LTBP1,MCAM,MYH9,PDLIM1,SELP,SPINK5,VCAM1,VCL | 21 |
| Connective Tissue Disorders,Immunological Disease,Inflammatory Disease,Inflammatory Response,Organismal Injury and Abnormalities,Skeletal and Muscular Disorders | Rheumatoid arthritis | 0.000000703 | 6.153044675 |  |  | ADIPOQ,APOA1,APOE,APOM,C3,CHI3L1,CST3,F10,GSR,IGKC,LBP,LCP1,LDHB,MMP2,MRC1,PON1,SAA1,VCAM1,VSIG4 | 19 |
| Developmental Disorder,Hereditary Disorder,Metabolic Disease,Organismal Injury and Abnormalities | Autosomal dominant amyloidosis | 0.00000072 | 6.142667504 |  |  | A2M,APOA1,APOE,CST3 | 4 |
| Lipid Metabolism,Small Molecule Biochemistry | Homeostasis of triacylglycerol | 0.00000072 | 6.142667504 |  |  | APOA1,APOC2,APOC3,APOC4 | 4 |
| Cell-To-Cell Signaling and Interaction | Adhesion of tumor cell lines | 0.000000722 | 6.141462802 |  | -0.108 | APOE,DSP,EFEMP1,F10,FBN1,MCAM,MMP2,ROCK2,SELP,ST6GAL1,VCAM1,VCAN | 12 |
| Cell-To-Cell Signaling and Interaction,Hematological System Development and Function,Immune Cell Trafficking | Binding of antigen presenting cells | 0.00000075 | 6.124938737 |  | 0.152 | A2M,ADIPOQ,APOE,CAMP,MRC1 | 5 |
| Inflammatory Response | Inflammatory response | 0.000000857 | 6.067019178 |  | 0.541 | ADIPOQ,APOA1,APOE,C1QTNF3,C3,CAMP,CRHBP,LBP,LCP1,LPA,PPIB,SAA1,SERPING1 | 13 |
| Cardiovascular Disease,Neurological Disease,Organismal Injury and Abnormalities | Cerebrovascular dysfunction | 0.000000919 | 6.036684489 |  |  | ADA2,ADIPOQ,APOA1,APOB,CST3,F10,LPA,NOTCH3,PCSK9,TFPI,VCAM1 | 11 |
| Hereditary Disorder,Neurological Disease,Organismal Injury and Abnormalities,Psychological Disorders | Familial dementia | 0.000000921 | 6.03574037 |  |  | A2M,APOA4,APOC1,APOC2,APOE,CST3,NOTCH3 | 7 |
| Cellular Movement | Migration of tumor cell lines | 0.000000968 | 6.014124643 |  | 0.766 | A2M,ADIPOQ,CAMP,CCN5,CST3,DSP,EFEMP1,FBLN5,IGFBP4,IGFBP6,MCAM,MMP2,MYH9,PDLIM1,PVR,RSU1,SPARCL1,ST6GAL1,TFPI,TIMP2,VCAM1,VCAN | 22 |
| Cardiovascular Disease,Organismal Injury and Abnormalities | Formation of blood clot | 0.00000114 | 5.943095149 |  |  | C3,F10,SELP | 3 |
| Inflammatory Disease | Chronic inflammatory disorder | 0.00000118 | 5.928117993 |  |  | ADIPOQ,APOA1,APOE,APOM,C3,CAMP,CHI3L1,CST3,F10,GSR,IGHG4,IGKC,LBP,LCP1,LDHB,MCAM,MMP2,MRC1,PDLIM1,PON1,SAA1,SELP,VCAM1,VSIG4 | 24 |
| Cellular Compromise,Inflammatory Response | Degranulation of phagocytes | 0.0000012 | 5.920818754 |  |  | ADA2,C3,CAMP,CD84,CHI3L1,CST3,DSP,GM2A,IGF2R,NPC2,PSMB7,TIMP2,VCL | 13 |
| Hematological Disease,Metabolic Disease,Organismal Injury and Abnormalities | Hypolipoproteinemia | 0.00000125 | 5.903089987 |  |  | APOA1,APOB,LPA,PCSK9 | 4 |
| Cellular Compromise,Inflammatory Response | Degranulation of myeloid cells | 0.0000014 | 5.853871964 |  |  | ADA2,C3,CAMP,CD84,CHI3L1,CST3,DSP,GM2A,IGF2R,NPC2,PSMB7,TIMP2,VCL | 13 |
| Cellular Movement | Cell movement of colorectal cancer cell lines | 0.00000163 | 5.787812396 |  | 0.718 | CAMP,EFEMP1,IGFBP4,IGFBP6,MMP2,PDLIM1,SELP,SPARCL1,ST6GAL1 | 9 |
| Cell-To-Cell Signaling and Interaction,Hematological System Development and Function,Immune Cell Trafficking,Inflammatory Response | Activation of macrophages | 0.00000169 | 5.772113295 |  | -0.447 | ADIPOQ,APOE,CAMP,LBP,SERPING1 | 5 |
| Cell-To-Cell Signaling and Interaction,Hematological System Development and Function | Activation of myeloid cells | 0.00000169 | 5.772113295 |  | -0.816 | ADIPOQ,APOE,C3,CAMP,CD84,LBP,SERPING1 | 7 |
| Ophthalmic Disease,Organismal Injury and Abnormalities | Open-angle glaucoma | 0.00000172 | 5.764471553 |  |  | APOD,APOE,FBN1,LTBP1,SAA1,TIMP2 | 6 |
| Infectious Diseases | Infection by RNA virus | 0.0000018 | 5.744727495 |  | 0.916 | ADIPOQ,APCS,APOA1,APOD,APOE,APOM,C3,CCN5,DCD,F10,GM2A,IGLC3,MRC1,PPIB,PSMA1,PSMA3,PVR,SAA1,SAA4,ST6GAL1,TAGLN2,VCAM1,VCAN | 23 |
| Connective Tissue Disorders,Organismal Injury and Abnormalities,Skeletal and Muscular Disorders | Non-traumatic arthropathy | 0.0000019 | 5.721246399 |  |  | ADIPOQ,APOA1,APOE,APOM,C3,CHI3L1,CST3,F10,GSR,IGKC,LBP,LCP1,LDHB,MMP2,MRC1,PON1,SAA1,TIMP2,VCAM1,VSIG4 | 20 |
| Cardiovascular Disease,Organismal Injury and Abnormalities,Skeletal and Muscular Disorders | Myocardial infarction | 0.00000202 | 5.694648631 |  |  | ADIPOQ,APOA1,APOE,C3,F10,LPA,PON1,PSMA6,SELP | 9 |
| Cell-To-Cell Signaling and Interaction | Binding of Candida albicans | 0.00000228 | 5.642065153 |  |  | C3,CAMP,MRC1 | 3 |
| Carbohydrate Metabolism,Small Molecule Biochemistry | Length of glycosaminoglycan | 0.00000228 | 5.642065153 |  |  | APOE,EXT1,EXT2 | 3 |
| Protein Synthesis | Metabolism of protein | 0.00000228 | 5.642065153 |  | 1.091 | ADA2,APCS,APOA1,APOA4,APOB,APOE,C3,CSPG4,CST3,FBN1,IGFBP4,IGFBP6,LTBP1,MMP2,MYH9,PCSK9,PPIB,SAA1,SPARCL1,VCAN | 20 |
| Cardiovascular Disease,Organismal Injury and Abnormalities | Ischemia of heart | 0.00000233 | 5.632644079 |  |  | ADIPOQ,APOA1,C3,F10,MMP2,PON1,SELP,TFPI,VCAM1 | 9 |
| Cell-To-Cell Signaling and Interaction,Reproductive System Development and Function | Binding of gonadal cell lines | 0.00000242 | 5.616184634 |  | 0.132 | APOE,LBP,MMP2,SELP,VCAM1 | 5 |
| Lipid Metabolism,Molecular Transport,Small Molecule Biochemistry | Secretion of lipid | 0.00000253 | 5.596879479 |  | 0.152 | APOA1,APOA4,APOB,APOC3,APOE,NPC2 | 6 |
| Cell-To-Cell Signaling and Interaction | Binding of connective tissue cells | 0.00000362 | 5.441291429 |  | -1.067 | APOE,CCN5,CHAD,FBN1,RNASE1,VCL | 6 |
| Cardiovascular Disease,Organismal Injury and Abnormalities | Stenosis of aortic valve | 0.00000417 | 5.379863945 |  |  | DSP,F10,MMP2,TIMP2,VCAM1 | 5 |
| Cellular Compromise,Inflammatory Response | Degranulation of neutrophils | 0.00000438 | 5.358525889 |  |  | ADA2,C3,CAMP,CHI3L1,CST3,DSP,GM2A,IGF2R,NPC2,PSMB7,TIMP2,VCL | 12 |
| Cell Morphology,Cellular Movement | Cell spreading | 0.00000564 | 5.248720896 |  | 0.127 | ANTXR1,C3,CHAD,FBN1,MYH9,PVR,TFPI,VCAM1 | 8 |
| Cell-To-Cell Signaling and Interaction,Hematological System Development and Function,Immune Cell Trafficking,Inflammatory Response | Activation of phagocytes | 0.00000565 | 5.247951552 |  | -0.674 | ADIPOQ,APOE,C3,CAMP,CD84,LBP,SERPING1 | 7 |
| Connective Tissue Disorders,Inflammatory Disease,Inflammatory Response,Organismal Injury and Abnormalities,Skeletal and Muscular Disorders | Inflammation of joint | 0.00000572 | 5.242603971 |  |  | ADIPOQ,APOA1,APOE,APOM,C3,CHI3L1,CST3,F10,GSR,IGKC,LBP,LCP1,LDHB,MMP2,MRC1,PON1,SAA1,TIMP2,VCAM1,VSIG4 | 20 |
| Cardiovascular System Development and Function,Organismal Development | Angiogenesis | 0.00000579 | 5.237321436 |  | 0.441 | ADIPOQ,APOE,C3,CAMP,CHI3L1,IGFBP4,MCAM,MMP2,MYH9,ROCK2,SAA1,SPINK5,ST6GAL1,TIMP2,VCAM1 | 15 |
| Ophthalmic Disease,Organismal Injury and Abnormalities | Macular degeneration | 0.00000616 | 5.210419288 |  |  | APOE,C3,CST3,EFEMP1,FBLN5,SERPING1 | 6 |
| Lipid Metabolism,Molecular Transport,Small Molecule Biochemistry | Import of cholesterol | 0.00000634 | 5.197910742 |  |  | APOA1,APOC3,NPC2 | 3 |
| Lipid Metabolism,Molecular Transport,Small Molecule Biochemistry | Transport of glycolipid | 0.00000634 | 5.197910742 |  |  | APOA1,NPC2,PLTP | 3 |
| Hematological Disease,Metabolic Disease,Organismal Injury and Abnormalities | Mixed dyslipidemia | 0.00000642 | 5.192464972 |  |  | APOA1,APOB,APOC3,PCSK9 | 4 |
| Cell Death and Survival | Necrosis | 0.0000083 | 5.080921908 |  | -0.188 | ADIPOQ,ANTXR1,APMAP,APOA1,APOB,APOC1,APOC3,APOE,C3,CAMP,CCN5,CSPG4,CST3,DSP,EFEMP1,IGF2R,IGFBP4,IGFBP6,LDHA,LTBP1,MCAM,MMP2,NOTCH3,NPC2,PCSK9,RNASE1,ROCK2,SAA1,ST6GAL1,TAGLN2,VCAM1,VCAN | 32 |
| Organismal Injury and Abnormalities,Renal and Urological Disease | Chronic kidney disease | 0.0000084 | 5.075720714 |  |  | ADIPOQ,APOB,APOE,C3,F10,MYH9,PCSK9,PSMA6,TFPI | 9 |
| Cellular Movement,Hematological System Development and Function,Immune Cell Trafficking | Cell movement of lymphocytes | 0.00000872 | 5.059483515 |  | -0.068 | APOD,CAMP,LCP1,MCAM,MYH9,PPIB,SAA1,SELP | 8 |
| Cell-To-Cell Signaling and Interaction | Aggregation of tumor cell lines | 0.00000888 | 5.051587034 |  | -0.849 | DSC2,MYH9,ROCK2,TIMP2,VCAM1 | 5 |
| Dermatological Diseases and Conditions,Inflammatory Disease,Inflammatory Response,Organismal Injury and Abnormalities | Dermatitis | 0.00000893 | 5.049148541 |  |  | ADIPOQ,C3,CAMP,CST3,DCD,DSP,GSR,MCAM,SELP,SPINK5,VCL | 11 |
| Carbohydrate Metabolism,Lipid Metabolism,Small Molecule Biochemistry | Binding of lipopolysaccharide | 0.00000947 | 5.023650021 |  |  | CAMP,LBP,SELP | 3 |
| Molecular Transport | Quantity of metal | 0.00000971 | 5.01278077 |  | 0.64 | A2M,APOC3,C3,MCAM,PON1,PPIB,SAA1,SELP,VCAM1 | 9 |
| Lipid Metabolism,Small Molecule Biochemistry,Vitamin and Mineral Metabolism | Metabolism of terpenoid | 0.0000103 | 4.987162775 |  |  | APOA1,APOA4,APOB,APOC2,APOC3,APOE,APOM,IGFBP4,NPC2 | 9 |
| Cardiovascular Disease,Organismal Injury and Abnormalities | Peripheral arterial disease | 0.0000106 | 4.974694135 |  |  | ADA2,APOE,F10,LCP1,PCSK9,PLTP,ST6GAL1,TFPI,VSIG4 | 9 |
| Cardiovascular Disease | Vascular lesion | 0.0000111 | 4.954677021 |  |  | APOA1,CST3,FBN1,MMP2,TIMP2,VCAM1,VCAN | 7 |
| Carbohydrate Metabolism | Metabolism of carbohydrate | 0.0000113 | 4.946921557 |  |  | ADIPOQ,ALDOB,APOA1,APOA4,APOD,APOE,CSPG4,EXT1,EXT2,PON1,ST6GAL1,TKT,UGP2,VCAN | 14 |
| Endocrine System Disorders,Gastrointestinal Disease,Metabolic Disease,Organismal Injury and Abnormalities | Diabetic complication | 0.0000118 | 4.928117993 |  |  | APOD,APOE,CHI3L1,COL6A2,CRHBP,F10,FBLN5,LTBP1,MYH9,TFPI,VCAM1 | 11 |
| Cardiovascular Disease,Organismal Injury and Abnormalities | Aortic aneurysm | 0.0000124 | 4.906578315 |  |  | CST3,FBN1,MMP2,TIMP2,VCAN | 5 |
| Cellular Movement,Hematological System Development and Function,Immune Cell Trafficking | Migration of mononuclear leukocytes | 0.0000127 | 4.896196279 |  | -0.192 | APOD,CAMP,LCP1,MCAM,MYH9,SELP,TIMP2,VCAM1 | 8 |
| Ophthalmic Disease,Organismal Injury and Abnormalities | Age-related macular degeneration | 0.0000135 | 4.869666232 |  |  | APOE,C3,CST3,FBLN5,SERPING1 | 5 |
| Carbohydrate Metabolism,Small Molecule Biochemistry | Metabolism of D-hexose | 0.0000137 | 4.863279433 |  |  | ADIPOQ,ALDOB,APOD,APOE | 4 |
| Cardiovascular Disease,Neurological Disease,Organismal Injury and Abnormalities | Stroke | 0.0000143 | 4.844663963 |  |  | ADIPOQ,APOA1,APOB,CST3,F10,LPA,NOTCH3,PCSK9,VCAM1 | 9 |
| Cellular Movement,Hair and Skin Development and Function | Cell movement of epithelial cell lines | 0.0000167 | 4.777283529 |  | 1.954 | ANTXR1,APOA1,C3,CAMP,F10,MCAM,SAA1 | 7 |
| Cellular Movement,Hematological System Development and Function,Immune Cell Trafficking,Inflammatory Response | Cell movement of phagocytes | 0.0000168 | 4.774690718 |  | 0.662 | APOA1,C3,CAMP,LPA,PPIB,SAA1,SELP,TIMP2,VCAM1 | 9 |
| Cancer,Organismal Injury and Abnormalities,Reproductive System Disease | Female genital tract serous carcinoma | 0.0000177 | 4.752026734 |  |  | APOD,APOE,COL6A2,CSPG4,FBLN5,IGFBP4,MCAM,NOTCH3,SAA1,SERPING1,SPARCL1,VCAN | 12 |
| Cancer,Organismal Injury and Abnormalities | Pelvic serous carcinoma | 0.0000177 | 4.752026734 |  |  | APOD,APOE,COL6A2,CSPG4,FBLN5,IGFBP4,MCAM,NOTCH3,SAA1,SERPING1,SPARCL1,VCAN | 12 |
| Organismal Survival | Organismal death | 0.0000215 | 4.66756154 |  |  | CAMP,COL6A2,CST3,DSC2,EFEMP1,LCP1,MCAM,MMP2,PDLIM1,PVR,SAA1,TIMP2,TREML1,VCAN,VCL | 15 |
| Tissue Development | Development of epithelial tissue | 0.0000235 | 4.628932138 |  | -0.18 | ADIPOQ,APOE,C3,CAMP,MCAM,MMP2,SAA1,TIMP2,VCAM1,VCL | 10 |
| Hematological Disease | Factor X deficiency | 0.0000239 | 4.621602099 |  |  | APCS,F10 | 2 |
| Cardiovascular Disease,Hematological Disease,Hereditary Disorder,Metabolic Disease,Organismal Injury and Abnormalities | Familial hypercholesterolemia 3 | 0.0000239 | 4.621602099 |  |  | APOB,PCSK9 | 2 |
| Developmental Disorder,Hematological Disease,Hereditary Disorder,Metabolic Disease,Organismal Injury and Abnormalities | Familial hypobetalipoproteinemia type 1 | 0.0000239 | 4.621602099 |  |  | APOB,PCSK9 | 2 |
| Cancer,Connective Tissue Disorders,Developmental Disorder,Hereditary Disorder,Organismal Injury and Abnormalities,Skeletal and Muscular Disorders | Multiple hereditary exostoses | 0.0000239 | 4.621602099 |  |  | EXT1,EXT2 | 2 |
| Carbohydrate Metabolism,Lipid Metabolism,Small Molecule Biochemistry | Transmission of lipopolysaccharide | 0.0000239 | 4.621602099 |  |  | LBP,PLTP | 2 |
| Lipid Metabolism,Molecular Transport,Small Molecule Biochemistry,Vitamin and Mineral Metabolism | Quantity of vitamin A | 0.0000239 | 4.621602099 |  |  | APOA1,APOC3 | 2 |
| Cardiovascular Disease,Organismal Injury and Abnormalities,Organismal Survival | Aborted sudden cardiac death | 0.0000239 | 4.621602099 |  |  | DSC2,VCL | 2 |
| Cardiovascular System Development and Function,Cellular Movement | Cell movement of endothelial cells | 0.000024 | 4.619788758 |  | 0.404 | ADIPOQ,APOA1,APOE,CAMP,MCAM,MMP2,ROCK2,SAA1,TIMP2,VCAM1 | 10 |
| Cell-To-Cell Signaling and Interaction | Adhesion of connective tissue cells | 0.0000244 | 4.612610174 |  |  | CCN5,CHAD,FBN1,RNASE1,VCL | 5 |
| Cardiovascular Disease,Organismal Injury and Abnormalities | Non-rheumatic aortic stenosis | 0.0000245 | 4.610833916 |  |  | F10,MMP2,VCAM1 | 3 |
| Cardiovascular Disease,Hematological Disease,Hereditary Disorder,Metabolic Disease,Organismal Injury and Abnormalities | Familial combined hyperlipidemia | 0.0000245 | 4.610833916 |  |  | APOA1,APOB,APOC3 | 3 |
| Cardiovascular Disease,Hematological Disease,Metabolic Disease,Organismal Injury and Abnormalities | Hypertriglyceridemia | 0.0000256 | 4.591760035 |  |  | APOA1,APOB,APOC3,APOE | 4 |
| Lipid Metabolism,Molecular Transport,Small Molecule Biochemistry | Concentration of fatty acid | 0.0000262 | 4.581698709 |  |  | ADIPOQ,APOD,LPA,PLTP,PON1 | 5 |
| Cancer,Organismal Injury and Abnormalities,Reproductive System Disease | Breast or ovarian cancer | 0.0000271 | 4.567030709 |  |  | ADA2,ANTXR1,APOA1,APOB,APOD,APOE,C3,CCN5,CHI3L1,COL6A2,CRHBP,CSPG4,DCD,DSP,EFEMP1,EXT1,EXT2,F10,FBLN5,FBN1,GM2A,GSR,IGDCC4,IGF2R,IGFBP4,IGFBP6,LBP,LMAN1,LTBP1,MCAM,MMP2,MRC1,MYH9,NOTCH3,NPC2,OAF,PCSK9,ROCK2,RSU1,SELP,SERPING1,ST6GAL1,SVEP1,TAGLN2,TFPI,TIMP2,TREML1,VCAM1,VCAN | 49 |
| Cancer,Organismal Injury and Abnormalities | Breast or pancreatic cancer | 0.0000278 | 4.555955204 |  |  | ADA2,ADIPOQ,ANTXR1,APOA1,APOB,APOC1,APOC4,APOD,APOE,C3,CCN5,CHI3L1,COL6A2,CRHBP,CSPG4,DCD,DSP,EFEMP1,ERCC6L2,EXT1,EXT2,F10,FBLN5,FBN1,GM2A,GSR,IGDCC4,IGF2R,IGFBP4,IGFBP6,LBP,LMAN1,LPA,LTBP1,MCAM,MMP2,MRC1,MYH9,NOTCH3,NPC2,OAF,PCSK9,PSMA3,ROCK2,RSU1,SAA1,SELP,SERPING1,SPINK5,ST6GAL1,SVEP1,TAGLN2,TFPI,TIMP2,TREML1,UGP2,VCAM1,VCAN,VCL | 59 |
| Cell Signaling,Molecular Transport,Vitamin and Mineral Metabolism | Quantity of Ca2+ | 0.000031 | 4.508638306 |  | 0.64 | A2M,APOC3,C3,MCAM,PPIB,SAA1,SELP,VCAM1 | 8 |
| Cellular Movement,Hematological System Development and Function,Immune Cell Trafficking | Cell movement of granulocytes | 0.0000314 | 4.503070352 |  | 0.6 | APOA1,CAMP,PPIB,ROCK2,SAA1,SELP,VCAM1 | 7 |
| Cancer,Organismal Injury and Abnormalities | Multiple cancers | 0.0000317 | 4.498940738 |  |  | ADA2,ADIPOQ,ANTXR1,APOA1,APOB,APOD,APOE,C3,CCN5,CHI3L1,COL6A2,CRHBP,CSPG4,DCD,DSP,EFEMP1,EXT1,EXT2,F10,FBLN5,FBN1,GM2A,GSR,IGDCC4,IGF2R,IGFBP4,IGFBP6,LBP,LMAN1,LTBP1,MCAM,MMP2,MRC1,MYH9,NOTCH3,NPC2,OAF,PCSK9,ROCK2,RSU1,SELP,SERPING1,ST6GAL1,SVEP1,TAGLN2,TFPI,TIMP2,TREML1,VCAM1,VCAN | 50 |
| Cell-To-Cell Signaling and Interaction | Aggregation of colorectal cancer cell lines | 0.0000318 | 4.49757288 |  |  | DSC2,MYH9,TIMP2 | 3 |
| Cellular Function and Maintenance | Engulfment of cells | 0.0000323 | 4.490797478 |  | 0.463 | ADIPOQ,ANTXR1,APCS,APOA1,APOE,C3,CAMP,CD5L,F10,MRC1 | 10 |
| Lipid Metabolism,Small Molecule Biochemistry | Synthesis of fatty acid | 0.0000328 | 4.484126156 |  | 0.525 | ADIPOQ,APOA4,APOC1,APOC2,APOC3,APOE,CAMP,NPC2 | 8 |
| Cellular Compromise,Inflammatory Response | Degranulation of blood platelets | 0.000034 | 4.468521083 |  |  | A2M,APOA1,SELP,SERPING1,TAGLN2,VCL | 6 |
| Cardiovascular Disease,Organismal Injury and Abnormalities,Skeletal and Muscular Disorders | Acute myocardial infarction | 0.0000355 | 4.449771647 |  |  | ADIPOQ,APOA1,C3,F10,PON1,SELP | 6 |
| Cellular Movement | Migration of colorectal cancer cell lines | 0.0000374 | 4.427128398 |  | 0.798 | CAMP,EFEMP1,IGFBP4,IGFBP6,PDLIM1,SPARCL1,ST6GAL1 | 7 |
| Cardiovascular System Development and Function,Cellular Development,Cellular Function and Maintenance,Cellular Growth and Proliferation,Organismal Development,Tissue Development | Endothelial cell development | 0.0000383 | 4.416801226 |  | -0.083 | ADIPOQ,APOE,C3,CAMP,MCAM,MMP2,SAA1,TIMP2,VCAM1 | 9 |
| Cell-To-Cell Signaling and Interaction | Aggregation of cells | 0.0000387 | 4.412289035 |  | -0.58 | APCS,DSC2,MYH9,ROCK2,SELP,TIMP2,VCAM1 | 7 |
| Cellular Movement,Skeletal and Muscular System Development and Function | Cell movement of smooth muscle cells | 0.0000388 | 4.411168274 |  |  | ADIPOQ,APOE,FBN1,IGFBP4,MMP2 | 5 |
| Cell-To-Cell Signaling and Interaction,Hematological System Development and Function,Immune Cell Trafficking,Inflammatory Response | Binding of macrophages | 0.0000403 | 4.394694954 |  |  | ADIPOQ,APOE,CAMP | 3 |
| Cancer,Organismal Injury and Abnormalities | Benign solid tumor | 0.0000407 | 4.390405591 |  |  | ANTXR1,APCS,APOA1,APOE,CCN5,COL6A2,FBN1,LTBP1,MMP2,MYH9,NOTCH3,PSMA6,SPARCL1,UGP2,VCAM1,VCAN,VSIG4 | 17 |
| Cancer,Hematological Disease,Immunological Disease,Organismal Injury and Abnormalities | Lymphoma | 0.000041 | 4.387216143 |  |  | ANTXR1,C3,CHI3L1,COL6A2,CST3,DCD,DSP,EXT2,F10,FBN1,GSR,IGF2R,IGFBP4,IGKC,MCAM,MMP2,PLTP,ROCK2,SELP,SVEP1,TFPI,TIMP2,VCAM1,VCL | 24 |
| Connective Tissue Disorders,Developmental Disorder,Hereditary Disorder,Organismal Injury and Abnormalities,Skeletal and Muscular Disorders | Marfan syndrome | 0.0000439 | 4.35753548 |  |  | APCS,APOA1,FBN1,VCL | 4 |
| Cardiovascular System Development and Function,Organismal Development | Vasculogenesis | 0.0000454 | 4.342944147 |  | 0.36 | ADIPOQ,APOE,C3,CAMP,CHI3L1,IGFBP4,MCAM,MMP2,SAA1,ST6GAL1,TIMP2,VCAM1 | 12 |
| Molecular Transport | Secretion of molecule | 0.0000457 | 4.3400838 |  | 0.283 | A2M,APOA1,APOA4,APOB,APOC3,APOE,FBLN5,NPC2 | 8 |
| Cellular Assembly and Organization,Neurological Disease,Organismal Development,Tissue Development | Formation of amyloid fibrils | 0.0000502 | 4.299296283 |  |  | A2M,APCS,APOE | 3 |
| Cell Death and Survival | Opsonization | 0.0000502 | 4.299296283 |  |  | ADIPOQ,C3,LBP | 3 |
| Cancer,Gastrointestinal Disease,Hepatic System Disease,Organismal Injury and Abnormalities | Hepatocellular carcinoma | 0.0000509 | 4.293282218 |  |  | ALDOB,APOA1,APOB,C1QTNF3,CCN5,CD5L,COLEC10,CRHBP,F10,FBLN5,IGDCC4,IGF2R,LPA,LTBP1,MMP2,MRC1,PROZ,PVR,SAA4,SERPING1,SVEP1,VCAN,VSIG4 | 23 |
| Cell-To-Cell Signaling and Interaction | Binding of myeloid cells | 0.0000525 | 4.279840697 |  | -0.376 | ADIPOQ,APOE,CAMP,PLTP,SELP,VCAM1 | 6 |
| Organismal Injury and Abnormalities,Reproductive System Disease | Benign pelvic disease | 0.000056 | 4.251811973 |  |  | ADIPOQ,APOE,COL6A2,FBN1,IGHG2,LTBP1,MMP2,PON1,PSMA1,PSMA6,ROCK2,SPARCL1,TIMP2,VCAN | 14 |
| Cell-To-Cell Signaling and Interaction,Hematological System Development and Function,Hematopoiesis | Adhesion of red blood cells | 0.0000616 | 4.210419288 |  |  | APCS,SELP,SERPING1 | 3 |
| Cardiovascular Disease,Neurological Disease,Organismal Injury and Abnormalities | Infarction of cerebrum | 0.0000624 | 4.20481541 |  |  | APOA1,APOB,F10,LPA,NOTCH3 | 5 |
| Cancer,Organismal Injury and Abnormalities,Reproductive System Disease | Mammary tumor | 0.0000638 | 4.195179321 |  |  | ADA2,APOA1,APOB,APOD,APOE,C3,CAMP,CHI3L1,CSPG4,DCD,DSP,EFEMP1,EXT2,F10,FBLN5,FBN1,GSR,IGDCC4,IGF2R,IGFBP6,LBP,LMAN1,LTBP1,MCAM,MMP2,MRC1,MYH9,NOTCH3,NPC2,OAF,ROCK2,SELP,SERPING1,SVEP1,TAGLN2,TFPI,TIMP2,TREML1,VCAN | 39 |
| Cardiovascular System Development and Function,Cellular Movement | Migration of endothelial cells | 0.0000642 | 4.192464972 |  | 0.127 | ADIPOQ,APOA1,APOE,MCAM,MMP2,ROCK2,SAA1,TIMP2,VCAM1 | 9 |
| Cardiovascular Disease,Hematological Disease,Organismal Injury and Abnormalities | Thrombus | 0.0000644 | 4.191114133 |  |  | C3,F10,MMP2,PON1,SELP,TFPI | 6 |
| Dermatological Diseases and Conditions,Organismal Injury and Abnormalities | Abnormality of skin morphology | 0.0000682 | 4.166215625 |  |  | APOE,C3,DCD,DSC2,DSP,FBN1,IGFBP6,LCP1,PGD,TKT | 10 |
| Cellular Development,Cellular Growth and Proliferation,Organ Development,Skeletal and Muscular System Development and Function,Tissue Development | Proliferation of smooth muscle cells | 0.0000697 | 4.156767222 |  | -0.726 | ADIPOQ,APOD,APOE,CCN5,LPA,NOTCH3 | 6 |
| Lipid Metabolism,Molecular Transport,Small Molecule Biochemistry | Release of lipid | 0.0000697 | 4.156767222 |  | 1.17 | ADIPOQ,APOA1,APOD,C3,CAMP | 5 |
| Cell-To-Cell Signaling and Interaction | Binding of melanoma cell lines | 0.0000702 | 4.153662888 |  |  | MCAM,ROCK2,SELP,VCAN | 4 |
| Cardiovascular Disease,Hematological Disease,Organismal Injury and Abnormalities | Formation of thrombus | 0.0000715 | 4.145693958 |  |  | C3,SELP | 2 |
| Drug Metabolism,Lipid Metabolism,Molecular Transport,Small Molecule Biochemistry | Release of prostaglandin E2 | 0.0000745 | 4.127843727 |  |  | ADIPOQ,C3,CAMP | 3 |
| Cardiovascular Disease,Organismal Injury and Abnormalities | Arrhythmia | 0.0000747 | 4.126679398 |  |  | DSC2,DSP,F10,FBN1,MMP2,TFPI,VCAM1,VCL | 8 |
| Cellular Movement,Renal and Urological System Development and Function | Cell movement of kidney cell lines | 0.0000754 | 4.122628654 |  |  | ANTXR1,APOA1,C3,CAMP,F10,SAA1 | 6 |
| Organismal Injury and Abnormalities,Renal and Urological Disease | Chronic renal failure | 0.0000814 | 4.089375595 |  |  | APOB,C3,F10,MYH9,PCSK9,TFPI | 6 |
| Molecular Transport | Transport of molecule | 0.0000828 | 4.081969663 |  | 1.766 | A2M,ADIPOQ,APOA1,APOA4,APOB,APOC1,APOC2,APOC3,APOE,APOM,C3,CAMP,FBLN5,LBP,MYH9,NPC2,PLTP,PON1,SAA1 | 19 |
| Cell-To-Cell Signaling and Interaction,Embryonic Development | Adhesion of embryonic cells | 0.0000834 | 4.078833949 |  |  | ANTXR1,C3,F10,VCAM1 | 4 |
| Cancer,Organismal Injury and Abnormalities,Reproductive System Disease | Breast cancer | 0.0000852 | 4.069560405 |  |  | ADA2,APOA1,APOB,APOD,APOE,C3,CHI3L1,CSPG4,DCD,DSP,EFEMP1,EXT2,F10,FBLN5,FBN1,GSR,IGDCC4,IGF2R,IGFBP6,LBP,LMAN1,LTBP1,MCAM,MMP2,MRC1,MYH9,NOTCH3,NPC2,OAF,ROCK2,SELP,SERPING1,SVEP1,TAGLN2,TFPI,TIMP2,TREML1,VCAN | 38 |
| Cellular Assembly and Organization,Tissue Development | Formation of fibrils | 0.0000891 | 4.050122296 |  |  | A2M,APCS,APOE | 3 |
| Organismal Development,Tissue Development | Morphogenesis of epithelial tissue | 0.0000906 | 4.042871802 |  |  | MMP2,TIMP2,VCAM1,VCL | 4 |
| Inflammatory Response | Inflammation of absolute anatomical region | 0.0000958 | 4.018634491 |  |  | ADIPOQ,APOA1,APOB,APOE,C3,CAMP,CST3,F10,IGHG4,LTBP1,MYH9,PDLIM1,SELP,VCAM1 | 14 |
| Connective Tissue Development and Function,Tissue Morphology | Quantity of connective tissue | 0.0000958 | 4.018634491 |  |  | ADIPOQ,APOA4,APOB,APOE,SAA1 | 5 |
| Cancer,Organismal Injury and Abnormalities | Connective or soft tissue tumor | 0.0000964 | 4.015922966 |  |  | APOA4,APOB,CAMP,CCN5,COL6A2,CSPG4,EXT1,EXT2,F10,FBN1,GSR,IGF2R,LTBP1,MCAM,MMP2,MYH9,NOTCH3,PCSK9,PSMA6,ROCK2,RSU1,SPARCL1,TKT,VCAN,VCL | 25 |
| Cellular Movement | Cell movement of hepatoma cell lines | 0.000106 | 3.974694135 |  | 0.314 | ADIPOQ,RSU1,ST6GAL1,TAGLN2,VCAM1,VCAN | 6 |
| Connective Tissue Development and Function,Tissue Morphology | Quantity of adipose tissue | 0.000106 | 3.974694135 |  |  | ADIPOQ,APOA4,APOB,APOE | 4 |
| Cancer,Hematological Disease,Organismal Injury and Abnormalities | Mature lymphocytic neoplasm | 0.000113 | 3.946921557 |  |  | ADIPOQ,ANTXR1,APOE,C3,COL6A2,CST3,DCD,DSP,EXT2,F10,FBN1,GSR,IGF2R,IGFBP4,IGKC,MCAM,MMP2,PCSK9,PLTP,ROCK2,SELP,SVEP1,TFPI,TIMP2,VCAM1,VCAN,VCL | 27 |
| Immunological Disease | Hypersensitive reaction | 0.000119 | 3.924453039 |  |  | ADIPOQ,C3,CAMP,CST3,DCD,MCAM,SELP,SPINK5,VCL | 9 |
| Cellular Movement | Migration of myeloid cells | 0.000123 | 3.910094889 |  | -1.067 | CAMP,ROCK2,SAA1,TIMP2,VCAM1 | 5 |
| Cardiovascular Disease,Organismal Injury and Abnormalities | Atherosclerotic lesion | 0.000124 | 3.906578315 |  |  | APOA1,VCAM1,VCAN | 3 |
| Lipid Metabolism,Molecular Transport,Small Molecule Biochemistry | Concentration of sterol | 0.000124 | 3.906578315 |  |  | ADIPOQ,APOA1,APOE,NPC2 | 4 |
| Cellular Movement | Invasion of cells | 0.000126 | 3.899629455 | Increased | 2.035 | ADIPOQ,CHI3L1,CSPG4,DSP,EFEMP1,FBLN5,LCP1,MCAM,MMP2,PDLIM1,SAA1,SPARCL1,ST6GAL1,TAGLN2,TFPI,TIMP2,VCAM1,VCAN | 18 |
| Cancer,Hematological Disease,Immunological Disease,Organismal Injury and Abnormalities | Lymphocytic cancer | 0.000128 | 3.89279003 |  |  | ADIPOQ,ANTXR1,APOE,C3,CHI3L1,COL6A2,CSPG4,CST3,DCD,DSP,EXT2,F10,FBN1,GSR,IGF2R,IGFBP4,IGKC,LDHA,LPA,LTBP1,MCAM,MMP2,NOTCH3,PCSK9,PLTP,ROCK2,SELP,SVEP1,TFPI,TIMP2,VCAM1,VCAN,VCL | 33 |
| Cancer,Hematological Disease,Organismal Injury and Abnormalities | Lymphocytic neoplasm | 0.00013 | 3.886056648 |  |  | ADIPOQ,ANTXR1,APOE,C3,CHI3L1,COL6A2,CSPG4,CST3,DCD,DSP,EXT2,F10,FBN1,GSR,IGF2R,IGFBP4,IGKC,LDHA,LPA,LTBP1,MCAM,MMP2,NOTCH3,PCSK9,PLTP,ROCK2,SELP,SVEP1,TFPI,TIMP2,VCAM1,VCAN,VCL | 33 |
| Cellular Movement | Invasion of tumor cell lines | 0.000136 | 3.866461092 |  | 1.683 | ADIPOQ,CSPG4,DSP,EFEMP1,FBLN5,LCP1,MCAM,MMP2,PDLIM1,SAA1,SPARCL1,ST6GAL1,TAGLN2,TFPI,TIMP2,VCAM1,VCAN | 17 |
| Cardiovascular Disease | Hypertension | 0.000137 | 3.863279433 |  |  | ADIPOQ,APOB,C3,CCN5,CHI3L1,CST3,DSC2,F10,GSR,NOTCH3,SELP,SPARCL1,TAGLN2,VCAM1,VCL | 15 |
| Cellular Movement,Embryonic Development | Migration of embryonic cell lines | 0.000142 | 3.847711656 |  |  | ADIPOQ,C3,F10,ROCK2,SAA1 | 5 |
| Cardiovascular Disease,Hematological Disease,Organismal Injury and Abnormalities | Symptomatic venous thromboembolism | 0.000143 | 3.844663963 |  |  | F10,TFPI | 2 |
| Cardiovascular Disease,Hematological Disease,Organismal Injury and Abnormalities | Mesenteric thrombosis | 0.000143 | 3.844663963 |  |  | F10,TFPI | 2 |
| Cardiovascular Disease,Organismal Injury and Abnormalities | Atherogenesis | 0.000143 | 3.844663963 |  |  | APOA1,VCAM1 | 2 |
| Cardiovascular Disease,Hematological Disease,Organismal Injury and Abnormalities,Renal and Urological Disease | Renal vein thrombosis | 0.000143 | 3.844663963 |  |  | F10,TFPI | 2 |
| Cancer,Cardiovascular Disease,Organismal Injury and Abnormalities | Susceptibility to capillary infantile hemangioma | 0.000143 | 3.844663963 |  |  | ANTXR1,MYH9 | 2 |
| Cancer,Organismal Injury and Abnormalities | Nonmetastatic transitional-cell carcinoma | 0.000143 | 3.844663963 |  |  | MMP2,TIMP2 | 2 |
| Cell-To-Cell Signaling and Interaction | Adhesion of Candida albicans | 0.000143 | 3.844663963 |  |  | C3,CAMP | 2 |
| Cancer,Organismal Injury and Abnormalities,Renal and Urological Disease | High grade renal cell carcinoma | 0.000143 | 3.844663963 |  |  | MMP2,TIMP2 | 2 |
| Carbohydrate Metabolism,Small Molecule Biochemistry | Length of heparan sulfate | 0.000143 | 3.844663963 |  |  | EXT1,EXT2 | 2 |
| Lipid Metabolism,Molecular Transport,Small Molecule Biochemistry | Quantity of non-esterified fatty acid | 0.000143 | 3.844663963 |  |  | LPA,PLTP | 2 |
| Cellular Movement,Hematological System Development and Function,Immune Cell Trafficking,Inflammatory Response | Chemotaxis of mononuclear leukocytes | 0.000145 | 3.838631998 |  | 1.481 | APOA1,CAMP,LCP1,LPA,PPIB,SAA1 | 6 |
| Lipid Metabolism,Small Molecule Biochemistry | Synthesis of lipid | 0.000145 | 3.838631998 |  | 0.558 | A2M,ADIPOQ,APOA1,APOA4,APOC1,APOC2,APOC3,APOE,C3,CAMP,IGFBP4,NPC2,PGD | 13 |
| Endocrine System Disorders,Gastrointestinal Disease,Metabolic Disease,Organismal Injury and Abnormalities | Non-insulin-dependent diabetes mellitus | 0.000146 | 3.835647144 |  |  | ADIPOQ,APOB,APOC1,APOC3,PCSK9,PSMA1,PSMA3,PSMB7,SELP,VCAM1 | 10 |
| Carbohydrate Metabolism | Quantity of carbohydrate | 0.00015 | 3.823908741 |  | 0.771 | ADIPOQ,APOE,LPA,PON1,ROCK2,UGP2 | 6 |
| Dermatological Diseases and Conditions,Immunological Disease,Inflammatory Disease,Inflammatory Response,Organismal Injury and Abnormalities | Atopic dermatitis | 0.000164 | 3.785156152 |  |  | ADIPOQ,C3,CAMP,CST3,DCD,SELP,SPINK5,VCL | 8 |
| Cardiovascular Disease,Organismal Injury and Abnormalities | Advanced stage peripheral arterial disease | 0.000165 | 3.782516056 |  |  | ADA2,APOE,F10,LCP1,ST6GAL1,VSIG4 | 6 |
| Cell-To-Cell Signaling and Interaction,Cellular Assembly and Organization,Hematological System Development and Function,Immune Cell Trafficking | Cell-cell adhesion of leukocytes | 0.000216 | 3.665546249 |  |  | APOA4,SELP,VCAM1 | 3 |
| Infectious Diseases | Severe sepsis | 0.000217 | 3.663540266 |  |  | APOC1,APOE,C3,F10 | 4 |
| Cardiovascular Disease,Organismal Injury and Abnormalities | Intermediate disease stage peripheral arterial disease | 0.000221 | 3.655607726 |  |  | ADA2,APOE,LCP1,PLTP,ST6GAL1,VSIG4 | 6 |
| Carbohydrate Metabolism | Synthesis of polysaccharide | 0.000228 | 3.642065153 |  |  | APOE,CSPG4,EXT1,EXT2,UGP2,VCAN | 6 |
| Inflammatory Response | Inflammation of body cavity | 0.000234 | 3.630784143 |  |  | ADIPOQ,APOA1,APOB,APOE,C3,CAMP,F10,IGHG4,LTBP1,MYH9,PDLIM1,SELP,VCAM1 | 13 |
| Cardiovascular Disease,Hematological Disease,Organismal Injury and Abnormalities | Acute venous thromboembolism | 0.000237 | 3.625251654 |  |  | F10,TFPI | 2 |
| Carbohydrate Metabolism,Small Molecule Biochemistry | 6-O-sulfation of heparan sulfate | 0.000237 | 3.625251654 |  |  | EXT1,EXT2 | 2 |
| Cardiovascular System Development and Function,Tissue Development | Adhesion of endothelial tissue | 0.000237 | 3.625251654 |  |  | C3,F10 | 2 |
| Cellular Assembly and Organization | Rearrangement of actin filaments | 0.000237 | 3.625251654 |  |  | MYH9,VCAM1 | 2 |
| Cancer,Organismal Injury and Abnormalities | Advanced malignant tumor | 0.00025 | 3.602059991 |  |  | APOE,C3,EFEMP1,EXT1,F10,GSR,IGFBP4,MCAM,MMP2,MYH9,NOTCH3,RNASE1,SERPING1,TFPI,VCAM1,VCAN | 16 |
| Cell-To-Cell Signaling and Interaction,Cellular Function and Maintenance,Inflammatory Response | Phagocytosis of cells | 0.00026 | 3.585026652 |  | -0.654 | ADIPOQ,APCS,APOA1,C3,CAMP,CD5L,F10 | 7 |
| Infectious Diseases | Infection of cells | 0.000261 | 3.583359493 |  | 0.768 | APCS,APOE,C3,CCN5,DCD,F10,GM2A,IGF2R,MRC1,PPIB,PSMA1,PSMA3,PVR,ST6GAL1,TAGLN2 | 15 |
| Organismal Injury and Abnormalities,Reproductive System Disease | Endometriosis | 0.000266 | 3.575118363 |  |  | ADIPOQ,APOE,COL6A2,FBN1,IGHG2,MMP2,PON1,PSMA1,ROCK2,TIMP2 | 10 |
| Immunological Disease | Allergy | 0.000266 | 3.575118363 |  |  | ADIPOQ,C3,CAMP,CST3,DCD,SELP,SERPING1,SPINK5,VCL | 9 |
| Cardiovascular System Development and Function,Cell Morphology,Cellular Development,Cellular Function and Maintenance,Cellular Growth and Proliferation,Organismal Development,Tissue Development | Morphogenesis of endothelial cells | 0.000276 | 3.559090918 |  |  | MMP2,TIMP2,VCAM1 | 3 |
| Cellular Movement | Migration of sarcoma cell lines | 0.000282 | 3.549750892 |  | 1.22 | FBLN5,IGFBP6,MCAM,TIMP2,VCAN | 5 |
| Cellular Movement,Hematological System Development and Function,Immune Cell Trafficking | Lymphocyte migration | 0.000289 | 3.539102157 |  | 0.372 | APOD,CAMP,LCP1,MCAM,MYH9,SELP | 6 |
| Cancer,Hematological Disease,Immunological Disease,Organismal Injury and Abnormalities | Non-Hodgkin lymphoma | 0.000305 | 3.515700161 |  |  | ANTXR1,C3,COL6A2,CST3,DCD,DSP,EXT2,FBN1,GSR,IGF2R,IGFBP4,IGKC,MCAM,MMP2,PLTP,ROCK2,SELP,SVEP1,TIMP2,VCAM1,VCL | 21 |
| Hematological System Development and Function,Organismal Functions | Coagulation of blood | 0.000307 | 3.512861625 |  | -1.969 | A2M,APOE,F10,LMAN1,SELP,SERPING1,TFPI | 7 |
| Carbohydrate Metabolism,Small Molecule Biochemistry | Metabolism of D-glucose | 0.000309 | 3.510041521 |  |  | ADIPOQ,APOD,APOE | 3 |
| Tissue Development | Disassembly of extracellular matrix | 0.000332 | 3.478861916 |  |  | A2M,LCP1,MMP2,TIMP2 | 4 |
| Lipid Metabolism,Molecular Transport,Small Molecule Biochemistry | Release of eicosanoid | 0.000332 | 3.478861916 |  | 0.719 | ADIPOQ,APOD,C3,CAMP | 4 |
| Connective Tissue Disorders,Immunological Disease,Organismal Injury and Abnormalities | Collagen disease | 0.000342 | 3.465973894 |  |  | ADIPOQ,C3,COL6A2,F10,MYH9 | 5 |
| Cardiovascular Disease,Cardiovascular System Development and Function,Hereditary Disorder,Organ Morphology,Organismal Development,Organismal Injury and Abnormalities,Skeletal and Muscular Disorders | Dilated cardiomyopathy type 1B | 0.000345 | 3.462180905 |  |  | DSC2,DSP,VCL | 3 |
| Cellular Function and Maintenance | Engulfment of bacteria | 0.000345 | 3.462180905 |  |  | C3,CAMP,MRC1 | 3 |
| Hereditary Disorder,Neurological Disease,Organismal Injury and Abnormalities,Psychological Disorders,Skeletal and Muscular Disorders | Huntington Disease | 0.000348 | 3.458420756 |  |  | A2M,APOA4,APOE,C3,CHI3L1,LDHA,LDHB,PDLIM1,PON1,ROCK2,VCAN | 11 |
| Developmental Disorder,Hereditary Disorder,Metabolic Disease,Organismal Injury and Abnormalities | Hereditary systemic amyloidosis | 0.000354 | 3.450996738 |  |  | APOA1,APOE | 2 |
| Cardiovascular Disease,Hematological Disease,Neurological Disease,Organismal Injury and Abnormalities | Cerebral venous thrombosis | 0.000354 | 3.450996738 |  |  | F10,TFPI | 2 |
| Cardiovascular Disease,Hereditary Disorder,Organismal Injury and Abnormalities,Skeletal and Muscular Disorders | Nonsyndromic arrhythmogenic right ventricular cardiomyopathy | 0.000354 | 3.450996738 |  |  | DSC2,DSP | 2 |
| Cellular Movement,Hematological System Development and Function,Immune Cell Trafficking,Inflammatory Response | Chemotaxis of leukocytes | 0.00036 | 3.443697499 |  | 1.167 | APOA1,C3,CAMP,LCP1,LPA,PPIB,SAA1 | 7 |
| Cell Morphology,Cellular Movement | Cell spreading of tumor cell lines | 0.000368 | 3.434152181 |  | -0.6 | C3,MYH9,PVR,TFPI,VCAM1 | 5 |
| Cellular Function and Maintenance | Engulfment of phagocytes | 0.000372 | 3.42945706 |  | -0.038 | APCS,C3,CAMP,MRC1 | 4 |
| Cellular Compromise,Hypersensitivity Response,Inflammatory Response | Degranulation of mast cells | 0.000384 | 3.415668776 |  |  | C3,CAMP,CD84 | 3 |
| Carbohydrate Metabolism,Small Molecule Biochemistry | Synthesis of proteoglycan | 0.000384 | 3.415668776 |  |  | APOE,EXT1,EXT2 | 3 |
| Cellular Movement | Transmigration of cells | 0.000396 | 3.402304814 |  | -0.152 | MCAM,MMP2,MYH9,TIMP2,VCAM1 | 5 |
| Cancer,Hematological Disease,Immunological Disease,Organismal Injury and Abnormalities | Neoplasia of leukocytes | 0.000404 | 3.393618635 |  |  | ADIPOQ,ANTXR1,APOE,C3,CHI3L1,COL6A2,CST3,DCD,DSP,EXT2,F10,FBN1,GSR,IGF2R,IGFBP4,IGKC,LDHA,LPA,LTBP1,MCAM,MMP2,PCSK9,PLTP,ROCK2,SELP,SVEP1,TFPI,TIMP2,VCAM1,VCAN,VCL | 31 |
| Cardiovascular Disease,Organismal Injury and Abnormalities | Abdominal aortic aneurysm | 0.000469 | 3.328827157 |  |  | CST3,MMP2,TIMP2 | 3 |
| Cellular Movement | Chemotaxis of myeloid cells | 0.00049 | 3.30980392 |  | 1.287 | APOA1,C3,CAMP,LPA,PPIB,SAA1 | 6 |
| Cardiovascular Disease,Hematological Disease,Metabolic Disease,Organismal Injury and Abnormalities | Severe hyperlipidemia | 0.000494 | 3.306273051 |  |  | APOC3,PCSK9 | 2 |
| Organismal Injury and Abnormalities,Respiratory Disease | Acute chest syndrome | 0.000494 | 3.306273051 |  |  | F10,TFPI | 2 |
| Cell Death and Survival | Opsonization of cells | 0.000494 | 3.306273051 |  |  | ADIPOQ,C3 | 2 |
| Cell-mediated Immune Response,Cellular Movement,Hematological System Development and Function,Immune Cell Trafficking | T cell migration | 0.000508 | 3.294136288 |  | -0.083 | APOD,CAMP,LCP1,MYH9,SELP | 5 |
| Cellular Movement,Hematological System Development and Function,Immune Cell Trafficking,Inflammatory Response | Cell movement of monocytes | 0.000508 | 3.294136288 |  | 1.014 | APOA1,CAMP,LPA,TIMP2,VCAM1 | 5 |
| Organismal Injury and Abnormalities,Reproductive System Disease | Disorder of pregnancy | 0.00051 | 3.292429824 |  |  | C3,CCN5,CHI3L1,DSC2,F10,GSR,IGFBP6,NOTCH3,SELP,SPARCL1,TAGLN2,TFPI,VCL | 13 |
| Cell-To-Cell Signaling and Interaction,Tissue Development | Adhesion of extracellular matrix | 0.000511 | 3.2915791 |  |  | FBLN5,VCAM1,VCAN,VCL | 4 |
| Cancer,Hematological Disease,Organismal Injury and Abnormalities | Hematologic cancer of cells | 0.000524 | 3.280668713 |  |  | ANTXR1,C3,CHI3L1,COL6A2,CST3,DCD,DSP,EXT2,F10,FBN1,GSR,IGF2R,IGFBP4,IGKC,LDHA,LPA,LTBP1,MCAM,MMP2,PCSK9,PLTP,ROCK2,SELP,SVEP1,TFPI,TIMP2,VCAM1,VCAN,VCL | 29 |
| Cellular Movement | Chemotaxis | 0.000525 | 3.279840697 |  | 0.658 | APOA1,C3,CAMP,F10,LCP1,LPA,MMP2,PPIB,SAA1 | 9 |
| Cellular Movement,Hematological System Development and Function,Immune Cell Trafficking,Inflammatory Response | Chemotaxis of phagocytes | 0.000529 | 3.276544328 |  | 1.286 | APOA1,C3,CAMP,LPA,PPIB,SAA1 | 6 |
| Cancer,Hematological Disease,Immunological Disease,Organismal Injury and Abnormalities | T-cell malignant neoplasm | 0.000545 | 3.263603498 |  |  | C3,COL6A2,CSPG4,FBN1,GSR,IGFBP4,IGKC,MCAM,MMP2,NOTCH3,SELP,SVEP1,TIMP2,VCAM1 | 14 |
| Cellular Function and Maintenance | Internalization of bacteria | 0.000565 | 3.247951552 |  |  | C3,CAMP,MRC1 | 3 |
| Infectious Diseases | Sepsis | 0.000571 | 3.243363892 |  |  | APOC1,APOE,C3,F10,MMP2,SERPING1 | 6 |
| DNA Replication, Recombination, and Repair | Synthesis of DNA | 0.000577 | 3.238824187 |  | -1.123 | ADIPOQ,APOE,EFEMP1,F10,FBLN5,IGFBP4,TIMP2 | 7 |
| Cellular Movement | Migration of hepatoma cell lines | 0.000601 | 3.221125528 |  | -0.218 | ADIPOQ,RSU1,ST6GAL1,VCAM1,VCAN | 5 |
| Cancer,Hematological Disease,Immunological Disease,Organismal Injury and Abnormalities | B cell cancer | 0.000635 | 3.197226275 |  |  | ADIPOQ,ANTXR1,APOE,C3,CHI3L1,CST3,DCD,DSP,EXT2,F10,FBN1,GSR,IGF2R,IGKC,LPA,MCAM,MMP2,PLTP,ROCK2,SELP,SVEP1,TFPI,TIMP2,VCAN,VCL | 25 |
| Cellular Movement,Hematological System Development and Function,Immune Cell Trafficking,Inflammatory Response | Cell movement of neutrophils | 0.000641 | 3.19314197 |  | 1.078 | APOA1,CAMP,PPIB,SAA1,SELP | 5 |
| Cardiovascular Disease,Gastrointestinal Disease,Hematological Disease,Hepatic System Disease,Organismal Injury and Abnormalities | Portal vein thrombosis | 0.000657 | 3.18243463 |  |  | F10,TFPI | 2 |
| Lipid Metabolism,Molecular Transport,Small Molecule Biochemistry | Accumulation of ganglioside GM2 | 0.000657 | 3.18243463 |  |  | GM2A,NPC2 | 2 |
| Cardiovascular Disease,Organismal Injury and Abnormalities | Intracranial atherosclerosis | 0.000657 | 3.18243463 |  |  | F10,LPA | 2 |
| Cardiovascular Disease,Hematological Disease,Organismal Injury and Abnormalities | Thrombosis of artery | 0.000657 | 3.18243463 |  |  | F10,TFPI | 2 |
| Cardiovascular Disease | Ischemic complication | 0.000657 | 3.18243463 |  |  | F10,TFPI | 2 |
| Cardiovascular Disease,Organismal Injury and Abnormalities,Reproductive System Disease | Preeclampsia | 0.000662 | 3.179142011 |  |  | C3,CCN5,CHI3L1,DSC2,F10,GSR,NOTCH3,SELP,SPARCL1,TAGLN2,VCL | 11 |
| Cell Death and Survival | Cytolysis | 0.000663 | 3.178486472 |  | 1.27 | C3,CAMP,CCN5,GSR,LBP,PVR | 6 |
| Cell-To-Cell Signaling and Interaction,Hematological System Development and Function,Inflammatory Response | Immune response of neutrophils | 0.000673 | 3.171984936 |  |  | APCS,C3,CAMP | 3 |
| Cell-To-Cell Signaling and Interaction | Adhesion of melanoma cell lines | 0.000673 | 3.171984936 |  |  | MCAM,ROCK2,VCAN | 3 |
| Hereditary Disorder,Organismal Injury and Abnormalities | SCN5A-related disorder | 0.000673 | 3.171984936 |  |  | DSP,FBN1,VCL | 3 |
| Cell-To-Cell Signaling and Interaction | Interaction of cancer cells | 0.000673 | 3.171984936 |  |  | MCAM,SELP,VCAM1 | 3 |
| Cardiovascular System Development and Function,Cellular Development,Cellular Function and Maintenance,Cellular Growth and Proliferation,Organismal Development,Tissue Development | Proliferation of endothelial cells | 0.000677 | 3.169411331 |  | -0.092 | ADIPOQ,APOE,C3,CAMP,MCAM,SAA1,TIMP2 | 7 |
| Cancer,Hematological Disease,Immunological Disease,Organismal Injury and Abnormalities | T-cell non-Hodgkin lymphoma | 0.000702 | 3.153662888 |  |  | C3,COL6A2,FBN1,GSR,IGFBP4,IGKC,MCAM,MMP2,SELP,TIMP2,VCAM1 | 11 |
| Organismal Injury and Abnormalities,Renal and Urological Disease | Nephrosis | 0.000706 | 3.151195299 |  |  | C3,CST3,F10,MYH9,PDLIM1 | 5 |
| Respiratory Disease | Lower respiratory tract disorder | 0.000706 | 3.151195299 |  |  | APOB,CST3,DSP,LBP,LCP1,LDHB,PLTP,PON1 | 8 |
| Gastrointestinal Disease,Hepatic System Disease | Cholestasis | 0.000717 | 3.144480844 |  |  | APOA1,LBP,LPA,UGP2 | 4 |
| Hematological Disease | Coagulation factor deficiency syndrome | 0.000731 | 3.136082623 |  |  | APCS,F10,LMAN1 | 3 |
| Cell-To-Cell Signaling and Interaction,Connective Tissue Development and Function | Binding of fibroblasts | 0.000731 | 3.136082623 |  |  | APOE,RNASE1,VCL | 3 |
| Cardiovascular System Development and Function,Cell-To-Cell Signaling and Interaction | Adhesion of endothelial cells | 0.000751 | 3.124360063 |  | 0.479 | F10,MCAM,SELP,SERPING1,VCAM1 | 5 |
| Cell-To-Cell Signaling and Interaction | Activation of cells | 0.000832 | 3.079876674 |  | -0.774 | ADIPOQ,APOE,C3,CAMP,CD84,LBP,MMP2,NPC2,SELP,SERPING1 | 10 |
| Cancer,Endocrine System Disorders,Organismal Injury and Abnormalities,Reproductive System Disease | Serous ovarian adenocarcinoma | 0.000837 | 3.077274542 |  |  | APOE,COL6A2,IGFBP4,MCAM,NOTCH3,SERPING1,VCAN | 7 |
| Cell Morphology,Cellular Movement | Cell spreading of connective tissue cells | 0.000842 | 3.074687909 |  |  | CHAD,FBN1 | 2 |
| Cardiovascular Disease,Hereditary Disorder,Organismal Injury and Abnormalities,Skeletal and Muscular Disorders | Familial arrhythmogenic right ventricular dysplasia type 1 | 0.000842 | 3.074687909 |  |  | DSC2,DSP | 2 |
| Cellular Function and Maintenance,Molecular Transport | Regulated secretory pathway | 0.000842 | 3.074687909 |  |  | CRHBP,MYH9 | 2 |
| Hematological Disease | Hypercoagulation | 0.000842 | 3.074687909 |  |  | F10,TFPI | 2 |
| Cell-To-Cell Signaling and Interaction | Activation of epithelial cells | 0.000842 | 3.074687909 |  |  | CAMP,NPC2 | 2 |
| Cell-To-Cell Signaling and Interaction,Hematological System Development and Function | Interaction of granulocytes | 0.000857 | 3.067019178 |  | -0.293 | PLTP,SAA1,SELP,VCAM1 | 4 |
| Hereditary Disorder,Immunological Disease,Organismal Injury and Abnormalities | Familial autoimmune disease | 0.000861 | 3.064996849 |  |  | ADA2,ADIPOQ,C3,COL6A2,F10,MYH9 | 6 |
| Cancer,Endocrine System Disorders,Organismal Injury and Abnormalities,Reproductive System Disease | Gonadal tumor | 0.000866 | 3.062482108 |  |  | ANTXR1,APOA1,APOB,APOE,C3,CAMP,CCN5,CHI3L1,COL6A2,CRHBP,CSPG4,EXT1,GM2A,IGDCC4,IGF2R,IGFBP4,MCAM,MMP2,MYH9,NOTCH3,PCSK9,RSU1,SELP,SERPING1,ST6GAL1,VCAM1,VCAN | 27 |
| Cell-To-Cell Signaling and Interaction | Adhesion of epithelial cells | 0.000895 | 3.048176965 |  |  | ANTXR1,C3,F10,ROCK2 | 4 |
| Cancer,Organismal Injury and Abnormalities | Subcutaneous tumor | 0.000898 | 3.046723663 |  |  | CCN5,COL6A2,FBN1,LTBP1,MMP2,NOTCH3,PSMA6,SPARCL1,VCAN | 9 |
| Cancer,Hematological Disease,Immunological Disease,Organismal Injury and Abnormalities | Tumorigenesis of lymphocytes | 0.000904 | 3.04383157 |  |  | ADIPOQ,ANTXR1,APOE,C3,CHI3L1,COL6A2,CST3,DCD,DSP,EXT2,F10,FBN1,GSR,IGF2R,IGFBP4,IGKC,LPA,MCAM,MMP2,PCSK9,PLTP,ROCK2,SELP,SVEP1,TFPI,TIMP2,VCAM1,VCAN,VCL | 29 |
| Cancer,Organismal Injury and Abnormalities | Benign connective or soft tissue neoplasm | 0.000911 | 3.040481623 |  |  | CCN5,COL6A2,FBN1,LTBP1,MMP2,NOTCH3,PSMA6,SPARCL1,VCAN | 9 |
| Cancer,Hematological Disease,Immunological Disease,Organismal Injury and Abnormalities | Mature B cell malignant tumor | 0.00092 | 3.036212173 |  |  | ADIPOQ,ANTXR1,APOE,CST3,DCD,DSP,EXT2,F10,FBN1,GSR,IGF2R,IGKC,MMP2,PLTP,ROCK2,SVEP1,TFPI,TIMP2,VCL | 19 |
| Endocrine System Disorders,Organismal Injury and Abnormalities | Benign thyroid nodule | 0.000926 | 3.033389013 |  |  | APCS,APOA1,PSMA3 | 3 |
| Cancer,Endocrine System Disorders,Organismal Injury and Abnormalities,Reproductive System Disease | Ovarian tumor | 0.000952 | 3.021363052 |  |  | ANTXR1,APOA1,APOB,APOE,C3,CAMP,CCN5,CHI3L1,COL6A2,CRHBP,CSPG4,EXT1,GM2A,IGF2R,IGFBP4,MCAM,MMP2,MYH9,NOTCH3,PCSK9,RSU1,SELP,SERPING1,ST6GAL1,VCAM1,VCAN | 26 |
| Neurological Disease,Skeletal and Muscular Disorders | Neuromuscular disease | 0.000965 | 3.015472687 |  |  | A2M,APOA4,APOD,APOE,C3,CHI3L1,CST3,LDHA,LDHB,PDLIM1,PON1,ROCK2,SERPING1,SPARCL1,VCAN | 15 |
| Dermatological Diseases and Conditions,Hereditary Disorder,Inflammatory Disease,Inflammatory Response,Organismal Injury and Abnormalities | Netherton syndrome | 0.00105 | 2.978810701 |  |  | DSP,SPINK5 | 2 |
| Inflammatory Response | Acute phase reaction | 0.00105 | 2.978810701 |  |  | APCS,LBP | 2 |
| Cell-To-Cell Signaling and Interaction,Hematological System Development and Function | Interaction of T lymphocytes | 0.00106 | 2.974694135 |  |  | MCAM,SAA1,SELP,VCAM1 | 4 |
| Carbohydrate Metabolism | Uptake of carbohydrate | 0.00106 | 2.974694135 |  | 1.238 | A2M,APOA1,C3,CAMP | 4 |
| Connective Tissue Disorders,Developmental Disorder,Hereditary Disorder,Immunological Disease,Organismal Injury and Abnormalities,Skeletal and Muscular Disorders | Ullrich congenital muscular dystrophy | 0.00106 | 2.974694135 |  |  | ADIPOQ,C3,COL6A2,F10 | 4 |
| Neurological Disease,Organismal Injury and Abnormalities,Psychological Disorders | Disorder of basal ganglia | 0.00109 | 2.962573502 |  |  | A2M,APOA4,APOD,APOE,C3,CHI3L1,LDHA,LDHB,PDLIM1,PON1,ROCK2,SPARCL1,VCAN | 13 |
| Cell Morphology,Tissue Development | Tubulation of cells | 0.0011 | 2.958607315 |  | 1.342 | ADIPOQ,CHI3L1,MMP2,SAA1,ST6GAL1 | 5 |
| Cancer,Organismal Injury and Abnormalities | Invasive tumor | 0.00112 | 2.950781977 |  |  | APOD,APOE,C3,DCD,EFEMP1,EXT1,F10,FBLN5,GSR,MMP2,MYH9,NOTCH3,RNASE1,SERPING1,TFPI,VCAM1 | 16 |
| Cancer,Hematological Disease,Immunological Disease,Organismal Injury and Abnormalities | Peripheral T-cell lymphoma | 0.00112 | 2.950781977 |  |  | C3,COL6A2,FBN1,IGKC,MMP2,SELP,VCAM1 | 7 |
| Cancer,Hematological Disease,Immunological Disease,Organismal Injury and Abnormalities | B-cell neoplasm | 0.00113 | 2.946921557 |  |  | ADIPOQ,ANTXR1,APOE,C3,CHI3L1,CST3,DCD,DSP,EXT2,F10,FBN1,GSR,IGF2R,IGKC,LPA,MCAM,MMP2,PCSK9,PLTP,ROCK2,SELP,SVEP1,TFPI,TIMP2,VCAN,VCL | 26 |
| Lipid Metabolism,Small Molecule Biochemistry,Vitamin and Mineral Metabolism | Metabolism of cholesterol | 0.00115 | 2.93930216 |  |  | APOA1,APOA4,APOB,APOE | 4 |
| Cellular Development,Cellular Growth and Proliferation | Proliferation of blood cells | 0.00115 | 2.93930216 |  | -0.851 | A2M,ADIPOQ,APOE,CAMP,CD84,MYH9,VCAM1,VSIG4 | 8 |
| Cancer,Hematological Disease,Immunological Disease,Organismal Injury and Abnormalities | Mature T-cell neoplasm | 0.00116 | 2.935542011 |  |  | C3,COL6A2,FBN1,GSR,IGFBP4,IGKC,MCAM,MMP2,SELP,TIMP2,VCAM1 | 11 |
| Cancer,Hematological Disease,Immunological Disease,Organismal Injury and Abnormalities | Plasma cell dyscrasia | 0.00117 | 2.931814138 |  |  | ADIPOQ,APOE,CST3,F10,GSR,IGF2R,IGKC,MMP2,PLTP,ROCK2,TFPI,TIMP2 | 12 |
| Cancer,Organismal Injury and Abnormalities | Advanced malignant solid tumor | 0.00117 | 2.931814138 |  |  | C3,EFEMP1,F10,GSR,IGFBP4,MCAM,MMP2,NOTCH3,RNASE1,SERPING1,TFPI,VCAM1,VCAN | 13 |
| Cell Death and Survival | Cell survival | 0.00123 | 2.910094889 | Increased | 2.437 | ADIPOQ,ANTXR1,APOB,APOD,APOE,C3,CAMP,CHI3L1,DCD,IGF2R,LDHA,MCAM,PSMA1,PSMA3,PSMA6,ST6GAL1,TIMP2,VCAM1,VCAN | 19 |
| Cellular Movement | Migration of breast cancer cell lines | 0.00124 | 2.906578315 |  | -0.562 | CCN5,MCAM,MMP2,MYH9,PDLIM1,RSU1,TFPI,TIMP2 | 8 |
| Cellular Movement,Hematological System Development and Function,Immune Cell Trafficking,Inflammatory Response,Lymphoid Tissue Structure and Development | Chemotaxis of lymphocytes | 0.00124 | 2.906578315 |  |  | CAMP,LCP1,PPIB,SAA1 | 4 |
| Developmental Disorder,Hereditary Disorder,Metabolic Disease,Neurological Disease,Organismal Injury and Abnormalities | Familial amyloidotic polyneuropathy | 0.00128 | 2.89279003 |  |  | APOA1,APOE | 2 |
| Cardiovascular Disease,Hematological Disease,Hereditary Disorder,Metabolic Disease,Organismal Injury and Abnormalities | Heterozygous familial hypercholesterolemia | 0.00128 | 2.89279003 |  |  | APOB,PCSK9 | 2 |
| Developmental Disorder,Hereditary Disorder,Metabolic Disease,Neurological Disease,Organismal Injury and Abnormalities,Psychological Disorders | Autosomal dominant Alzheimer disease | 0.00128 | 2.89279003 |  |  | A2M,APOE | 2 |
| Molecular Transport,Small Molecule Biochemistry | Secretion of lactic acid | 0.00128 | 2.89279003 |  |  | A2M,NPC2 | 2 |
| Cellular Movement | Cell rolling of leukemia cell lines | 0.00128 | 2.89279003 |  |  | SELP,VCAM1 | 2 |
| Immunological Disease,Inflammatory Disease,Neurological Disease | Guillain-Barré syndrome | 0.00128 | 2.89279003 |  |  | C3,CST3 | 2 |
| Cancer,Organismal Injury and Abnormalities | Connective tissue tumor | 0.0013 | 2.886056648 |  |  | APOA4,APOB,CAMP,COL6A2,CSPG4,EXT1,EXT2,F10,FBN1,GSR,IGF2R,MCAM,MMP2,MYH9,NOTCH3,PCSK9,ROCK2,RSU1,TKT,VCAN | 20 |
| Cardiovascular Disease,Hereditary Disorder,Organismal Injury and Abnormalities | Familial heart disease | 0.00131 | 2.882728704 |  |  | APOA1,DSC2,DSP,FBN1,PON1,TKT,VCL | 7 |
| Endocrine System Disorders,Gastrointestinal Disease,Metabolic Disease,Organismal Injury and Abnormalities,Renal and Urological Disease | Diabetic nephropathy | 0.00131 | 2.882728704 |  |  | APOD,APOE,CHI3L1,CRHBP,FBLN5,LTBP1,MYH9 | 7 |
| Cellular Development,Cellular Growth and Proliferation,Hematological System Development and Function,Lymphoid Tissue Structure and Development | Cell proliferation of T lymphocytes | 0.00133 | 2.876148359 |  | -0.933 | A2M,APOE,CAMP,CD84,VCAM1,VSIG4 | 6 |
| Organismal Injury and Abnormalities,Respiratory Disease | Severe pulmonary disease | 0.00133 | 2.876148359 |  |  | C3,CAMP,F10,ST6GAL1,TIMP2,TKT | 6 |
| Cancer,Organismal Injury and Abnormalities,Reproductive System Disease | Breast or gynecological cancer | 0.00144 | 2.841637508 |  |  | ADA2,ALDOB,ANTXR1,APOA1,APOA4,APOB,APOD,APOE,C3,CAMP,CCN5,CD5L,CD84,CHI3L1,CILP,COL6A2,CRHBP,CSPG4,DCD,DSC2,DSP,EFEMP1,ERCC6L2,EXT1,EXT2,F10,FBLN5,FBN1,GM2A,GSR,IGDCC4,IGF2R,IGFBP4,IGFBP6,LBP,LMAN1,LPA,LTBP1,MCAM,MMP2,MRC1,MYH9,NOTCH3,NPC2,OAF,PCSK9,PON1,PSMA1,PSMA3,ROCK2,RSU1,SAA1,SELP,SERPING1,SPARCL1,SPINK5,ST6GAL1,SVEP1,TAGLN2,TFPI,TIMP2,TREML1,UGP2,VCAM1,VCAN,VSIG4 | 66 |
| Ophthalmic Disease,Organismal Injury and Abnormalities | Retinal degeneration | 0.00148 | 2.829738285 |  |  | APOE,C3,CST3,EFEMP1,FBLN5,SERPING1,VCAN | 7 |
| Cell Death and Survival | Apoptosis | 0.00151 | 2.821023053 |  | -0.844 | ADIPOQ,APOC1,APOE,C3,CAMP,CCN5,CSPG4,DSP,EFEMP1,IGF2R,IGFBP4,IGFBP6,LDHA,LTBP1,MCAM,MMP2,NOTCH3,PCSK9,RNASE1,ROCK2,SAA1,ST6GAL1,TAGLN2,TIMP2,VCAN | 25 |
| Carbohydrate Metabolism,Nucleic Acid Metabolism,Small Molecule Biochemistry | Pentose shunt of D-glucose | 0.00153 | 2.815308569 |  |  | PGD,TKT | 2 |
| Cardiovascular Disease,Hereditary Disorder,Organismal Injury and Abnormalities,Skeletal and Muscular Disorders | Arrhythmogenic right ventricular dysplasia familial 9 | 0.00153 | 2.815308569 |  |  | DSC2,DSP | 2 |
| Carbohydrate Metabolism,Small Molecule Biochemistry | Synthesis of heparan sulfate proteoglycan | 0.00153 | 2.815308569 |  |  | EXT1,EXT2 | 2 |
| Cardiovascular Disease,Organismal Injury and Abnormalities,Respiratory Disease | Acute pulmonary embolism | 0.00153 | 2.815308569 |  |  | F10,TFPI | 2 |
| Cardiovascular Disease,Cardiovascular System Development and Function,Hereditary Disorder,Organ Morphology,Organismal Development,Organismal Injury and Abnormalities,Skeletal and Muscular Disorders | Dilated cardiomyopathy 1S | 0.00153 | 2.815308569 |  |  | DSP,VCL | 2 |
| Cardiovascular Disease,Organismal Injury and Abnormalities | Calcific aortic valve stenosis | 0.00153 | 2.815308569 |  |  | MMP2,TIMP2 | 2 |
| Cardiovascular Disease,Hematological Disease,Hereditary Disorder,Metabolic Disease,Organismal Injury and Abnormalities | Hyperlipoproteinemia type III | 0.00153 | 2.815308569 |  |  | APOE,PCSK9 | 2 |
| Cardiovascular Disease,Hereditary Disorder,Organismal Injury and Abnormalities,Skeletal and Muscular Disorders | Paroxysmal familial ventricular fibrillation type 1 | 0.00153 | 2.815308569 |  |  | DSP,VCL | 2 |
| Cellular Development,Cellular Growth and Proliferation,Hematological System Development and Function,Hematopoiesis,Lymphoid Tissue Structure and Development,Tissue Development | Monocytopoiesis | 0.00156 | 2.806875402 |  | -1.188 | ADIPOQ,APCS,CAMP,CST3 | 4 |
| Cell-To-Cell Signaling and Interaction,Hematological System Development and Function | Activation of blood cells | 0.00159 | 2.798602876 |  | -1.068 | ADIPOQ,APOE,C3,CAMP,CD84,LBP,SELP,SERPING1 | 8 |
| Dermatological Diseases and Conditions,Organismal Injury and Abnormalities | Keratosis | 0.00162 | 2.790484985 |  |  | DSC2,DSP,PGD,TKT | 4 |
| Hereditary Disorder,Organismal Injury and Abnormalities,Skeletal and Muscular Disorders | Hereditary myopathy | 0.00166 | 2.779891912 |  |  | ADIPOQ,APOA1,C3,COL6A2,DSC2,DSP,F10,FBLN5,FBN1,LBP,RSU1,VCL | 12 |
| Dermatological Diseases and Conditions,Inflammatory Disease,Organismal Injury and Abnormalities | Acne | 0.00167 | 2.777283529 |  |  | APOA1,CRHBP,MMP2,VCAM1 | 4 |
| Cellular Movement,Hematological System Development and Function,Immune Cell Trafficking | Transmigration of mononuclear leukocytes | 0.0017 | 2.769551079 |  |  | MYH9,TIMP2,VCAM1 | 3 |
| Cancer,Endocrine System Disorders,Organismal Injury and Abnormalities,Reproductive System Disease | Ovarian cancer | 0.0017 | 2.769551079 |  |  | ANTXR1,APOA1,APOB,APOE,C3,CCN5,CHI3L1,COL6A2,CRHBP,CSPG4,EXT1,GM2A,IGF2R,IGFBP4,MCAM,MMP2,MYH9,NOTCH3,PCSK9,RSU1,SELP,SERPING1,ST6GAL1,VCAM1,VCAN | 25 |
| Inflammatory Response | Immune response of cells | 0.0017 | 2.769551079 |  | -0.963 | ADIPOQ,APCS,APOA1,APOE,C3,CAMP,CD5L,F10 | 8 |
| Dermatological Diseases and Conditions,Immunological Disease,Inflammatory Disease,Organismal Injury and Abnormalities | Lichen planus | 0.00174 | 2.759450752 |  |  | APOE,DCD,IGFBP6,LCP1 | 4 |
| Cellular Assembly and Organization,Cellular Function and Maintenance | Formation of membrane blebs | 0.0018 | 2.744727495 |  |  | MYH9,ROCK2 | 2 |
| Carbohydrate Metabolism,Connective Tissue Development and Function,Skeletal and Muscular System Development and Function,Small Molecule Biochemistry,Tissue Development | Synthesis of chondroitin sulfate B | 0.0018 | 2.744727495 |  |  | CSPG4,VCAN | 2 |
| Energy Production,Lipid Metabolism,Small Molecule Biochemistry | Oxidation of palmitic acid | 0.0018 | 2.744727495 |  |  | ADIPOQ,APOA1 | 2 |
| Carbohydrate Metabolism,Small Molecule Biochemistry | Catabolism of chondroitin sulfate | 0.0018 | 2.744727495 |  |  | CSPG4,VCAN | 2 |
| Carbohydrate Metabolism | Gluconeogenesis | 0.0018 | 2.744727495 |  |  | ADIPOQ,ALDOB,C1QTNF3 | 3 |
| Inflammatory Disease,Inflammatory Response,Organismal Injury and Abnormalities,Renal and Urological Disease | Glomerulonephritis | 0.00184 | 2.735182177 |  |  | C3,LTBP1,MYH9,PDLIM1,SELP,VCAM1 | 6 |
| Lipid Metabolism,Molecular Transport,Small Molecule Biochemistry | Concentration of cholesterol | 0.00191 | 2.718966633 |  |  | APOA1,APOE,NPC2 | 3 |
| Cell Morphology,Cellular Function and Maintenance | Permeability of cells | 0.00193 | 2.714442691 |  | -0.958 | CAMP,MCAM,RNASE1,TIMP2 | 4 |
| Cardiovascular Disease,Hereditary Disorder,Organismal Injury and Abnormalities | Familial arrhythmia | 0.00199 | 2.701146924 |  |  | DSC2,DSP,FBN1,VCL | 4 |
| Cancer,Hematological Disease,Immunological Disease,Organismal Injury and Abnormalities | B-cell lymphoma | 0.002 | 2.698970004 |  |  | ANTXR1,CHI3L1,CST3,DCD,DSP,EXT2,FBN1,GSR,IGF2R,IGKC,MMP2,PLTP,ROCK2,SVEP1,TIMP2,VCL | 16 |
| Cardiovascular Disease,Organismal Injury and Abnormalities | Long-QT syndrome | 0.00202 | 2.694648631 |  |  | DSC2,DSP,VCL | 3 |
| Organismal Injury and Abnormalities,Reproductive System Disease | Habitual abortion | 0.00202 | 2.694648631 |  |  | C3,F10,TFPI | 3 |
| Developmental Disorder,Hereditary Disorder,Metabolic Disease,Organismal Injury and Abnormalities | Inborn error of lipid metabolism | 0.00202 | 2.694648631 |  |  | APOB,GM2A,LPA | 3 |
| Cancer,Organismal Injury and Abnormalities | Lymphoreticular neoplasm | 0.00205 | 2.688246139 |  |  | ADA2,ANTXR1,APOB,C3,CHI3L1,COL6A2,CSPG4,CST3,DCD,DSP,EXT2,F10,FBN1,GSR,IGF2R,IGFBP4,IGKC,LDHB,LPA,MCAM,MMP2,MRC1,MYH9,PCSK9,PLTP,ROCK2,SELP,SVEP1,TFPI,TIMP2,VCAM1,VCL | 32 |
| Cellular Movement,Hair and Skin Development and Function | Migration of epithelial cell lines | 0.00206 | 2.68613278 |  |  | C3,F10,MCAM,SAA1 | 4 |
| Carbohydrate Metabolism,Molecular Transport | Transport of carbohydrate | 0.00206 | 2.68613278 |  |  | ADIPOQ,APOA1,C3,LBP | 4 |
| Cardiovascular Disease,Organismal Injury and Abnormalities | Critical limb ischemia | 0.00209 | 2.679853714 |  |  | F10,PCSK9 | 2 |
| Cellular Movement,Hematological System Development and Function,Immune Cell Trafficking | Cell rolling of granulocytes | 0.00209 | 2.679853714 |  |  | SELP,VCAM1 | 2 |
| Cardiovascular System Development and Function,Hematological System Development and Function | Systolic pressure | 0.00209 | 2.679853714 |  |  | APOB,PON1 | 2 |
| Inflammatory Disease,Neurological Disease | Chronic inflammatory demyelinating polyradiculoneuropathy | 0.00209 | 2.679853714 |  |  | C3,CST3 | 2 |
| Cell-To-Cell Signaling and Interaction,Hematological System Development and Function,Hypersensitivity Response,Immune Cell Trafficking,Inflammatory Response | Activation of mast cells | 0.00209 | 2.679853714 |  |  | C3,CD84 | 2 |
| Tissue Morphology | Quantity of carcinoma cell lines | 0.00214 | 2.669586227 |  |  | DSP,IGFBP6,ST6GAL1 | 3 |
| Tissue Development | Growth of epithelial tissue | 0.00217 | 2.663540266 |  | 0.178 | ADIPOQ,APOE,C3,CAMP,IGFBP4,MCAM,SAA1,TIMP2 | 8 |
| Cancer,Hematological Disease,Immunological Disease,Organismal Injury and Abnormalities | Mature B-cell neoplasm | 0.00218 | 2.661543506 |  |  | ADIPOQ,ANTXR1,APOE,CST3,DCD,DSP,EXT2,F10,FBN1,GSR,IGF2R,IGKC,MMP2,PCSK9,PLTP,ROCK2,SVEP1,TFPI,TIMP2,VCAN,VCL | 21 |
| Cell Death and Survival | Cell viability | 0.0022 | 2.657577319 | Increased | 2.569 | ADIPOQ,ANTXR1,APOB,APOD,APOE,C3,CHI3L1,DCD,IGF2R,LDHA,MCAM,PSMA1,PSMA3,PSMA6,ST6GAL1,TIMP2,VCAM1,VCAN | 18 |
| Cardiovascular Disease,Organismal Injury and Abnormalities,Skeletal and Muscular Disorders | Cardiomyopathy of heart ventricle | 0.00226 | 2.645891561 |  |  | DSC2,DSP,VCL | 3 |
| Cell-mediated Immune Response,Cellular Movement,Hematological System Development and Function,Immune Cell Trafficking | Cell movement of T lymphocytes | 0.00227 | 2.643974143 |  |  | CAMP,LCP1,MYH9,SELP | 4 |
| Organismal Injury and Abnormalities,Renal and Urological Disease | End stage renal disease | 0.00227 | 2.643974143 |  |  | C3,F10,MYH9,PCSK9 | 4 |
| Inflammatory Response,Respiratory Disease | Inflammation of respiratory system component | 0.00232 | 2.634512015 |  |  | ANTXR1,APOA1,C3,CAMP,CHI3L1,F10,SERPING1,VCAM1 | 8 |
| Hereditary Disorder,Immunological Disease,Organismal Injury and Abnormalities | Autosomal recessive immunological disorder | 0.00234 | 2.630784143 |  |  | ADA2,APOE,C3,COL6A2,IGKC,SERPING1 | 6 |
| Cancer,Hematological Disease,Immunological Disease,Organismal Injury and Abnormalities | Angioimmunoblastic T-cell lymphoma | 0.00237 | 2.625251654 |  |  | C3,COL6A2,IGKC,SELP,VCAM1 | 5 |
| Cancer,Organismal Injury and Abnormalities | Breast or colorectal cancer | 0.00237 | 2.625251654 |  |  | A2M,ADA2,ANTXR1,APOA1,APOA4,APOB,APOC1,APOD,APOE,C1QTNF3,C3,CAMP,CCN5,CD5L,CHI3L1,COL6A2,CSPG4,CST3,DCD,DSC2,DSP,EFEMP1,EXT2,F10,FBLN5,FBN1,GSR,IGDCC4,IGF2R,IGFBP4,IGFBP6,LBP,LCP1,LDHA,LMAN1,LPA,LTBP1,MCAM,MMP2,MRC1,MYH9,NOTCH3,NPC2,OAF,PCSK9,PDLIM1,PON1,PROZ,PVR,ROCK2,SAA4,SELP,SERPING1,SPARCL1,SPINK5,ST6GAL1,SVEP1,TAGLN2,TFPI,TIMP2,TREML1,VCAM1,VCAN,VCL,VSIG4 | 65 |
| Lipid Metabolism,Small Molecule Biochemistry | Metabolism of triacylglycerol | 0.00238 | 2.623423043 |  |  | APOC3,APOE,C3 | 3 |
| Dermatological Diseases and Conditions,Endocrine System Disorders,Gastrointestinal Disease,Metabolic Disease,Organismal Injury and Abnormalities,Skeletal and Muscular Disorders | Diabetic foot ulcer disorder | 0.00238 | 2.623423043 |  |  | COL6A2,F10,TFPI | 3 |
| Protein Degradation,Protein Synthesis | Degradation of Gelatin | 0.00241 | 2.617982957 |  |  | CSPG4,MMP2 | 2 |
| Cellular Movement | Migration of rhabdomyosarcoma cell lines | 0.00241 | 2.617982957 |  |  | IGFBP6,MCAM | 2 |
| Cardiovascular Disease,Organismal Injury and Abnormalities,Skeletal and Muscular Disorders | Cardiac fibrillation | 0.00242 | 2.616184634 |  |  | DSP,F10,TFPI,VCAM1,VCL | 5 |
| Carbohydrate Metabolism,Small Molecule Biochemistry | Synthesis of glycosaminoglycan | 0.00243 | 2.614393726 |  |  | CSPG4,EXT1,EXT2,VCAN | 4 |
| Cell Death and Survival | Cell viability of myeloma cell lines | 0.00243 | 2.614393726 |  | 1.982 | LDHA,PSMA1,PSMA3,PSMA6 | 4 |
| Gene Expression,RNA Damage and Repair,RNA Post-Transcriptional Modification | Stabilization of mRNA | 0.0025 | 2.602059991 |  |  | PSMA1,PSMA3,PSMA6,PSMB7 | 4 |
| Cardiovascular Disease,Organismal Injury and Abnormalities | Angina pectoris | 0.0025 | 2.602059991 |  |  | ADIPOQ,F10,PCSK9,TFPI | 4 |
| Cell-To-Cell Signaling and Interaction | Binding of cell surface | 0.00251 | 2.600326279 |  |  | APOE,C3,EXT1 | 3 |
| Cardiovascular Disease,Organismal Injury and Abnormalities | Acute heart failure | 0.00251 | 2.600326279 |  |  | DSC2,F10,VCL | 3 |
| Cancer,Organismal Injury and Abnormalities,Reproductive System Disease | Malignant neoplasm of male genital organ | 0.00253 | 2.596879479 |  |  | A2M,ALDOB,ANTXR1,APMAP,APOA1,APOB,APOD,C3,CHAD,CHI3L1,CILP,COL6A2,CRHBP,CSPG4,DSC2,DSP,EFEMP1,F10,FBLN5,FBN1,GSR,IGDCC4,IGF2R,IGFBP4,IGKC,LCP1,LMAN1,MMP2,MYH9,NOTCH3,PSMA1,ROCK2,SERPING1,SPINK5,ST6GAL1,SVEP1,TFPI,TIMP2,TKT,VCAM1,VCAN,VCL | 42 |
| Cardiovascular System Development and Function,Hematological System Development and Function | Blood pressure | 0.00264 | 2.578396073 |  |  | ADIPOQ,APOB,PON1 | 3 |
| Cellular Development,Tissue Development | Differentiation of epithelial tissue | 0.00271 | 2.567030709 |  |  | ADIPOQ,DSP,ROCK2,SPINK5,TAGLN2 | 5 |
| Cancer,Hematological Disease,Immunological Disease,Organismal Injury and Abnormalities | Low-grade lymphoma | 0.00272 | 2.565431096 |  |  | CST3,DCD,DSP,EXT2,FBN1,IGF2R,MMP2,PLTP,ROCK2,SVEP1,TIMP2,VCL | 12 |
| Cancer,Hematological Disease,Organismal Injury and Abnormalities | Hematologic cancer | 0.00273 | 2.563837353 |  |  | ADA2,ADIPOQ,ANTXR1,APOB,APOE,C3,CHI3L1,COL6A2,CSPG4,CST3,DCD,DSP,EXT2,F10,FBN1,GSR,IGF2R,IGFBP4,IGKC,LDHA,LDHB,LPA,LTBP1,MCAM,MMP2,MRC1,MYH9,NOTCH3,PCSK9,PLTP,ROCK2,SELP,SVEP1,TFPI,TIMP2,VCAM1,VCAN,VCL | 38 |
| Cardiovascular System Development and Function,Cell Morphology,Cellular Development,Cellular Function and Maintenance,Cellular Growth and Proliferation,Organismal Development,Tissue Development | Morphogenesis of vascular endothelial cells | 0.00274 | 2.562249437 |  |  | MMP2,TIMP2 | 2 |
| Cardiovascular Disease,Connective Tissue Disorders,Developmental Disorder,Hereditary Disorder,Organismal Injury and Abnormalities,Skeletal and Muscular Disorders | Loeys-Dietz syndrome | 0.00274 | 2.562249437 |  |  | FBN1,VCAN | 2 |
| Endocrine System Disorders,Organismal Injury and Abnormalities | Benign cold thyroid nodule | 0.00274 | 2.562249437 |  |  | APCS,APOA1 | 2 |
| Skeletal and Muscular Disorders | Muscle laminopathy | 0.00275 | 2.560667306 |  |  | COL6A2,DSC2,DSP,RSU1 | 4 |
| Hereditary Disorder,Organismal Injury and Abnormalities,Skeletal and Muscular Disorders | Autosomal dominant myopathy | 0.00275 | 2.560667306 |  |  | COL6A2,DSC2,DSP,FBN1,RSU1,VCL | 6 |
| Carbohydrate Metabolism,Lipid Metabolism,Small Molecule Biochemistry | Metabolism of phosphatidylcholine | 0.00278 | 2.555955204 |  |  | APOA1,APOA4,PON1 | 3 |
| Cell Signaling,Cellular Function and Maintenance,Molecular Transport,Vitamin and Mineral Metabolism | Flux of Ca2+ | 0.00284 | 2.54668166 |  |  | APOA1,C3,CAMP,CD84,PPIB | 5 |
| Cancer,Organismal Injury and Abnormalities,Reproductive System Disease | Breast or ovarian carcinoma | 0.00284 | 2.54668166 |  |  | ADA2,ANTXR1,APOB,APOD,APOE,C3,CHI3L1,COL6A2,CRHBP,CSPG4,DCD,DSP,EFEMP1,EXT1,EXT2,F10,FBLN5,FBN1,GM2A,IGDCC4,IGF2R,IGFBP4,LMAN1,LTBP1,MCAM,MMP2,MYH9,NOTCH3,OAF,PCSK9,ROCK2,RSU1,SELP,SERPING1,ST6GAL1,SVEP1,TREML1,VCAN | 38 |
| Cardiovascular System Development and Function,Cell Morphology,Cellular Development,Organismal Development,Tissue Development | Tubulation of endothelial cells | 0.00292 | 2.534617149 |  | 1 | ADIPOQ,MMP2,SAA1,ST6GAL1 | 4 |
| Cardiovascular Disease,Neurological Disease,Organismal Injury and Abnormalities,Psychological Disorders | Vascular dementia | 0.00292 | 2.534617149 |  |  | APOA1,APOB,NOTCH3 | 3 |
| Cell-To-Cell Signaling and Interaction,Cellular Function and Maintenance,Inflammatory Response | Phagocytosis of phagocytes | 0.00292 | 2.534617149 |  |  | APCS,C3,CAMP | 3 |
| Carbohydrate Metabolism | Synthesis of carbohydrate | 0.00295 | 2.530177984 |  |  | APOA1,APOE,CSPG4,EXT1,EXT2,TKT,UGP2,VCAN | 8 |
| Cancer,Organismal Injury and Abnormalities,Skeletal and Muscular Disorders | Leiomyoma | 0.00297 | 2.527243551 |  |  | CCN5,FBN1,LTBP1,MMP2,PSMA6,SPARCL1,VCAN | 7 |
| Cell-To-Cell Signaling and Interaction | Adhesion of colorectal cancer cell lines | 0.00307 | 2.512861625 |  |  | FBN1,MMP2,ST6GAL1 | 3 |
| Cell Death and Survival | Cell death of tumor cell lines | 0.00308 | 2.511449283 |  | 0.517 | ADIPOQ,APMAP,APOB,APOE,CAMP,CCN5,CSPG4,DSP,EFEMP1,IGF2R,IGFBP4,IGFBP6,LDHA,LTBP1,NOTCH3,NPC2,RNASE1,SAA1,ST6GAL1,TAGLN2,VCAM1,VCAN | 22 |
| Cancer,Organismal Injury and Abnormalities,Reproductive System Disease | Invasive breast carcinoma | 0.0031 | 2.508638306 |  |  | APOD,DCD,EFEMP1,FBLN5,MMP2,MYH9,SERPING1 | 7 |
| Cell-To-Cell Signaling and Interaction,Hematological System Development and Function,Immune Cell Trafficking,Inflammatory Response | Binding of dendritic cells | 0.0031 | 2.508638306 |  |  | A2M,MRC1 | 2 |
| Cardiovascular Disease,Hematological Disease,Hereditary Disorder,Metabolic Disease,Organismal Injury and Abnormalities | Hyperlipoproteinemia type I | 0.0031 | 2.508638306 |  |  | APOC2,APOC3 | 2 |
| Cellular Function and Maintenance | Internalization of E. coli | 0.0031 | 2.508638306 |  |  | CAMP,MRC1 | 2 |
| Neurological Disease,Organismal Injury and Abnormalities | Recurrent encephalopathy | 0.0031 | 2.508638306 |  |  | F10,GSR | 2 |
| Cell-To-Cell Signaling and Interaction,Inflammatory Response | Immune response of leukocytes | 0.00319 | 2.496209317 |  | -0.957 | APCS,APOE,C3,CAMP | 4 |
| Endocrine System Disorders,Organismal Injury and Abnormalities | Benign thyroid disease | 0.00319 | 2.496209317 |  |  | APCS,APOA1,APOB,PSMA3 | 4 |
| Cancer,Hematological Disease,Organismal Injury and Abnormalities | Myeloid or lymphoid neoplasm | 0.00322 | 2.492144128 |  |  | ADA2,ADIPOQ,ANTXR1,APOB,APOE,C3,CHI3L1,COL6A2,CSPG4,CST3,DCD,DSP,EXT2,F10,FBN1,GSR,IGF2R,IGFBP4,IGKC,LDHA,LDHB,LPA,LTBP1,MCAM,MMP2,MRC1,MYH9,NOTCH3,PCSK9,PLTP,ROCK2,SELP,SVEP1,TFPI,TIMP2,VCAM1,VCAN,VCL | 38 |
| Cancer,Organismal Injury and Abnormalities,Skeletal and Muscular Disorders | Muscle tumor | 0.0033 | 2.48148606 |  |  | CCN5,CSPG4,FBN1,LTBP1,MMP2,MYH9,PCSK9,PSMA6,ROCK2,RSU1,SPARCL1,VCAN | 12 |
| Cancer,Organismal Injury and Abnormalities,Reproductive System Disease | Prostate cancer | 0.00333 | 2.477555766 |  |  | A2M,ALDOB,ANTXR1,APMAP,APOA1,APOB,APOD,C3,CHAD,CHI3L1,CILP,COL6A2,CRHBP,CSPG4,DSC2,DSP,EFEMP1,F10,FBLN5,FBN1,GSR,IGF2R,IGFBP4,IGKC,LCP1,LMAN1,MMP2,MYH9,NOTCH3,PSMA1,ROCK2,SERPING1,SPINK5,ST6GAL1,SVEP1,TFPI,TIMP2,TKT,VCAM1,VCAN,VCL | 41 |
| Gastrointestinal Disease,Hepatic System Disease,Hereditary Disorder,Organismal Injury and Abnormalities | Progressive familial intrahepatic cholestasis type 1 | 0.00337 | 2.472370099 |  |  | LBP,LPA,UGP2 | 3 |
| Lipid Metabolism,Molecular Transport,Small Molecule Biochemistry | Uptake of lipid | 0.00337 | 2.472370099 |  |  | APOD,APOE,CAMP | 3 |
| Cellular Movement | Migration of melanoma cell lines | 0.00338 | 2.4710833 |  |  | CST3,MCAM,TIMP2,VCAN | 4 |
| Hematological Disease | Factor VIII deficiency | 0.00348 | 2.458420756 |  |  | F10,LMAN1 | 2 |
| Cell-To-Cell Signaling and Interaction,Cellular Compromise,Tumor Morphology | Adhesion of cancer cells | 0.00348 | 2.458420756 |  |  | MCAM,VCAM1 | 2 |
| Cardiovascular Disease,Hematological Disease,Organismal Injury and Abnormalities | Thrombosis of vein | 0.00353 | 2.452225295 |  |  | F10,MMP2,TFPI | 3 |
| Cancer,Organismal Injury and Abnormalities | Anogenital cancer | 0.00355 | 2.449771647 |  |  | A2M,ADA2,ALDOB,ANTXR1,APMAP,APOA1,APOA4,APOB,APOD,APOE,C3,CAMP,CCN5,CD5L,CD84,CHAD,CHI3L1,CILP,COL6A2,CRHBP,CSPG4,DCD,DSC2,DSP,EFEMP1,ERCC6L2,EXT1,EXT2,F10,FBLN5,FBN1,GM2A,GSR,IGDCC4,IGF2R,IGFBP4,IGFBP6,IGKC,LBP,LCP1,LMAN1,LPA,LTBP1,MCAM,MMP2,MRC1,MYH9,NOTCH3,NPC2,OAF,PCSK9,PON1,PSMA1,PSMA3,ROCK2,RSU1,SAA1,SELP,SERPING1,SPARCL1,SPINK5,ST6GAL1,SVEP1,TAGLN2,TFPI,TIMP2,TKT,TREML1,UGP2,VCAM1,VCAN,VCL,VSIG4 | 73 |
| Connective Tissue Disorders,Immunological Disease,Inflammatory Disease,Organismal Injury and Abnormalities,Skeletal and Muscular Disorders | Lupus erythematosus | 0.00363 | 2.440093375 |  |  | C3,CAMP,F10,LTBP1,MYH9,SELP,SERPING1,VCAM1 | 8 |
| Lipid Metabolism,Small Molecule Biochemistry,Vitamin and Mineral Metabolism | Steroid metabolism | 0.00366 | 2.436518915 |  |  | APOA1,APOA4,APOB,APOE,IGFBP4 | 5 |
| Cardiovascular Disease,Organismal Injury and Abnormalities | Unstable angina | 0.0037 | 2.431798276 |  |  | F10,PCSK9,TFPI | 3 |
| Cell-To-Cell Signaling and Interaction,Cellular Assembly and Organization | Cell-cell adhesion | 0.00373 | 2.428291168 |  |  | APOA4,CST3,DSP,SELP,VCAM1 | 5 |
| Cancer,Organismal Injury and Abnormalities,Renal and Urological Disease | Urothelial bladder carcinoma | 0.00376 | 2.424812155 |  |  | APOD,CSPG4,FBLN5,MMP2,NOTCH3,SERPING1,TIMP2 | 7 |
| Cellular Development,Cellular Growth and Proliferation | Cell proliferation of tumor cell lines | 0.00384 | 2.415668776 |  | -0.139 | A2M,ADIPOQ,APOB,CAMP,DCD,DSC2,EFEMP1,EXT1,IGF2R,IGFBP4,LCP1,LDHA,LTBP1,MMP2,NOTCH3,PGD,PSMB7,RSU1,SPARCL1,TAGLN2,TFPI,TIMP2,VCAM1,VCAN | 24 |
| Cancer,Hematological Disease,Immunological Disease,Organismal Injury and Abnormalities | B-cell non-Hodgkin lymphoma | 0.00387 | 2.412289035 |  |  | ANTXR1,CST3,DCD,DSP,EXT2,FBN1,GSR,IGF2R,IGKC,MMP2,PLTP,ROCK2,SVEP1,TIMP2,VCL | 15 |
| Metabolic Disease,Neurological Disease,Organismal Injury and Abnormalities,Psychological Disorders | Early-onset Alzheimer disease | 0.00387 | 2.412289035 |  |  | APOE,CST3 | 2 |
| Organismal Injury and Abnormalities,Renal and Urological Disease | Minimal change nephrotic syndrome | 0.00387 | 2.412289035 |  |  | CST3,PDLIM1 | 2 |
| Cardiovascular Disease,Hematological Disease,Hereditary Disorder,Metabolic Disease,Organismal Injury and Abnormalities | Homozygous familial hypercholesterolemia | 0.00387 | 2.412289035 |  |  | APOB,PCSK9 | 2 |
| Inflammatory Disease,Inflammatory Response,Organismal Injury and Abnormalities,Renal and Urological Disease | Lupus nephritis | 0.00389 | 2.410050399 |  |  | C3,LTBP1,PDLIM1,SELP,VCAM1 | 5 |
| Cancer,Organismal Injury and Abnormalities | Sarcoma | 0.00392 | 2.406713933 |  |  | APOA4,APOB,CAMP,CSPG4,EXT1,EXT2,F10,FBN1,GSR,IGF2R,MCAM,MMP2,MYH9,PCSK9,ROCK2,RSU1,TKT,VCAN | 18 |
| Connective Tissue Disorders,Inflammatory Disease,Inflammatory Response,Organismal Injury and Abnormalities,Skeletal and Muscular Disorders | Osteoarthritis | 0.00404 | 2.393618635 |  |  | C3,F10,MMP2,TIMP2,VCAM1,VSIG4 | 6 |
| Cardiovascular System Development and Function,Tissue Morphology | Permeability of vascular system | 0.00404 | 2.393618635 |  |  | MCAM,MMP2,RNASE1 | 3 |
| Infectious Diseases,Inflammatory Disease,Organismal Injury and Abnormalities,Respiratory Disease | Severe acute respiratory syndrome | 0.00421 | 2.375717904 |  |  | CAMP,F10,ST6GAL1,TIMP2,TKT | 5 |
| Cell-To-Cell Signaling and Interaction,Renal and Urological System Development and Function | Adhesion of kidney cells | 0.00421 | 2.375717904 |  |  | ANTXR1,C3,F10 | 3 |
| Cellular Movement,Skeletal and Muscular System Development and Function | Migration of smooth muscle cells | 0.00421 | 2.375717904 |  |  | ADIPOQ,APOE,IGFBP4 | 3 |
| Cellular Development,Cellular Growth and Proliferation | Cell proliferation of hepatoma cell lines | 0.00423 | 2.373659633 |  | 0.647 | ADIPOQ,LDHA,LTBP1,RSU1,VCAM1,VCAN | 6 |
| Inflammatory Disease,Inflammatory Response,Organismal Injury and Abnormalities,Respiratory Disease | Asthma | 0.00423 | 2.373659633 |  |  | ANTXR1,APOA1,C3,CHI3L1,SERPING1,VCAM1 | 6 |
| Cancer,Hematological Disease,Organismal Injury and Abnormalities | Neoplasia of blood cells | 0.00424 | 2.372634143 |  |  | ADA2,ADIPOQ,ANTXR1,APOB,APOE,C3,CHI3L1,COL6A2,CSPG4,CST3,DCD,DSP,EXT2,F10,FBN1,GSR,IGF2R,IGFBP4,IGKC,LDHA,LDHB,LPA,LTBP1,MCAM,MMP2,MRC1,MYH9,PCSK9,PLTP,ROCK2,SELP,SVEP1,TFPI,TIMP2,VCAM1,VCAN,VCL | 37 |
| Cell-To-Cell Signaling and Interaction,Hematological System Development and Function,Hypersensitivity Response,Immune Cell Trafficking | Adhesion of eosinophils | 0.00429 | 2.367542708 |  |  | SELP,VCAM1 | 2 |
| Inflammatory Response,Organismal Injury and Abnormalities | Antibody mediated rejection | 0.00429 | 2.367542708 |  |  | C3,SERPING1 | 2 |
| Cellular Movement,Hematological System Development and Function,Hypersensitivity Response,Immune Cell Trafficking,Inflammatory Response | Chemotaxis of mast cells | 0.00429 | 2.367542708 |  |  | C3,CAMP | 2 |
| Cancer,Endocrine System Disorders,Organismal Injury and Abnormalities | Thyroid follicular adenoma | 0.00429 | 2.367542708 |  |  | APCS,APOA1 | 2 |
| Cancer,Organismal Injury and Abnormalities | Serous adenocarcinoma | 0.00435 | 2.361510743 |  |  | ANTXR1,APOD,APOE,C3,COL6A2,CSPG4,FBLN5,IGFBP4,MCAM,NOTCH3,RSU1,SAA1,SERPING1,SPARCL1,VCAN | 15 |
| Small Molecule Biochemistry | Catabolism of carboxylic acid | 0.0044 | 2.356547324 |  |  | CSPG4,PON1,VCAN | 3 |
| Cell-To-Cell Signaling and Interaction,Cellular Assembly and Organization,Tissue Development | Adhesion of cell-associated matrix | 0.0044 | 2.356547324 |  |  | FBLN5,VCAM1,VCL | 3 |
| Cancer,Organismal Injury and Abnormalities | Metastasis | 0.00453 | 2.343901798 |  |  | APOE,C3,EFEMP1,EXT1,F10,GSR,MMP2,MYH9,NOTCH3,RNASE1,TFPI,VCAM1 | 12 |
| Cell-To-Cell Signaling and Interaction | Communication of cells | 0.00457 | 2.3400838 |  |  | ADIPOQ,APOA1,APOC3,APOE,C3,CAMP,CRHBP,CSPG4,EXT1,EXT2,IGF2R,IGFBP4,ROCK2,SAA1 | 14 |
| Cell-To-Cell Signaling and Interaction,Hematological System Development and Function,Immune Cell Trafficking | Adhesion of granulocytes | 0.00458 | 2.339134522 |  |  | PLTP,SELP,VCAM1 | 3 |
| Developmental Disorder,Hereditary Disorder,Immunological Disease,Organismal Injury and Abnormalities | Complement component deficiency | 0.00472 | 2.326058001 |  |  | C3,SERPING1 | 2 |
| Cardiovascular System Development and Function,Cell-To-Cell Signaling and Interaction | Binding of microvascular endothelial cells | 0.00472 | 2.326058001 |  |  | MCAM,VCAM1 | 2 |
| Cell-To-Cell Signaling and Interaction,Cellular Function and Maintenance,Inflammatory Response | Phagocytosis of bacteria | 0.00472 | 2.326058001 |  |  | C3,CAMP | 2 |
| Hereditary Disorder,Ophthalmic Disease,Organismal Injury and Abnormalities | Autosomal dominant maculopathy | 0.00472 | 2.326058001 |  |  | APOE,FBLN5 | 2 |
| Cell Death and Survival,Organismal Survival | Survival of bacteria | 0.00472 | 2.326058001 |  |  | C3,CAMP | 2 |
| Cellular Movement,Hematological System Development and Function,Immune Cell Trafficking,Inflammatory Response | Transendothelial migration of monocytes | 0.00472 | 2.326058001 |  |  | TIMP2,VCAM1 | 2 |
| Cellular Function and Maintenance,Hematological System Development and Function | Engulfment of myeloid cells | 0.00477 | 2.321481621 |  |  | APCS,CAMP,MRC1 | 3 |
| Cell Morphology,Cellular Assembly and Organization,Cellular Function and Maintenance | Reorganization of actin cytoskeleton | 0.00477 | 2.321481621 |  |  | ANTXR1,LCP1,MYH9 | 3 |
| Hereditary Disorder,Organismal Injury and Abnormalities,Renal and Urological Disease | Autosomal dominant kidney disease | 0.00481 | 2.317854924 |  |  | ANTXR1,C3,SAA4,TIMP2 | 4 |
| Cancer,Organismal Injury and Abnormalities | Development of benign tumor | 0.00488 | 2.311580178 |  |  | ANTXR1,APCS,APOA1,APOE,COL6A2,MYH9,SPARCL1,UGP2,VSIG4 | 9 |
| Infectious Diseases | Activation of Influenza B virus | 0.00491 | 2.308918508 |  |  | APCS | 1 |
| Carbohydrate Metabolism,Lipid Metabolism,Small Molecule Biochemistry | Activation of 1-palmitoyl-2-glutaroyl-sn-glycero-3-phosphorylcholine | 0.00491 | 2.308918508 |  |  | PON1 | 1 |
| Cardiovascular System Development and Function,Embryonic Development,Organismal Development,Tissue Development | Angiogenesis of embryonic tissue | 0.00491 | 2.308918508 |  |  | TIMP2 | 1 |
| Connective Tissue Disorders,Developmental Disorder,Hereditary Disorder,Organismal Injury and Abnormalities,Skeletal and Muscular Disorders | 3MC syndrome type 3 | 0.00491 | 2.308918508 |  |  | COLEC10 | 1 |
| Tissue Development | Adhesion of articular cartilage | 0.00491 | 2.308918508 |  |  | RNASE1 | 1 |
| Ophthalmic Disease,Organismal Injury and Abnormalities | Age-related macular degeneration type 3 | 0.00491 | 2.308918508 |  |  | FBLN5 | 1 |
| Cardiovascular System Development and Function,Cell-To-Cell Signaling and Interaction | Activation of HCAEC cells | 0.00491 | 2.308918508 |  |  | LBP | 1 |
| Carbohydrate Metabolism,Lipid Metabolism,Small Molecule Biochemistry | Activation of 1-palmitoyl-2-arachidonyl-sn-glycero-3-phosphorylcholine | 0.00491 | 2.308918508 |  |  | PON1 | 1 |
| Ophthalmic Disease,Organismal Injury and Abnormalities | Age-related macular degeneration type 11 | 0.00491 | 2.308918508 |  |  | CST3 | 1 |
| Connective Tissue Disorders,Developmental Disorder,Hereditary Disorder,Organismal Injury and Abnormalities,Skeletal and Muscular Disorders | Acromicric dysplasia | 0.00491 | 2.308918508 |  |  | FBN1 | 1 |
| Nervous System Development and Function | Activation of superior temporal gyrus | 0.00491 | 2.308918508 |  |  | APOE | 1 |

**Figure S1. OPLS-DA model**


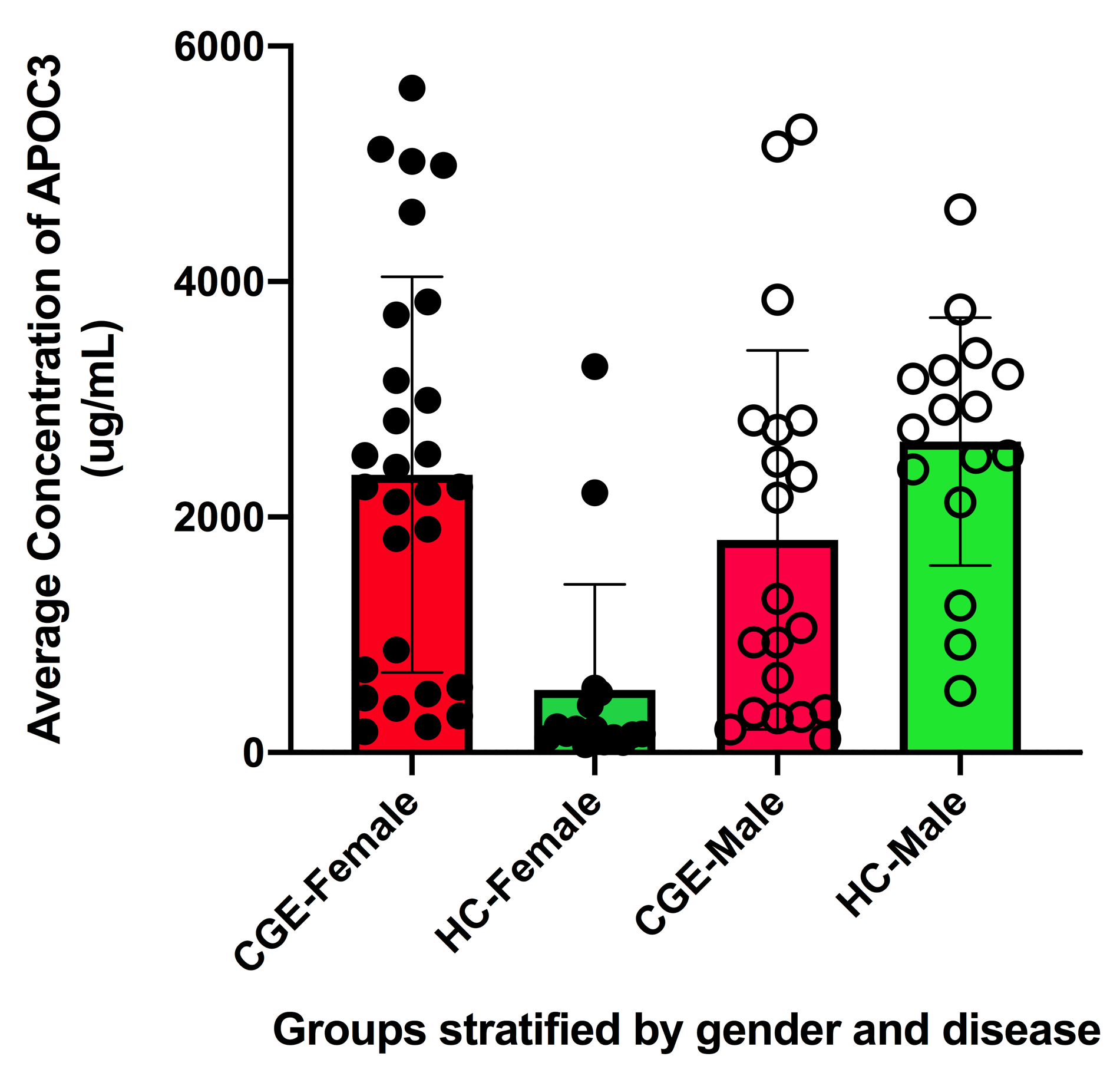

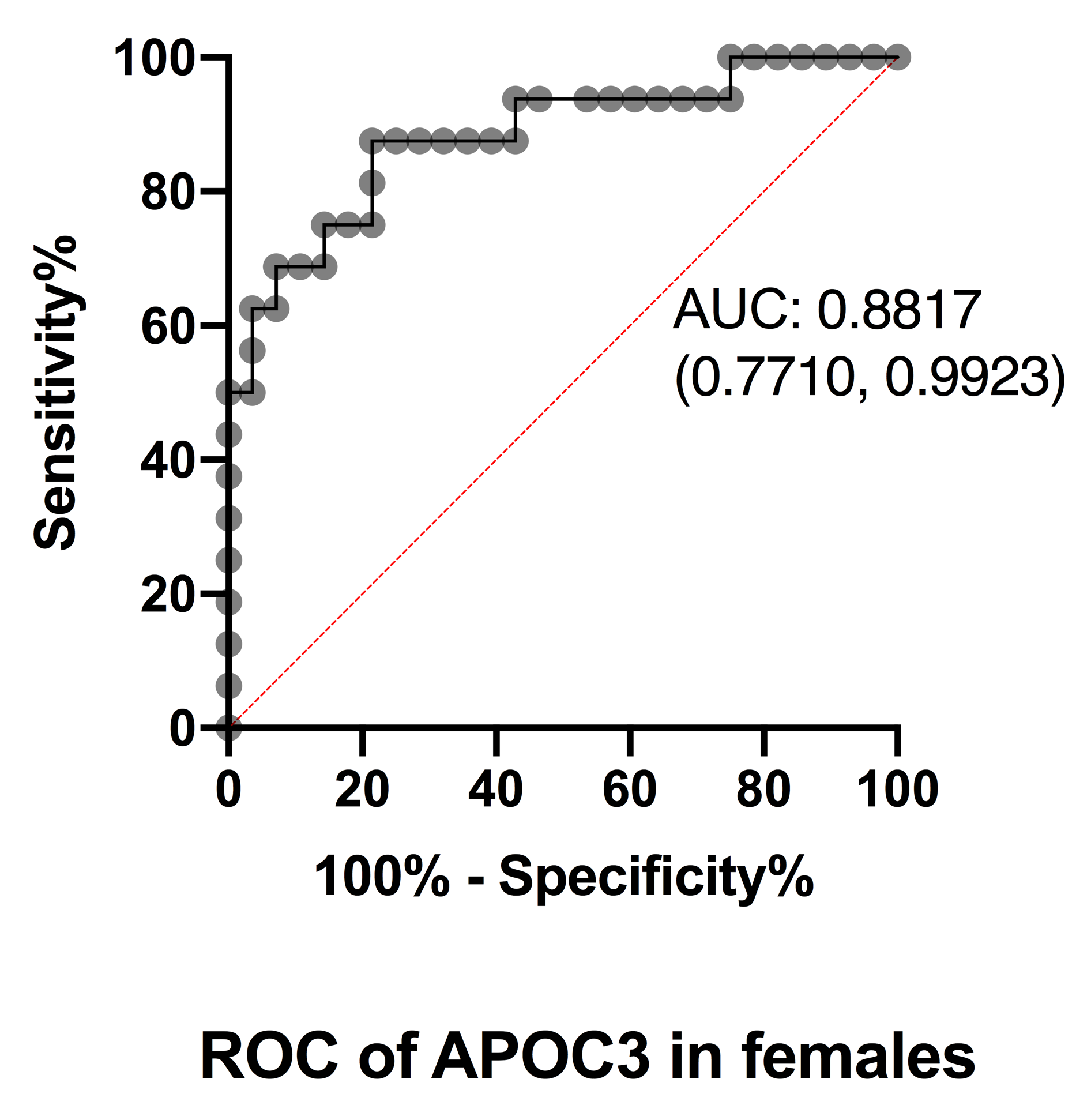

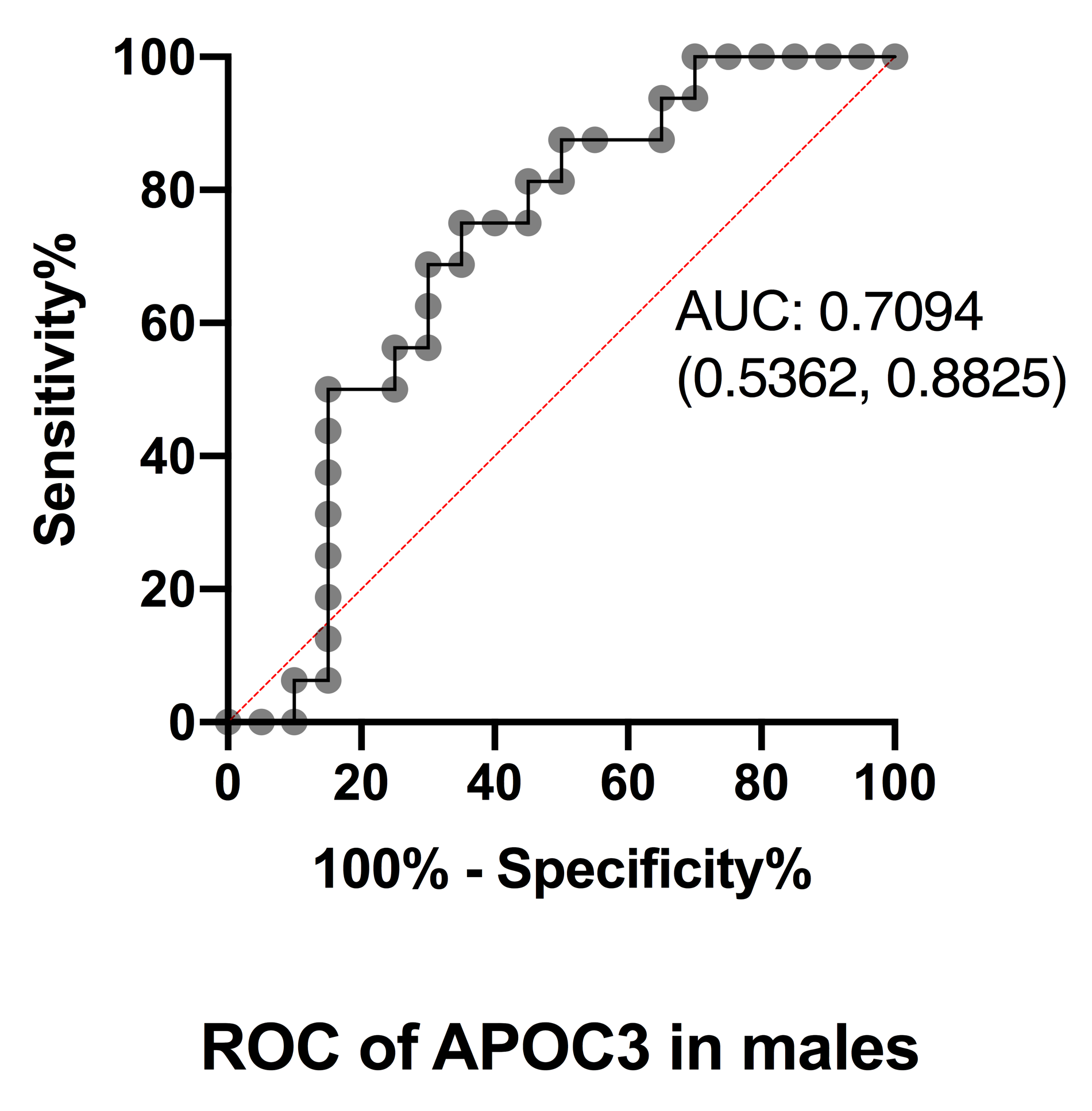

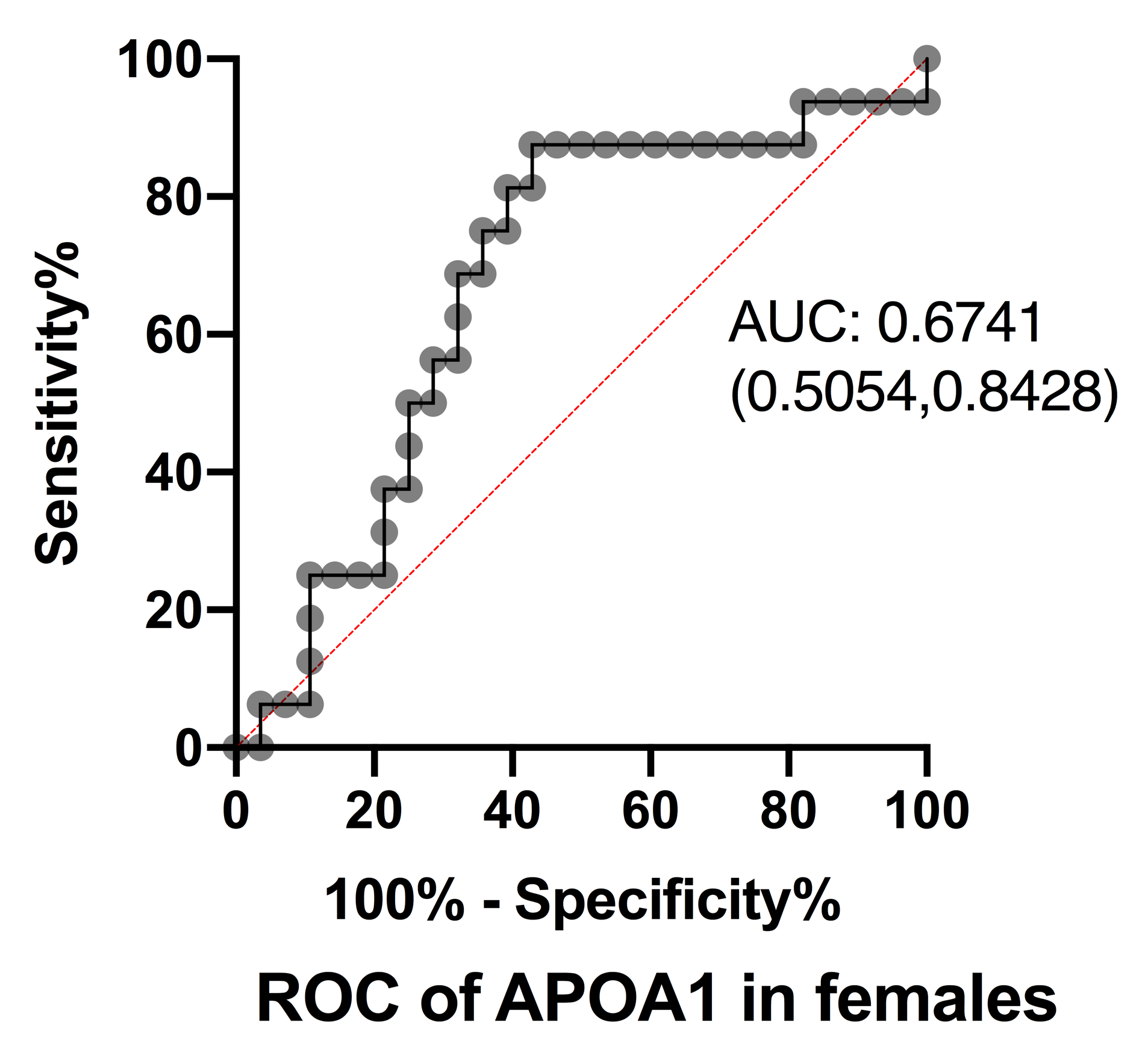

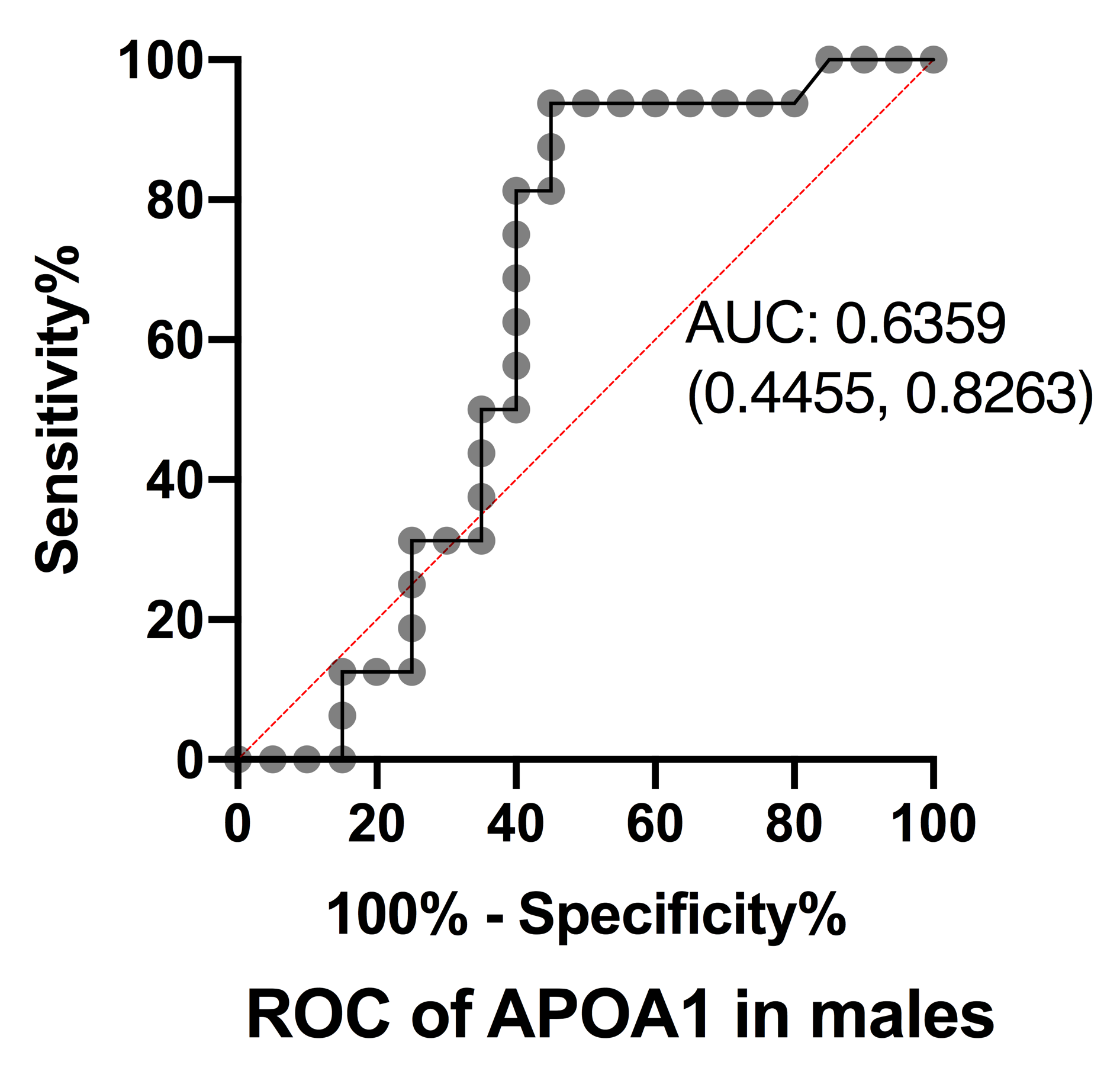

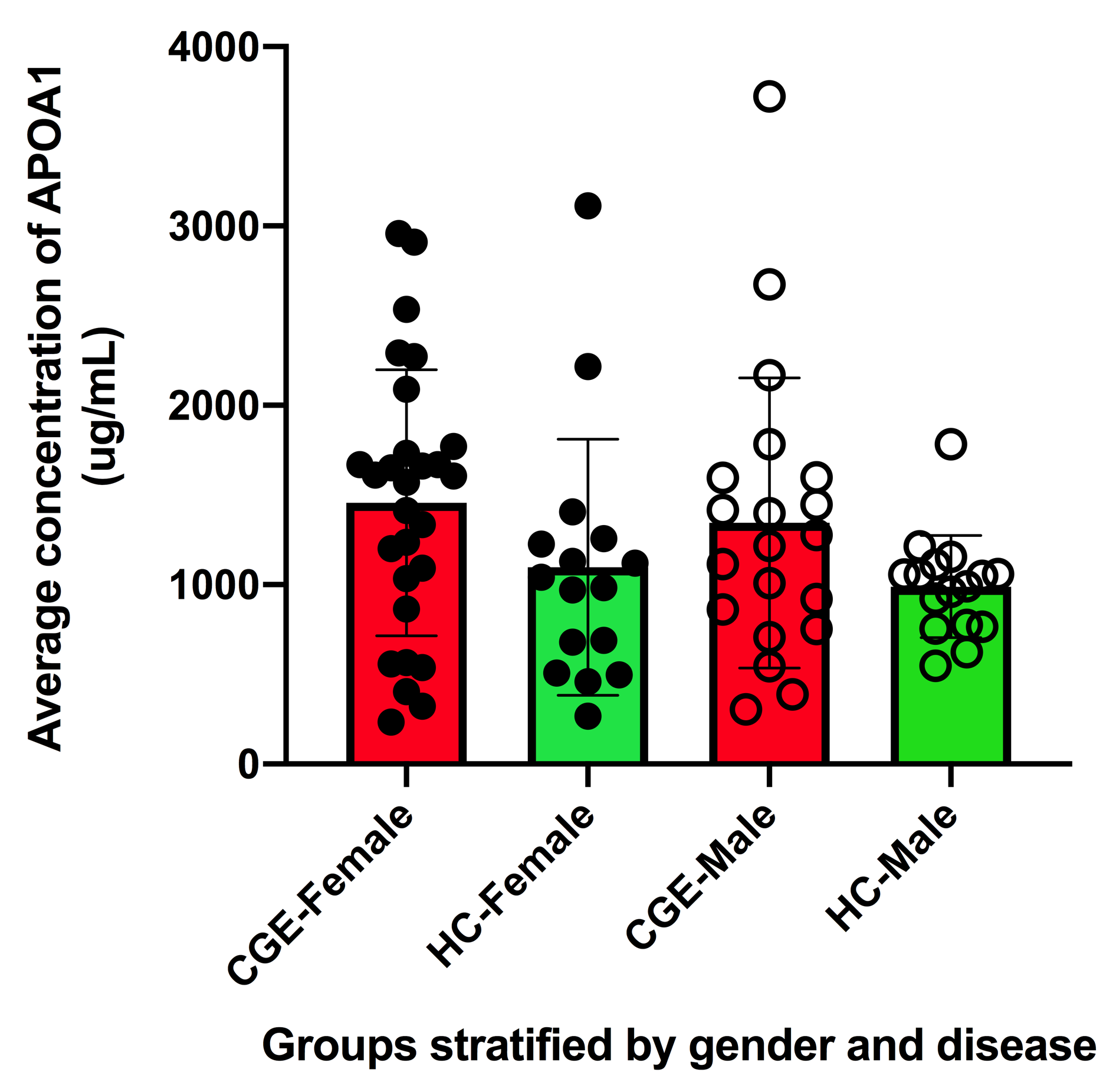

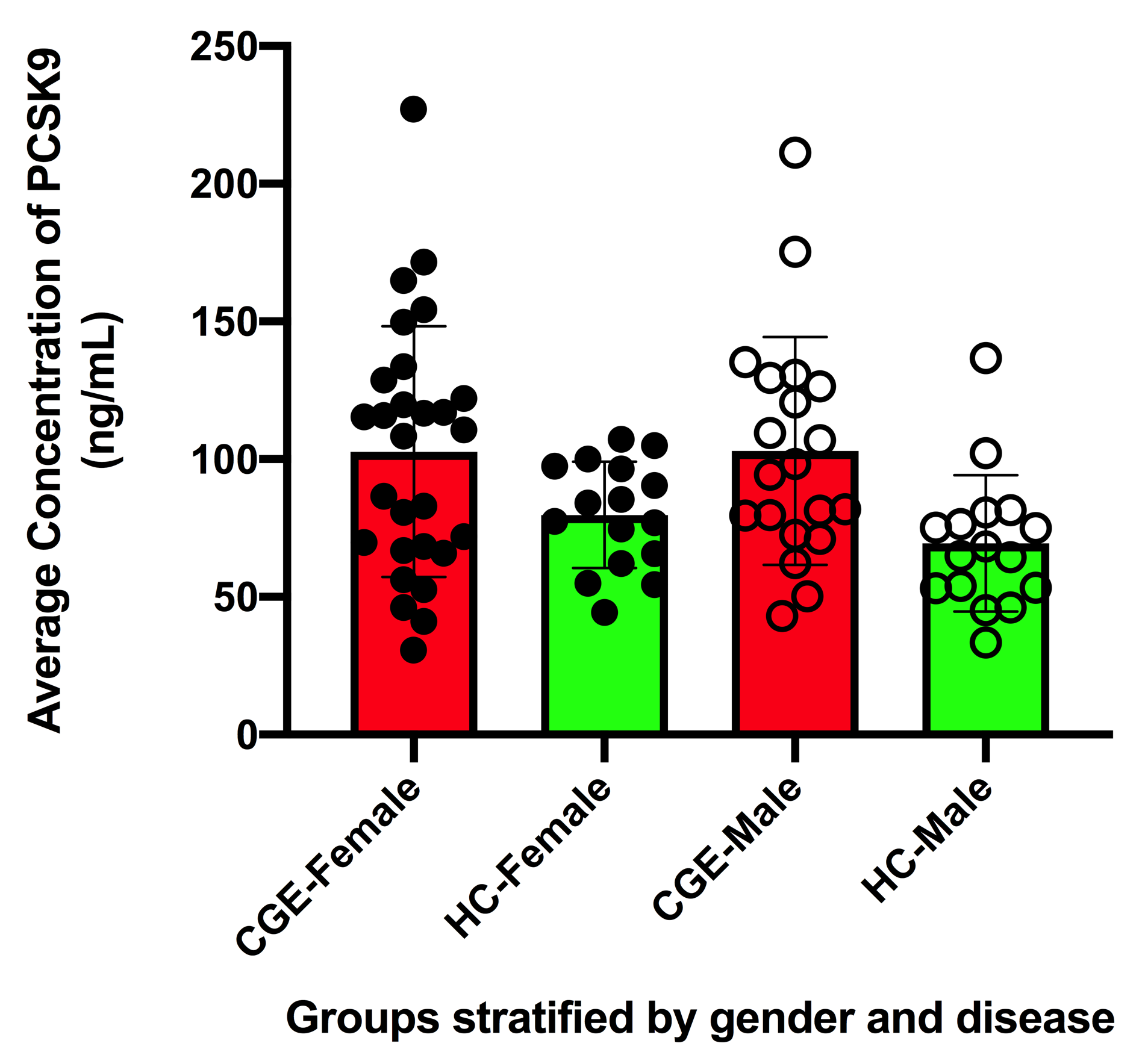

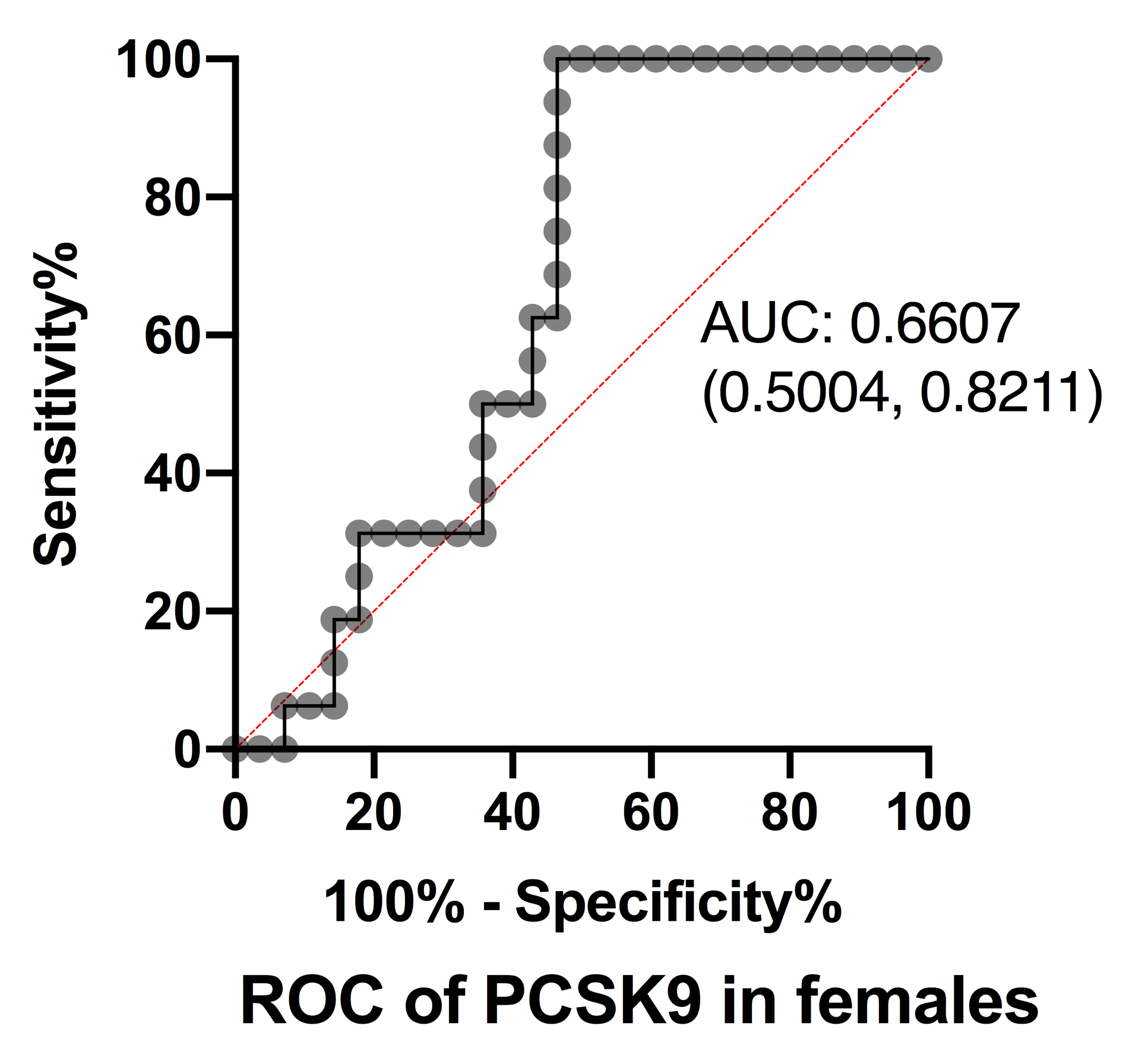

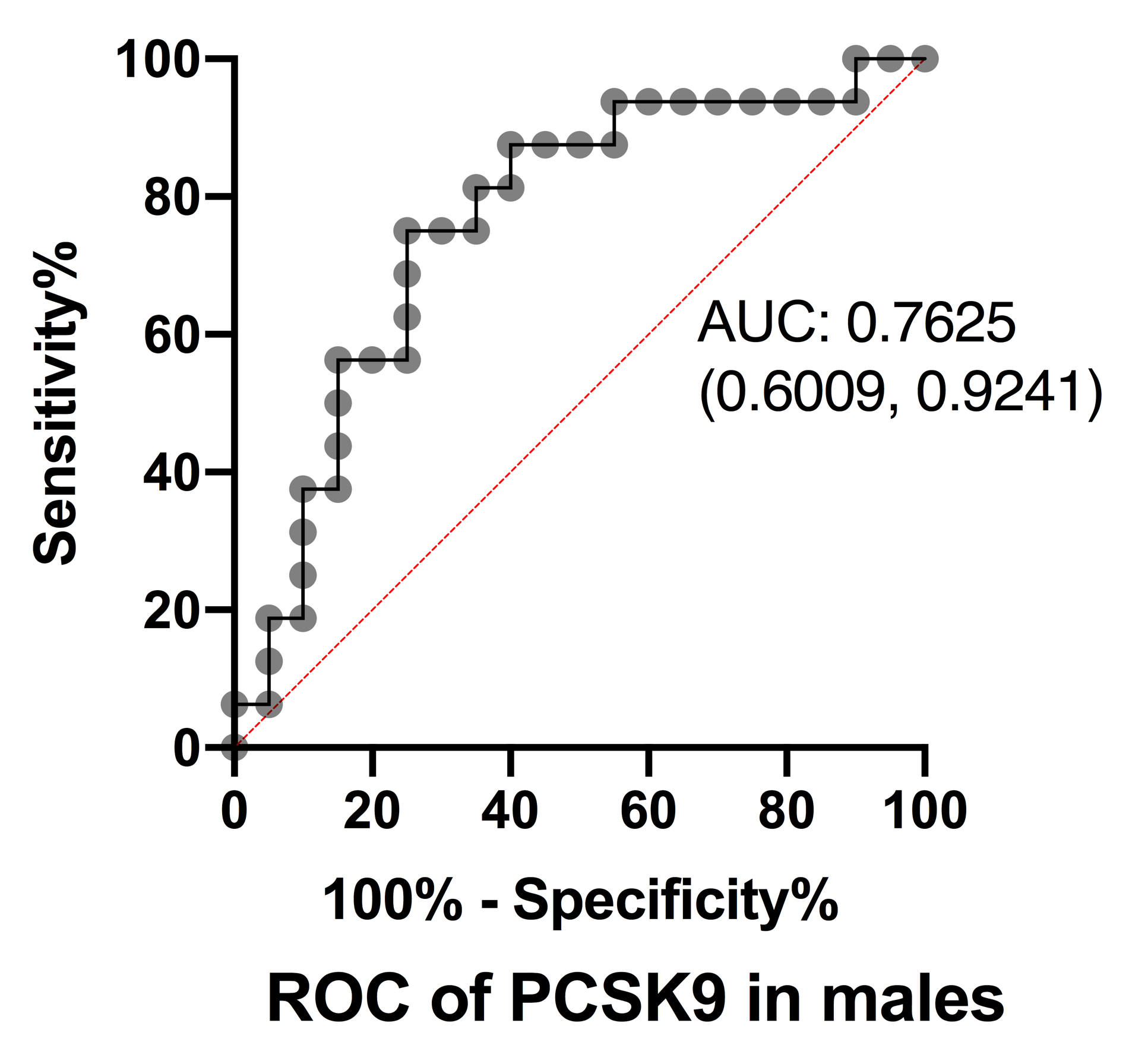

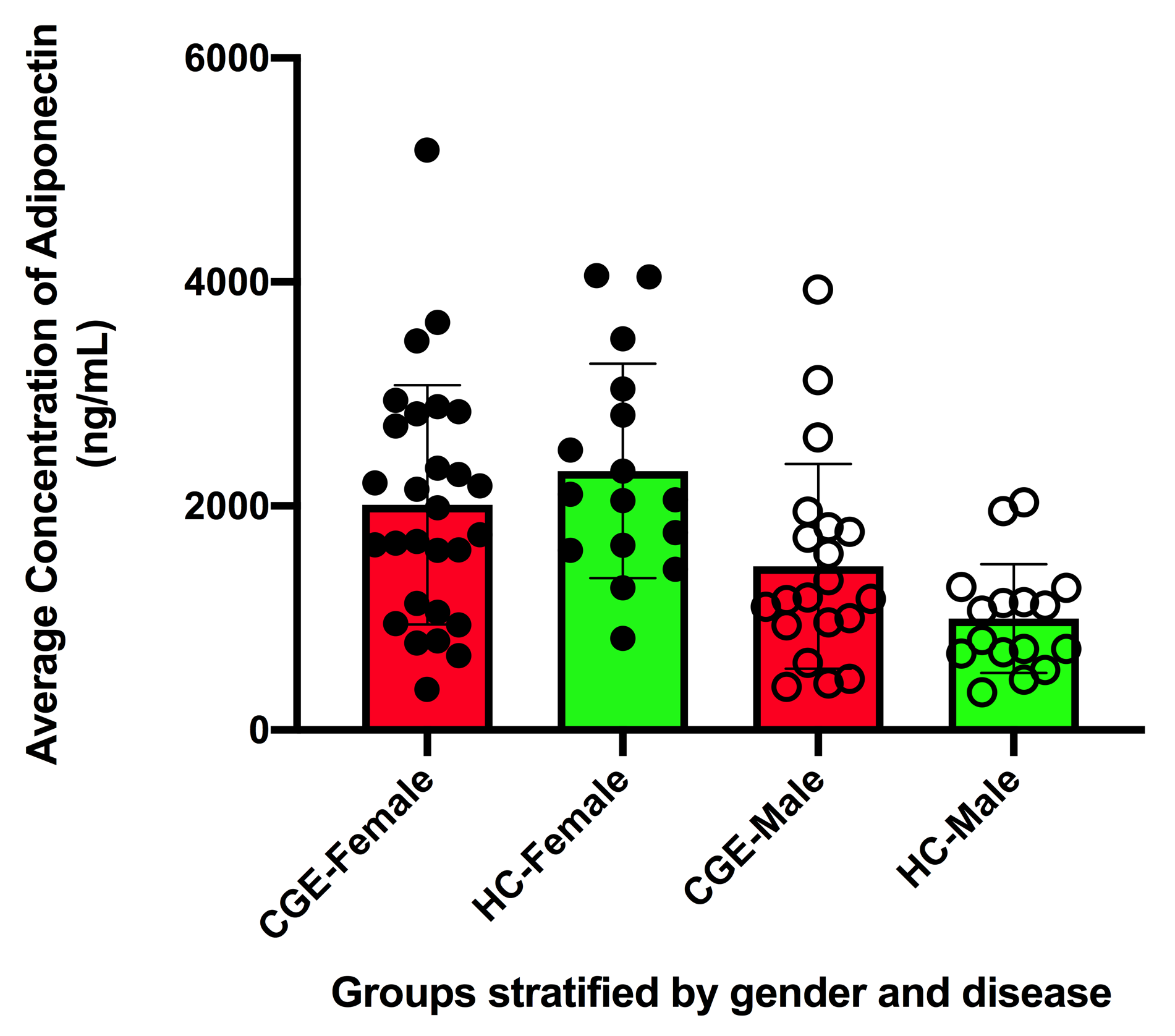

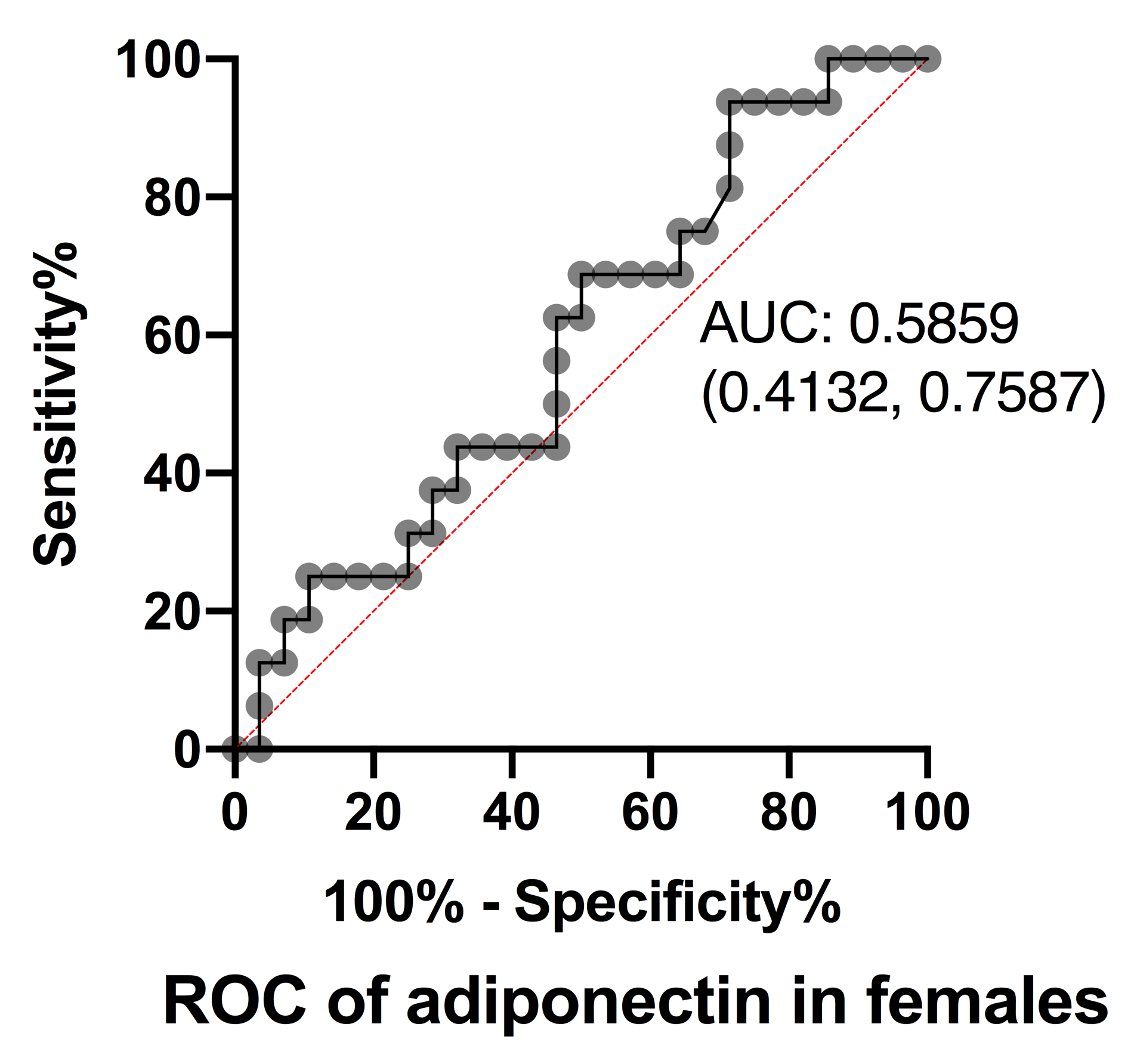

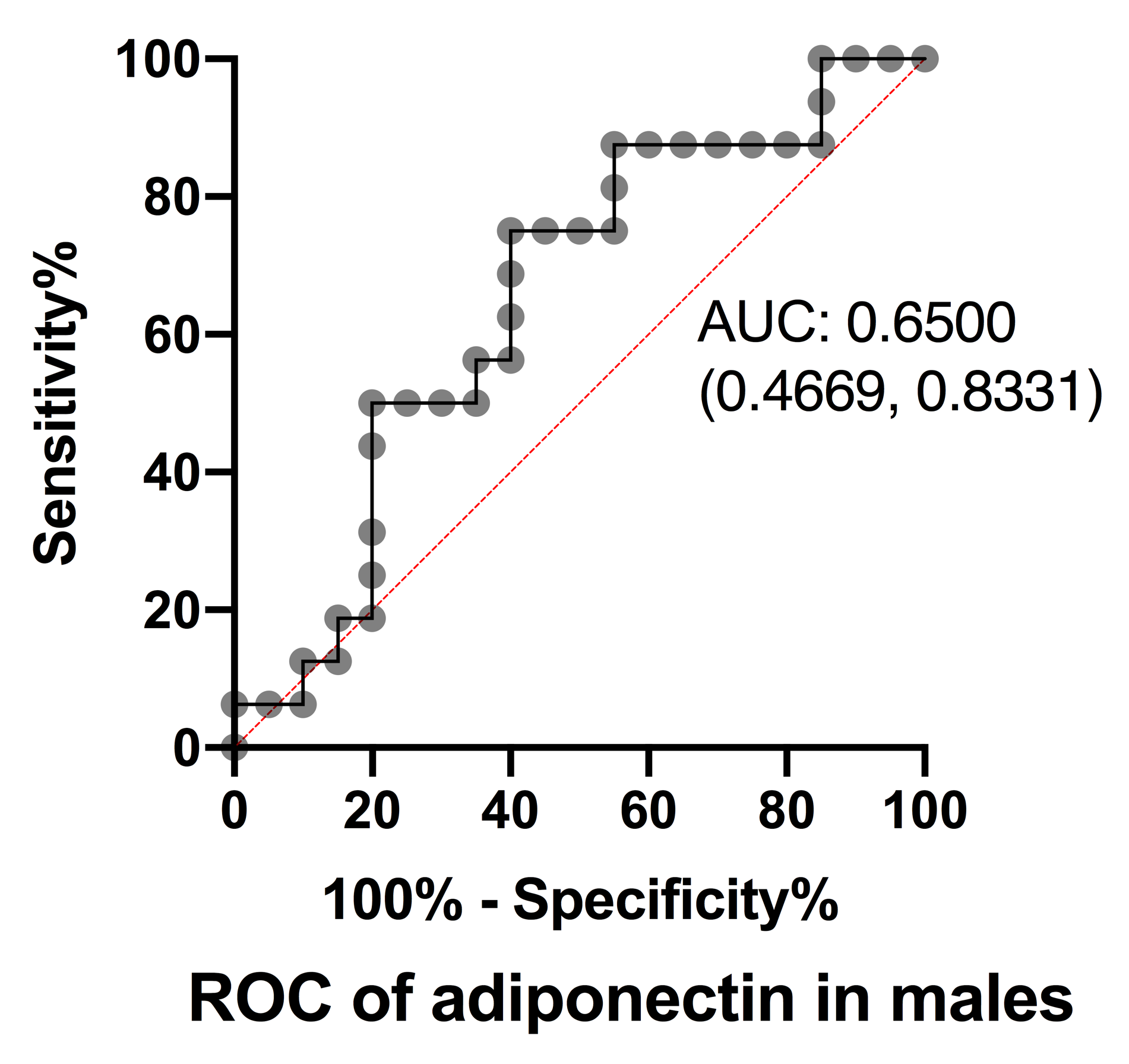


**Figure S2. ROC analysis and concentration comparison of**

**APOA1, APOC3, Adiponectin and PCSK9 between CGE patients and healthy controls**

**
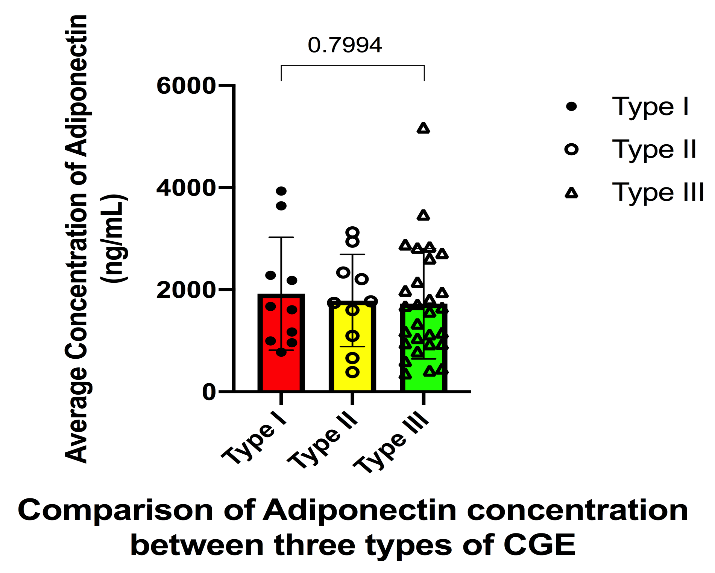

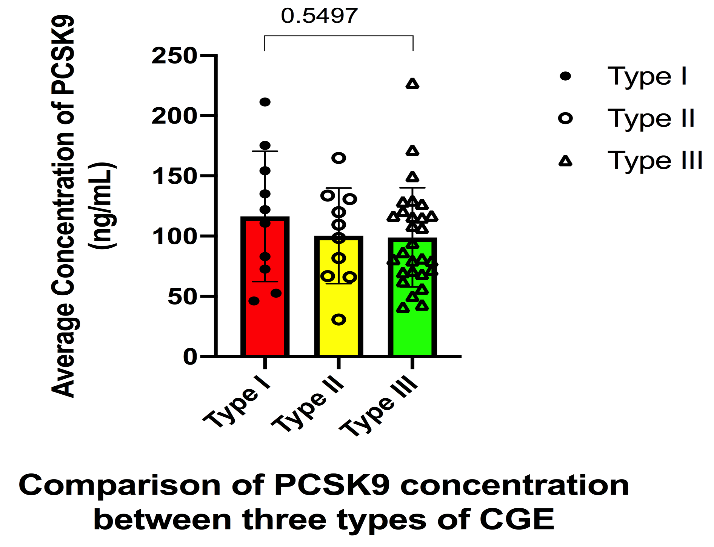
Figure S3. Comparison of APOA1, APOC3, Adiponectin and PCSK9**


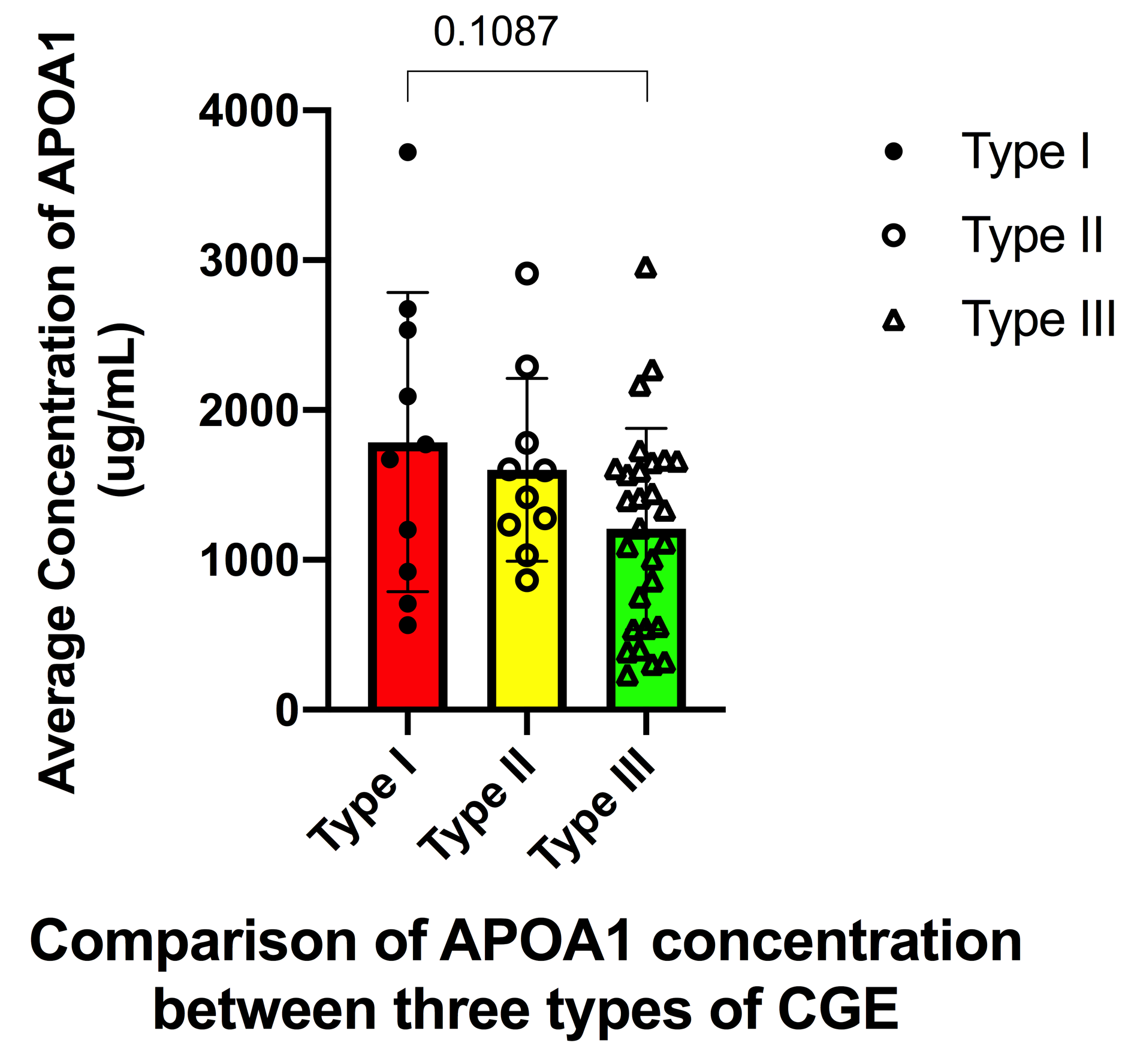

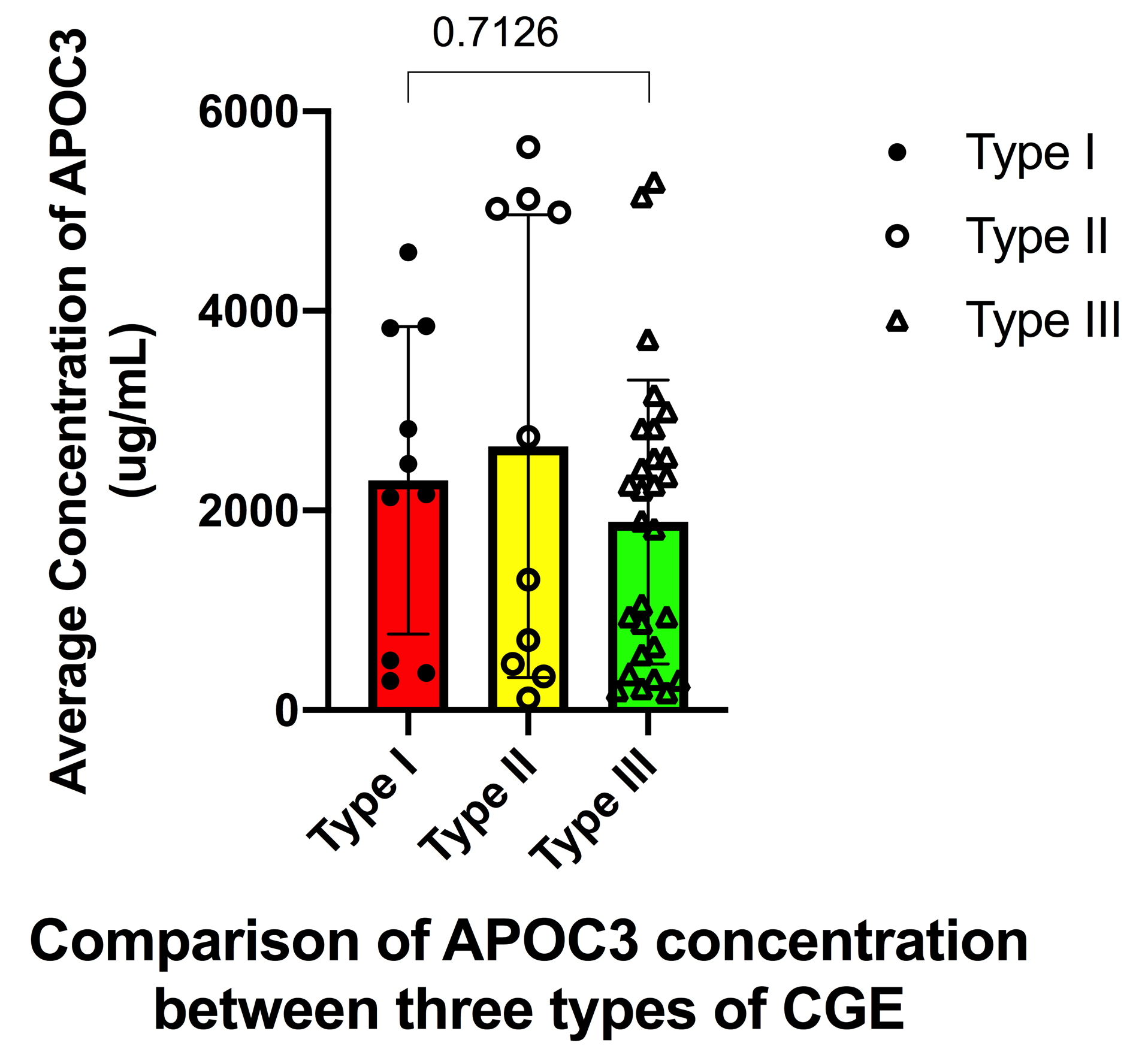


**concentration between different types of CGE**
